# Sex-determining region complements traditionally used in phylogenetic studies nuclear and chloroplast sequences in investigation of *Aigeiros* Duby and *Tacamahaca* Spach poplars (genus *Populus* L., Salicaceae)

**DOI:** 10.3389/fpls.2023.1204899

**Published:** 2023-10-04

**Authors:** Elena V. Borkhert, Elena N. Pushkova, Yuri A. Nasimovich, Marina V. Kostina, Natalia V. Vasilieva, Ramil A. Murataev, Roman O. Novakovskiy, Ekaterina M. Dvorianinova, Liubov V. Povkhova, Daiana A. Zhernova, Anastasia A. Turba, Elizaveta A. Sigova, Anastasiya V. Snezhkina, Anna V. Kudryavtseva, Nadezhda L. Bolsheva, George S. Krasnov, Alexey A. Dmitriev, Nataliya V. Melnikova

**Affiliations:** ^1^ Engelhardt Institute of Molecular Biology, Russian Academy of Sciences, Moscow, Russia; ^2^ State Environmental Protection Budgetary Institution of Moscow “Mospriroda”, Moscow, Russia; ^3^ Institute of Biology and Chemistry, Moscow Pedagogical State University, Moscow, Russia; ^4^ Tsitsin Main Botanical Garden, Russian Academy of Sciences, Moscow, Russia; ^5^ Faculty of Biology, Lomonosov Moscow State University, Moscow, Russia; ^6^ Moscow Institute of Physics and Technology, Moscow, Russia

**Keywords:** *Populus*, *Aigeiros*, *Tacamahaca*, poplars, intersectional hybrids, molecular phylogeny, sex-determining region, targeted deep sequencing

## Abstract

Members of the genus *Populus* L. play an important role in the formation of forests in the northern hemisphere and are used in urban landscaping and timber production. *Populus* species of closely related sections show extensive hybridization. Therefore, the systematics of the genus is rather complicated, especially for poplars of hybrid origin. We aimed to assess the efficiency of application of the sex-determining region (SDR) in addition to the nuclear and chloroplast genome loci traditionally used in phylogenetic studies of poplars to investigate relationships in sections *Aigeiros* Duby and *Tacamahaca* Spach. Targeted deep sequencing of NTS 5S rDNA, ITS, *DSH 2*, *DSH 5*, *DSH 8*, *DSH 12*, *DSH 29*, *6*, *15*, *16*, *X18*, *trnG-psbK-psbI*, *rps2-rpoC2*, *rpoC2-rpoC1*, as well as SDR and *ARR17* gene was performed for 379 poplars. The SDR and *ARR17* gene together with traditionally used multicopy and single-copy loci of nuclear and chloroplast DNA allowed us to obtain a clustering that is most consistent with poplar systematics based on morphological data and to shed light on several controversial hypotheses about the origin of the studied taxa (for example, the inexpediency of separating *P. koreana*, *P. maximowiczii*, and *P. suaveolens* into different species). We present a scheme of relationships between species and hybrids of sections *Aigeiros* and *Tacamahaca* based on molecular genetic, morphological, and geographical data. The geographical proximity of species and, therefore, the possibility of hybridization between them appear to be more important than the affiliation of species to the same section. We speculate that sections *Aigeiros* and *Tacamahaca* are distinguished primarily on an ecological principle (plain and mountain poplars) rather than on a genetic basis. Joint analysis of sequencing data for the SDR and chloroplast genome loci allowed us to determine the ancestors of *P.* × *petrovskoe* – *P. laurifolia* (female tree) × *P.* × *canadensis* (male tree), and *P.* × *rasumovskoe* – *P. nigra* (female tree) × *P. suaveolens* (male tree). Thus, the efficiency of using the SDR for the study of poplars of sections *Aigeiros* and *Tacamahaca* and the prospects of its use for the investigation of species of the genus *Populus* were shown.

## 1 Introduction

The genus *Populus* L. is distributed in the northern hemisphere from subtropical to boreal forests (DiFazio et al., 2011) and is one of the most economically and ecologically important genera of forest trees (Stettler et al., 1996). Poplar wood is used to produce plywood, pulp, paper, and biofuels (Kobolkuti et al., 2019). Species and interspecific hybrids of the genus *Populus* are widely used in landscaping cities and creating protective plantings because poplars have such valuable characteristics as fast growth, ecological plasticity, and ease of vegetative propagation (Bakulin, 2005). Poplars show high dust, smoke and gas resistance, and a huge leaf surface purifies the air of harmful impurities and absorbs a significant amount of CO_2_ (Kulagin, 1974; Jansson et al., 2010). In addition to their commercial value, poplars are ecologically important pioneers, colonizing disturbed and riverine ecosystems and contributing to the formation of natural forests in temperate and boreal regions, as well as in mountainous areas in the northern hemisphere (Schroeder et al., 2012).

The genus *Populus*, according to the Eckenwalder classification, includes 29 species in six sections: *Abaso*, *Turanga*, *Populus* (synonym: *Leuce*), *Leucoides*, *Aigeiros*, and *Tacamahaca* (Eckenwalder, 1996). Species of the genus *Populus* show extensive hybridization within sections as well as between closely related sections (Roe et al., 2014; Jiang et al., 2016). In this regard, the systematics of the genus *Populus* is rather complicated, and so far the researchers have not come to a consensus on the classification criteria, and the establishment of the taxonomic status of poplars of hybrid origin (both natural and cultivated) is particularly difficult (Cronk, 2005; Slavov and Zhelev, 2010; Schroeder et al., 2012; Mayorov et al., 2020). The question of the number of sections that should be distinguished in the genus *Populus* is also unresolved. For example, molecular genetic studies have shown that sections *Leucoides*, *Aigeiros*, and *Tacamahaca* should be considered not as separate sections but as a single subgenus, “Eupopulus” (Wang et al., 2020; Wang et al., 2022). Indeed, species of sections *Aigeiros* and *Tacamahaca* easily interbreed with each other, are connected by a powerful gene flow, and, in fact, have a common gene pool (at least, within Eurasia). Moreover, a common range of Eurasian poplars spreads from Western Europe to the Far East. Contacts between species in nature occur only at the borders of the habitats, i.e. “mixing” of genes occurs slowly. In this case, natural selection, despite gene flow, manages to maintain specificity of adaptive traits of each species, due to which it is possible to easily distinguish natural species morphologically (Nasimovich et al., 2019). In the study of cultivated poplars, identification is more difficult, but even in this case there is a factor supporting the discreteness of cultivars – the work of nurseries that propagate cultivars vegetatively. In this case, however, the control of varietal identity of seedlings is extremely important. When propagating poplars for use in urban landscaping, it is preferable to use only male clones that do not produce fluff (downy seeds) (Melnikova et al., 2019; Huang et al., 2022). However, it is impossible to determine the sex of a 1-2-year-old seedling by its phenotype; this can only be done after a few years when an adult tree begins to flower. In the cities of Central Russia, poplar was actively used in landscaping, but many female clones were mistakenly planted, which leads to annual problems with poplar fluff, because it collects and carries allergens, clogs drains and ventilation systems, is a fire hazard, and so on (Janković et al., 2021; Melnikova and Dmitriev, 2022). Genetic testing of seedlings to determine the sex would help not to repeat previous mistakes. In addition, when creating poplar hybrids for urban landscaping or industrial wood use, it is advisable to properly select parental species considering their adaptation to certain habitats (for example, representatives of the section *Aigeiros* are mostly plain poplars, and the section *Tacamahaca* includes mostly mountain poplars) (Nasimovich et al., 2019). Thus, understanding the evolutionary relationships of *Populus* species is important for basic research and breeding work (Wang et al., 2014).

A significant number of studies have been devoted to the search for reliable molecular markers for establishing phylogenetic relationships between poplar species – an analysis of various regions of nuclear DNA as well as sequences of the chloroplast genome was carried out (Schroeder et al., 2012; Wang et al., 2014; Alexandrov and Karlov, 2018; Zhou et al., 2018; Wang et al., 2022). The informativity for phylogenetic studies of poplars was noted for the sequences of non-transcribed intergenic spacers (NTS) of the 5S ribosomal RNA genes (rDNA) (Negi et al., 2002; Wilson, 2013; Alexandrov and Karlov, 2018) and internal transcribed spacers of rRNA genes (ITS) (Leskinen and Alström-Rapaport, 1999). At the same time, there are discrepancies between the phylogenetic trees obtained by analyzing the chloroplast and nuclear genome sequences of *Populus* species, which are usually attributed to their polyphyletic or hybrid origins (Hamzeh and Dayanandan, 2004; Wang et al., 2014; Liu et al., 2016; Zhou et al., 2018; Wang et al., 2022). In the analysis of chloroplast genome sequences, some accessions of the same species (a prime example is *P. nigra*) did not cluster together, but there was a significant association with geographic origin, which can be explained by recent hybridization (Wang et al., 2022). Analysis of different sequences allows clarification of various issues: chloroplast genome sequences showed great informativity for determining interspecific relationships in the genus *Populus*, including taxa difficult to systematize, while nuclear genome sequences were more effective for studying the deep phylogeny of *Populus* (Wang et al., 2022).

Poplars are dioecious plants, and another promising sequence for clarifying the systematic relationships of species and hybrids of the genus *Populus* is the sex-determining region (SDR). Sex chromosomes are actively used in phylogenetic studies (Parker et al., 2020; Navarro-Lopez et al., 2021). The species of sections *Aigeiros* and *Tacamahaca* are characterized by an XY sex-determination system (Gaudet et al., 2008; Pakull et al., 2009; Pakull et al., 2011; Kersten et al., 2014; Geraldes et al., 2015; Pakull et al., 2015; McKown et al., 2017), which allows the examination of poplar relationships in the male lineage. A series of studies made it possible to identify sex-associated DNA polymorphisms, determine the location and sequence of the SDR in various species of the genus *Populus*, and establish that sex is determined by the *ARABIDOPSIS RESPONSE REGLATOR 17* (*ARR17*) gene – when *ARR17* is turned on, female flowers develop, and when it is turned off – men ones (Gaudet et al., 2008; Yin et al., 2008; Pakull et al., 2009; Paolucci et al., 2010; Kersten et al., 2014; Geraldes et al., 2015; Pakull et al., 2015; McKown et al., 2017; Melnikova et al., 2019; Muller et al., 2020; Xue et al., 2020; Zhou et al., 2020; Kim et al., 2021; Melnikova et al., 2021; Yang et al., 2021). In species of sections *Aigeiros* and *Tacamahaca*, the SDR is localized on the end of chromosome 19, has a length of about 120 thousand nucleotides, and contains several genes, including *T-complex protein 1 subunit gamma* (*TCP*), *Chloride channel protein CLC-c* (*CLC*), and *DNA-methyltransferase 1* (*MET1*). Only in the SDR of male plants, there are partial repeats of the *ARR17* gene, from which small RNAs are synthesized, leading to the inactivation of *ARR17*, which is localized on the opposite to the SDR end of chromosome 19 (Xue et al., 2020; Zhou et al., 2020; Melnikova et al., 2021). Although the *ARR17* gene plays a key role in sex determination in the genus *Populus*, species of this genus differ in the sex-determination system and structure of the SDR. *P. euphratica* and *P. tremula* have an XY sex-determination system, however, in *P. tremula*, the SDR containing partial *ARR17* repeats is located in the peritelomeric region of chromosome 19, while in *P. euphratica*, it is located in the peritelomeric region of chromosome 14. Moreover, the SDR of these species is several hundred thousand nucleotides, differing significantly from the SDR of poplars of sections *Aigeiros* and *Tacamahaca* (Muller et al., 2020; Zhang et al., 2022). In *P. alba*, three copies of the *ARR17* gene are located in the peritelomeric region of chromosome 19 in female plants, whereas in male plants, *ARR17* is absent, indicating that *P. alba* has an ZW sex-determination system (Muller et al., 2020).

Previously, we showed that SDR polymorphisms can be effectively used in assessing the genetic proximity of *Aigeiros* and *Tacamahaca* species and hybrids based on whole genome sequencing data (Melnikova et al., 2021). The aim of this work was to evaluate the efficiency of using the SDR to establish the relationships of poplar species and hybrids of sections *Aigeiros* and *Tacamahaca* in combination with the nuclear and chloroplast genome sequences that have proven themselves in the study of the genus *Populus* systematics based on the target deep sequencing data.

## 2 Materials and methods

### 2.1 Plant material

The first part of the collection was formed from 222 samples of poplars of sections *Aigeiros* and *Tacamahaca* collected on the territory of Moscow (Russia). Plant material was gathered in different areas of the city to maximize the coverage of the existing genetic diversity. Samples were collected during flowering of poplar trees for accurate sex determination. The second part of the collection was formed from 157 samples of sections *Aigeiros* and *Tacamahaca*; the material was collected in 15 different regions of Russia: Moscow and Moscow Region, Orenburg Region, Novosibirsk Region, Altai Republic, Kemerovo Region, Republic of Khakassia, Krasnoyarsk Territory, Transbaikal Territory, Republic of Sakha (Yakutia), Jewish Autonomous Region, Khabarovsk Territory, Primorsky Territory, Sakhalin Region, and Magadan Region, as well as in natural areas of poplar species in 5 countries: Italy, Uzbekistan, Kazakhstan, Kyrgyzstan (Irkeshtam), Mongolia. Part of the accessions were herbarium samples from the collection of the Tsitsin Main Botanical Garden, Russian Academy of Sciences (MHA, Moscow, Russia). The resulting collection consisted of 379 accessions of poplars of sections *Aigeiros* and *Tacamahaca*. Affiliation of a poplar to a particular species/hybrid was carried out using a key for determining poplars (Mayorov et al., 2020). A complete list of accessions is given in **Supplementary Data 1**, and a description of poplar species and hybrids can be found in **Supplementary Data 2**.

Accessions from the section *Tacamahaca* were represented by the following species: *P. suaveolens* – 38 samples, *P. laurifolia* – 18 samples, *P. talassica* – 10 samples, *P. longifolia* – 6 samples (and 3 more samples with doubts in identification), *P. maximowiczii* – 8 samples, *P. simonii* – 8 samples, *P. koreana* – 5 samples, and *P. trichocarpa* – 4 samples. Accessions from the section *Aigeiros* were represented by the following species: *P. nigra* – 22 samples, *P. nigra* × *P. pyramidalis* – 3 samples, *P. afghanica* – 3 samples, and *P. deltoides* – 1 sample. The sample set also included the hybrids most commonly used in Moscow landscaping: *P.* × *sibirica* – 34 samples (and 27 more samples with doubts in identification), *P.* × *petrovskoe* – 57 samples (and 5 more samples with doubts in identification), *P.* × *rasumovskoe* – 30 samples (and 4 more samples with doubts in identification), *P.* × *canadensis* – 3 samples, as well as a rarely cultivated hybrid *P.* × *wobstii* (3 samples) and a natural Siberian hybrid *P.* × *irtyschensis* (7 samples). We also studied *P. nigra* × *P.* × *sibirica* – 8 samples, *P. deltoides* × (*P. laurifolia* × *P. suaveolens*) – 4 samples, *P. deltoides* × *P. suaveolens* – 1 sample. In 5 cases, more complex hybrids were examined. Five samples could not be identified reliably, and two possible affiliations were given for them. For 57 samples, the species affiliation was not established.

### 2.2 Description of the studied species and hybrids of poplar

The following is an enumeration of the studied taxa, along with a list of diagnostic features for poorly known taxa. We described in detail hybrid taxa for which mutually exclusive data are available in the literature on diagnostic traits and parental species. In these cases, we have to give detailed descriptions so that it is clear exactly what our genetic data refer to. For more details, see the full description of the studied taxa in **Supplementary Data 2**.

#### 2.2.1 Section *Aigeiros* Duby

Mainly plain poplars; petioles are long, strongly flattened laterally, without an adaxial groove, usually glabrous; leaf blades are rhombic or deltate, but, in some species, with rounded lateral edges; the maximum expansion of a leaf blade is very strongly shifted toward the base.

***P. afghanica*** C.K.Schneid.

Crown is spreading or pyramidal

Petioles are slightly pubescent or glabrous

Leaf blades are rhombic-like or deltate, but with rounded lateral edges

Note: morphologically close to *P. nigra*; however, it has certain features of “balsam” poplars

***P.* × *canadensis*** Moench [*P. deltoides* × *P. nigra*; cultivar]

Crown is spreading

Petioles are glabrous

Glands occur only on half of the leaves

Leaf blades are deltate or rhombic-like

***P. deltoides*** W.Bartram ex Marshall

Crown is spreading

Petioles are glabrous

Leaf blades are deltate

Glands are on most leaves

Note: leaves are particularly large

***P. nigra*** L. (*P*. *nigra* var. *nigra*)

Crown is spreading

Petioles are glabrous

Leaf blades are rhombic-like, less often deltate

Glands are usually absent or small

***P. nigra* × *P. pyramidalis*** Rozier (*P*. *nigra* var. *nigra* × *P. nigra* var. *italica* Munchh.; cultivar).

Crown is pyramidal

Petioles are slightly pubescent, less often glabrous

Leaf blades are rhombic-like, less often deltate, with slightly rounded edges

Glands are usually small or absent

Note: mixes with more southern *P. pyramidalis* Rozier; represented in the dendrograms under this name

#### 2.2.2 Section *Tacamahaca* Spach

Predominantly mountain poplars; petioles are terete (rounded in cross section) and shorter on average than those of “balsam” poplars, usually pubescent, with an adaxial groove; leaf blades are of various shapes (lanceolate, ovate, slightly cordate, etc.), but not rhombic-like or deltate; the maximum expansion of a leaf blade is in its middle or shifted toward base or apex.

***P. koreana*** Rehder

Axes of 1-2-year-old shoots are terete

Leaf blades are angular (not flat), matte, strongly leathery

Leaf blade apex is acuminate, tapering to a tip up to 5-6 mm long

Midrib is pubescent on the abaxial side

Capsules have 3 glabrous valves

***P. laurifolia*** Ledeb.

Axes of 1-2-year-old shoots are strongly grooved

Leaf blades are flat, matte, not leathery

Leaf blade apex is acute or attenuate, without a long tip

Midrib is glabrous

Capsules usually have 3 glabrous valves

***P. longifolia*** Fisch.

Axes of 1-2-year-old shoots are terete

Leaf blades are flat, matte, not leathery

Leaf blade apex is acute or attenuate, without a long tip

Midrib is glabrous

Capsules have 3 glabrous valves

Note: a medium-height tree; forms abundant sucker shoots; the color contrast of the adaxial and abaxial sides of leaf blades is maximal among the section *Tacamahaca* representatives; petioles are long

***P. maximowiczii*** Henry

Axes of 1-2-year-old shoots are terete

Leaf blades are flat, slightly glossy, slightly leathery

Leaf blade apex is acuminate, tapering to a tip up to 5-6 mm long

Midrib is pubescent on the adaxial and abaxial sides

Capsules have 3 glabrous valves

***P. simonii*** Carriere f. *fastigiata*

Axes of 1-2-year-old shoots are strongly grooved

Leaf blades are flat, matte, not leathery

Leaf blade apex is acute or obtuse, sometimes apiculate, with a 0.5-3 mm tip

Midrib is glabrous

Note: crown is pyramidal or wide pyramidal; leaf blades are obovate, always with attenuate base

***P. simonii *** Carriere f. *subpendula*

Leaf blades are flat, matte, not leathery

Leaf blade apex is acute, sometimes attenuate, without a long tip

Midrib is glabrous

Note: crown is semi-weeping; leaf blades are lanceolate (cuneate base and acute apex), sit on short shoots (brachyblasts)

***P. suaveolens*** Fisch.

Axes of 1-2-year-old shoots are terete

Leaf blades are flat, matte, not leathery

Leaf blade apex is acuminate, tapering to a tip up to 5-6 mm long

Midrib is glabrous

Capsules have 3 glabrous valves

***P. talassica *** Kom.

Axes of 1-2-year-old shoots are angular

Leaf blades are flat, matte, not leathery

Leaf blade apex is acute or attenuate

Midrib is glabrous

Capsules have 3 glabrous valves

***? P. trichocarpa*** Torr. et A.Gray ex Hook. [? *P.* × *trichocarpa* ‘Lettland’]

Here and further the sign «?» before a species/hybrid means that we have doubts in accurate identification of species affiliation

Axes of 1-2-year-old shoots are terete or grooved

Leaf blades are flat, matte, not leathery

Leaf blade apex is acute, without a narrow tip

Capsules have 3 pubescent valves

***P. × wobstii *** R.I.Schrod. (*P. laurifolia* and *P. longifolia*)

Axes of 1-2-year-old shoots are slightly grooved

Leaf blades are flat, matte, not leathery

Leaf blade apex is acute, without a narrow tip

Midrib is glabrous

Capsules have 3 glabrous valves

#### 2.2.3 Intersectional hybrids *Aigeiros* × *Tacamahaca*

This group includes the most widespread poplar hybrids (hybridogenic species, spontaneous hybrids, cultivars) of urban landscaping in Russia. Some of them are known under erroneous names in Russia and sometimes beyond its borders. These names may not coincide in Russia and other countries and may be different in the understanding of different authors within the same country. Therefore, we provide the main diagnostic features of these hybrids and sometimes complete descriptions (see also **Supplementary Data 2**).

The common features of these poplars are the following: petioles are long or medium, slightly flattened laterally, with a narrow and interrupted adaxial groove, usually slightly pubescent; leaf blades have diverse forms (lanceolate, ovate, cordate, etc., often rhombic-like); the maximum expansion of a leaf blade is strongly shifted toward the base.

***P. deltoides*** × ***(P. laurifolia *** × ***P. suaveolens)***

Crown is spreading

Axes of 1-2-year-old shoots are terete, angular, sometimes slightly grooved

Leaf blades are ovate or cordate, the length exceeds the width by 1.5 times on average

Leaf blade base is rounded, cuneate, or cordate

Leaf blade apex is attenuate

Capsules have 3 valves

***P. × irtyschensis *** Chang Y.Yang [*P*. × *berolinensis* nothovar. *irtyschensis* (Chang Y.Yang) C.Shang; *P. laurifolia* × *P. nigra* var. *nigra*]

Crown is spreading and irregular

Axes of 1-2-year-old shoots are angular or slightly grooved

Leaf blades are ovate-rhombic; the length exceeds the width by 1.5 times on average

Leaf blade base is rounded, cuneate, or irregular as in *P.* × *sibirica*

Leaf blade apex is acute or attenuate

Capsules have 2-3 valves

***P.*** × ***petrovskoe*** R.I.Schrod. ex Wolkenst. [In Russia, it is mentioned under the erroneous name of *P.* × *berolinensis* K.Koch; ? *P*. × *canadensis* × *P. laurifolia*]

Crown is spreading, regular, wide, becomes semi-pyramidal with age

Axes of 1-2-year-old shoots are angular or grooved

Leaf blades are ovate-rhombic, the length exceeds the width by 1.2 times on average

Leaf blade base is irregular: rounded at the petiole and broadly cuneate at a distance from it

Leaf blade apex is attenuate

Capsules have 3 valves

***P. × rasumovskoe *** R.I.Schrod. ex Wolkenst. [P. *nigra* var. *nigra* × *P. suaveolens*]

Crown is spreading, irregular, usually with hanging branch ends

Axes of 1-2-year-old shoots are terete

Leaf blades are lanceolate or ovate

Leaf blade base is rounded

Leaf blade apex is acuminate with a 1-1.5 cm tip

Note: the maximum expansion of a leaf blade is sometimes shifted toward the base because of the long tip

***P.*** × ***sibirica *** G.V.Krylov et G.V.Grig. ex A.K.Skvortsov [? *P. nigra* var. *nigra* × (*P. laurifolia* × *P. suaveolens*)]

Crown is spreading, irregular

Axes of 1-2-year-old shoots are terete or angular, rarely slightly grooved

Leaf blades are ovate-rhombic, the length exceeds the width by 1.5-2 times on average

Leaf blade base is irregular: rounded at the petiole and cuneate at a distance from it

Leaf blade apex is attenuate (1-2 cm tip)

Capsules have 2 or 2-3 valves

Note: leaves turn yellow in autumn and fall off earlier than in other analyzed intersectional poplar hybrids

### 2.3 DNA isolation for sequencing on the Illumina platform

For the accessions collected in Moscow, the 0.2 g leaf material was homogenized using a MagNA Lyser automatic homogenizer (Roche, Switzerland) in 1 ml of lysing modified CTAB buffer (100 mM Tris-HCl pH 8.0 (VWR Life Science, USA); 3% CTAB (VWR Life Science); 3M NaCl (Scharlab, Spain); 20 mM EDTA pH 8.0 (Promega, USA); 1% PVP K30 (PanReac AppliChem)) with 5 μl β-mercaptoethanol (BioRad, USA) and Solid-glass beads (Sigma-Aldrich, USA). The homogenate was incubated in a Gnom thermostat (DNA-Technology, Russia) at 65°C for 1 h, stirring every 20 min. Next, two consecutive purifications were performed with chloroform: an equal volume of chloroform (Acros Organics, USA) was added to the homogenate, and then centrifuged on a 5418R microcentrifuge (Eppendorf, Germany) for 10 min at 10,000 g and 4°C. The aqueous phase was then transferred to clean tubes, and two volumes of 96% alcohol were added to precipitate the DNA and incubated for 30 min at -20°C. Then, it was centrifuged for 15 min at 10,000 g and 4°C. After that, the supernatant was carefully removed without touching the precipitate. Next, the precipitate was washed with 70% alcohol and centrifuged for 5 min at 10,000 g and 4°C. The DNA precipitate was air dried for 5 min and dissolved in 100 µl of the DNA dilution buffer (Evrogen, Russia). The DNA concentration was estimated using a Qubit fluorometer (Thermo Fisher Scientific, USA). For the accessions provided from different regions of Russia, DNA was isolated with the classical CTAB method (Doyle and Doyle, 1987).

### 2.4 Preparation of DNA libraries for targeted deep sequencing on the Illumina platform

Preparation of DNA libraries included amplification of target nucleotide sequences and adding to each amplicon indexes unique to a sample and adapters required for sequencing. We designed primers for amplification of the SDR and *ARR17* gene loci, as well as applied primers previously used for amplification of single-copy nuclear DNA genes, namely *DSH 2*, *DSH 8*, *DSH 29*, *6*, *15*, *16*, *X18*, *DSH 5*, *DSH 12* (Du et al., 2014; Wang et al., 2014; Wang et al., 2019), chloroplast genome loci, namely *trnG‐psbK-psbI*, *rps2‐rpoC2*, *rpoC2‐rpoC1* (Schroeder et al., 2012; Huang et al., 2014; Zhou et al., 2018), and ITS (Hsiao et al., 1994) and NTS 5S rDNA (Falistocco et al., 2007). All primers were extended with sequences required for sample preparation and sequencing of DNA libraries on the Illumina platform as described in our previous works (Melnikova et al., 2019; Dmitriev et al., 2020; Novakovskiy et al., 2020; Povkhova et al., 2022) (a table with primers and a figure with the location of the amplified fragments in the SDR and *ARR17* gene are represented in **Supplementary Data 3**). Briefly, two sequential PCR were used to prepare DNA libraries: the first to amplify the target genome regions and add universal sequences to them (specific primers were used) and the second to add the sequences required for sequencing and double-indexes (Nextera XT v2 Illumina universal primers were used) as described previously (Melnikova et al., 2019; Dmitriev et al., 2020; Novakovskiy et al., 2020; Povkhova et al., 2022). The concentration and quality of the prepared DNA libraries were assessed using a Qubit fluorometer (Thermo Fisher Scientific) and an Agilent 2100 bioanalyzer (Agilent Technologies, USA). The obtained DNA libraries were diluted to a concentration of 4 nM. Sequencing of DNA libraries was performed on a MiSeq instrument (Illumina, USA) using the MiSeq Reagent Kit v3 (600 cycles) with reads of 300 nucleotides on each side.

### 2.5 Bioinformatics data analysis

The reads were trimmed from the 3’-end by quality, and adapter sequences were removed using Trimmomatic 0.39. The processed reads were then mapped to the regions of the reference genome of the male *P. trichocarpa* “Stettler 14” v1.1 (https://phytozome-next.jgi.doe.gov/info/PtrichocarpaStettler14 v1 1, (Hofmeister et al., 2020)) corresponding to the sequenced loci using BWA 0.7.17 with parameters for increased sensitivity due to probably low homology with the reference for some species/hybrids (Li, 2013). Next, the obtained BAM files were sorted using Samtools 1.10. The search for single-nucleotide polymorphisms (SNPs) and short insertions/deletions (InDels) was performed using freeBayes 1.3.2 (Garrison and Marth, 2012). Then, from the VCF files, the frequencies of SNP allele variants (variant allele frequency, VAF) were determined in different samples and pairwise Euclidean distances between samples were calculated based on the obtained VAF vectors. Clustering was performed with Ward’s method (ward.D2) in R 4.2.1. Visualization was performed using the ggtree and ggplot2 packages for R. Bootstrap analysis was performed using pvclust (Suzuki and Shimodaira, 2006). In our dataset, the majority of samples are hybrids of different species of sections *Aigeiros* and *Tacamahaca* (from 2 to 4-5 different parental species). In bootstrap analysis, they jump from cluster to cluster during subsampling because they have genetic similarity to different parental species. Thus, the bootstrapping method is impractical here to assess the reliability of the results. In this respect, it is better to consider the genetic distance matrices (calculated based on VAF) on the basis of which the clustering was performed.

## 3 Results

### 3.1 Results of targeted deep sequencing of 379 poplar accessions

For 379 poplar accessions of sections *Aigeiros* and *Tacamahaca*, 14 loci previously used in phylogenetic studies of poplars were sequenced, including NTS 5S rDNA, ITS, *DSH 2*, *DSH 5*, *DSH 8*, *DSH 12*, *DSH 29*, *6*, *15*, *16*, *X18*, *trnG-psbK-psbI*, *rps2-rpoC2*, *rpoC2-rpoC1*. In addition, loci of the sex-determining region (SDR) and *ARR17* gene, which determines sex in the genus *Populus* and whose partial repeats are located in the SDR, were sequenced. An average of about 500 reads were obtained for each sequenced amplicon of each sample. A search for polymorphisms was performed based on the sequencing data. The results are represented in **Supplementary Data 4**. We also evaluated the genetic proximity of the studied poplar accessions based on the identified polymorphisms. The results are represented in **Supplementary Data 5**. To visualize the data obtained on the genetic proximity of the studied poplar accessions, dendrograms were constructed based on the polymorphisms of the individual studied loci, as well as their combinations.

### 3.2 Analysis of 14 loci traditionally used in poplar phylogenetic studies

**Figure 1:**
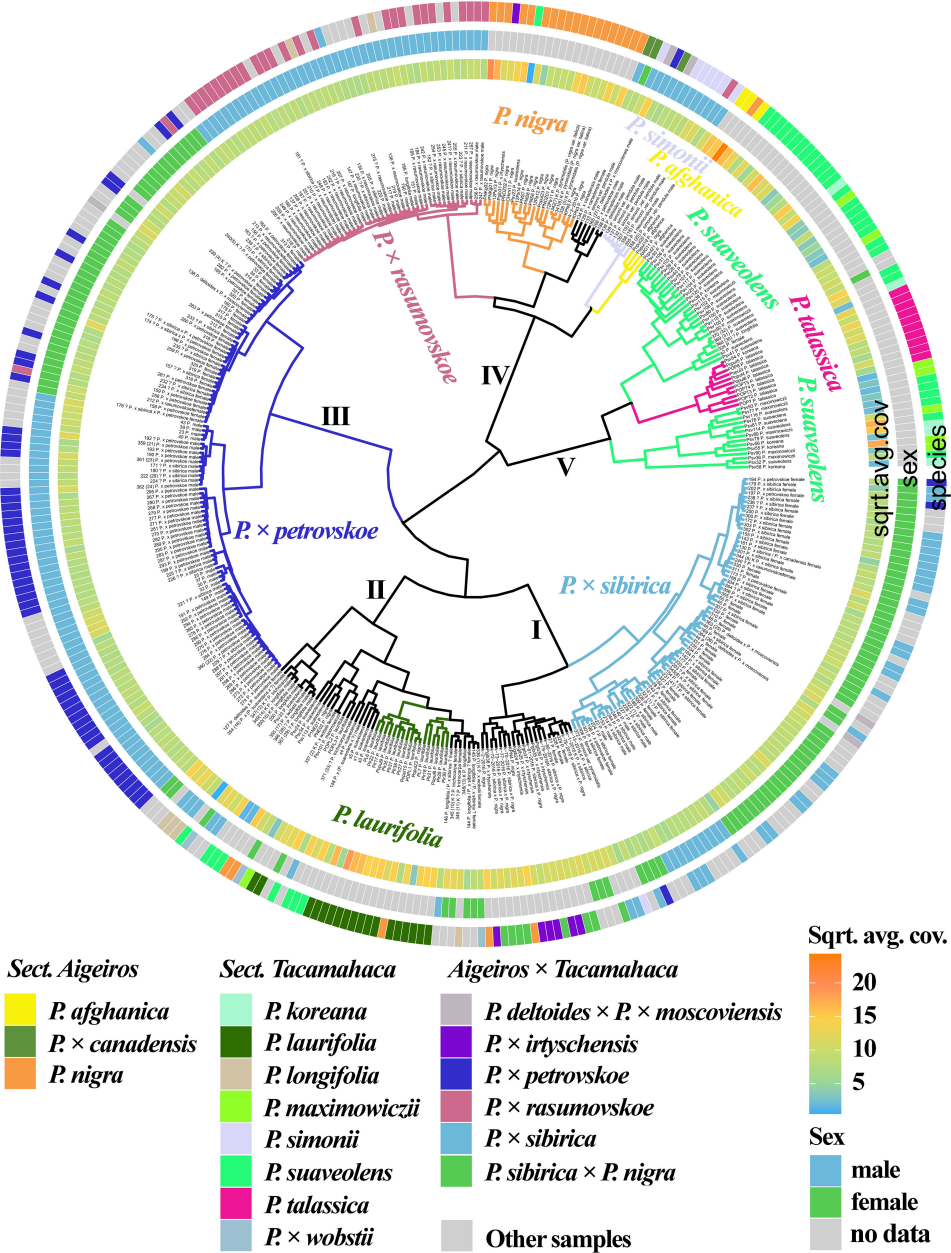
Dendrogram based on deep sequencing data for NTS 5S rDNA, ITS, *DSH 2*, *DSH 5*, *DSH 8*, *DSH 12*, *DSH 29*, *6*, *15*, *16*, *X18*, *trnG‐psbK-psbI*, *rps2-rpoC2*, and *rpoC2-rpoC1* sequences. Colors corresponding to species and hybrids mark only accessions for which there were no doubts in the morphological determination of the species affiliation.

**Figure 1** (**Supplementary Data 6A**, **8A**) shows the results of clustering poplar samples based on sequencing data for 14 loci previously used in phylogenetic studies of *Populus* species: NTS 5S rDNA, ITS, *DSH 2*, *DSH 5*, *DSH 8*, *DSH 12*, *DSH 29*, *6*, *15*, *16*, *X18*, *trnG‐psbK-psbI*, *rps2‐rpoC2*, *rpoC2-rpoC1* (White et al., 1990; Schmidt et al., 1994; Hsiao et al., 1995; Schroeder et al., 2012; Huang et al., 2014; Wang et al., 2014; Zhou et al., 2018; Wang et al., 2019). On the outer circle, the species affiliations of the studied 379 poplar accessions are marked with different colors. On the middle circle, blue and green colors indicate male and female genotypes, respectively. On the inner circle, data on read coverage for each examined sample are shown in the form of a heat scale.

A large cluster (I) was identified that included predominantly male and female samples of *P.* × *sibirica*, as well as hybrids in which one of the putative parental forms was *P.* × *sibirica*, all but one samples of *P.* × *irtyschensis*, and two samples of *P. nigra* (KPN4 and k-2-216). Another cluster (II) included predominantly male and female accessions of *P. laurifolia*, *P. longifolia*, some of *P. suaveolens*, *P.* × *wobstii*, and a few *P. nigra*. One more cluster (III) included the vast majority of *P.* × *petrovskoe* accessions, which were divided into two subclusters according to sex (male or female). The next cluster (IV) included almost all accessions of *P.* × *rasumovskoe*, forming a separate subcluster, and subclusters of *P. nigra*, *P. simonii*, and *P. afghanica*, as well as *P. deltoides* and its hybrids (including *P.* × *canadensis*). The latter cluster (V) combined subclusters of *P. suaveolens*, *P. koreana*, *P. maximowiczii*, and *P. talassica*. In all clusters, in addition to the listed accessions, there were also samples for which there were doubts in the accuracy of species identification (marked “?”) and samples for which determination of species affiliation on the basis of morphological analysis was not carried out.

The resulting dendrogram divided the samples quite well according to their species affiliation – thus, three clear clusters of hybrids (*P.* × *sibirica*, *P.* × *petrovskoe*, *P.* × *rasumovskoe*) and two clusters of “pure” or hybridogenic species were distinguished. The distinguished subclusters also corresponded to the division of samples according to the species affiliation. In addition, genetically related accessions were combined into clusters. For example, the *P.* × *rasumovskoe* cluster included most accessions of its probable parental form, *P. nigra*. *P. laurifolia*, which can be considered as a dynamic state of *P. suaveolens* under conditions of continuous gene flow from *P. nigra* (Nasimovich et al., 2019), formed one cluster with some of *P. suaveolens*. However, although we mostly saw a good division of accessions into small subclusters according to their species affiliation, a cluster could include several small clusters of accessions of different species interspersed with each other, as can be clearly seen for cluster II.

We also analyzed the dendrograms obtained from the sequencing data separately for each of the 14 studied loci and compared them with the dendrogram obtained from the data for all 14 loci. Low read coverage was observed for a number of samples for some sequenced loci (such samples are marked in dark blue in the heat scale of the inner circle); we do not draw any conclusions for them in the analysis described below.

#### 3.2.1 Analysis of multicopy loci of nuclear genome (NTS 5S rDNA, ITS)

We analyzed the dendrogram obtained on the basis of NTS 5S rDNA (chromosome 17) (Xin et al., 2020) (**Supplementary Data 6B**, **8B**), which showed efficiency in phylogenetic studies of poplars (Negi et al., 2002; Wilson, 2013; Alexandrov and Karlov, 2018). Samples of *P.* × *sibirica*, *P.* × *petrovskoe*, and *P.* × *rasumovskoe* formed separate groups. The *P.* × *sibirica* and *P.* × *rasumovskoe* subclusters merged into one cluster with the group of *P. suaveolens* samples, which is their probable ancestor. However, for many “pure” and hybridogenic species, the picture was ambiguous – they were all included in the same cluster, but did not form clear subclusters corresponding to individual species, like in the dendrogram based on 14 loci, although regularities corresponding to the phylogenetic relationships of poplars can be traced.

For another sequence frequently used in plant phylogenetic studies, ITS (chromosomes 8 and 14) (Xin et al., 2020), the clustering results were less consistent with the known information on poplar systematics and were inferior to NTS 5S rDNA in informativity (**Supplementary Data 6C**, **8C**). Thus, groups of *P.* × *petrovskoe* and *P.* × *rasumovskoe* were identified, but for *P.* × *sibirica* some samples were not in the main cluster of this hybrid, but in a cluster with different species. A number of accessions were grouped according to the species affiliation (e.g., the *P. simonii* subcluster), however, many samples of different species did not form separate subclusters but were interspersed with accessions of other species.

#### 3.2.2 Analysis of single-copy loci of nuclear genome (*DSH 2*, *DSH 5*, *DSH 8*, *DSH 12*, *DSH 29*, *6*, *15*, *16*, *X18*)

The *DSH 2* locus (chromosome 4) (Du et al., 2014) was sufficiently informative (**Supplementary Data 6D**, **8D**). Its analysis identified subclusters of *P.* × *rasumovskoe*, predominantly female samples of *P.* × *sibirica*, male samples of *P.* × *petrovskoe*, and female samples of *P.* × *petrovskoe*. However, female accessions of *P.* × *petrovskoe* were closer to female accessions of *P.* × *sibirica* than to male accessions of *P.* × *petrovskoe*. Some of the male samples of *P.* × *sibirica* clustered with the female samples of this hybrid, while others formed a separate distant subcluster. Subclusters of mainly samples of *P. nigra*, *P. talassica*, *P. laurifolia*, *P. simonii*, or *P. suaveolens* (together with *P. koreana* and *P. maximowiczii*, which can be considered as *P. suaveolens*) were also identified. However, some of the samples, like in the NTS 5S rDNA and ITS analyses, did not fall into the subclusters of the species to which they belong, but found themselves in other subclusters. In addition, subclusters were formed from samples of different species.

Analysis based on the *DSH 8* locus (chromosome 18) (Du et al., 2014) allowed us to distinguish a number of sample groups according to their taxonomic affiliation (**Supplementary Data 6E**, **8E**). Subclusters comprising predominantly accessions of one species/hybrid were identified for *P.* × *rasumovskoe*, *P.* × *sibirica*, *P.* × *petrovskoe*, *P. nigra*, and *P. simonii*. However, often, individual samples of the same species were not grouped together, but turned out to be in remote clusters. A striking example of such a pattern is *P. talassica*, which divided into groups.

When analyzed by the *DSH 29* locus (chromosome 14) (Wang et al., 2014), subclusters of mostly samples of the same species/hybrid were formed for *P.* × *rasumovskoe*, *P.* × *petrovskoe* (male and female plants were divided into two subclusters, the male subcluster also included samples of *P.* × *sibirica* male plants), *P. nigra*, and *P.* × *sibirica* (female plants) (**Supplementary Data 6F**, **8F**). For most other species, however, the division into groups according to taxonomic affiliation was unclear; species from different sections were grouped in the same cluster. For example, *P. simonii* (*Tacamahaca*) samples were in a subcluster with the majority of *P. nigra* (*Aigeiros*) samples; samples of *P. laurifolia*, *P. talassica*, *P. suaveolens* (together with *P. koreana* and *P. maximowiczii*) (*Tacamahaca*) and *P. nigra* (*Aigeiros*) formed mixed subclusters.

The analysis for gene *6* (chromosome 12) (Wang et al., 2019) identified clusters of the majority of samples of *P.* × *rasumovskoe*, *P.* × *petrovskoe* (divided into two subclusters according to sex), or *P.* × *sibirica* (**Supplementary Data 6G**, **8G**). However, separation of samples into subclusters corresponding to species was revealed only for small groups of accessions; there was no clear division according to taxonomic affiliation.

The gene *15* (chromosome 15) (Wang et al., 2019) analysis identified a cluster of *P.* × *rasumovskoe*, while *P.* × *petrovskoe* and *P.* × *sibirica* samples formed a mixed cluster (**Supplementary Data 6H**, **8H**). In addition, a cluster of the majority of *P. nigra* samples was identified, but several *P. nigra* samples were in other clusters. Samples of most species formed mixed clusters, which in some cases had isolated subclusters consisting of a small number of accessions of one species. Species from sections *Aigeiros* and *Tacamahaca* were often grouped in the same cluster.

The analysis of gene *16* (chromosome 3) (Wang et al., 2019) allowed us to distinguish subclusters of mainly *P.* × *rasumovskoe* or *P.* × *petrovskoe* (male trees) samples (**Supplementary Data 6I**, **8I**). A subcluster of the majority of *P. nigra* samples was identified, which also included *P.* × *irtyschensis* and *P.* × *canadensis* accessions. A subcluster of *P. simonii* samples was also distinguished. In addition, a cluster of a mixture of *P.* × *petrovskoe* and *P.* × *sibirica* female accessions was revealed. In some cases (e.g., for *P. talassica*, *P. suaveolens* (together with *P. koreana* and *P. maximowiczii*)), there were isolated small subclusters of samples of the same species, but often accessions of different species formed mixed subclusters.

The analysis of the *X18* locus (chromosome 15) (Wang et al., 2019) identified a cluster of *P.* × *petrovskoe* with a division into male and female genotypes (**Supplementary Data 6J**, **8J**). This cluster also included subclusters of *P. nigra* samples, which is the putative ancestor of *P.* × *petrovskoe*. Samples of *P.* × *rasumovskoe* and *P.* × *sibirica* were in one cluster and appeared to be mixed with each other. A subcluster of *P. simonii*, several subclusters of *P. suaveolens*, *P. nigra*, and *P. laurifolia* were identified, but a significant number of subclusters consisted of a mixture of samples of different taxa, including species from different sections.

The analyses based on *DSH 5* (chromosome 12) (Du et al., 2014) and *DSH 12* (chromosome 2) (Du et al., 2014) were low-informative (**Supplementary Data 6K, L**, **8K, L**). The division of samples into clusters/subclusters according to taxonomic affiliation was poorly pronounced.

#### 3.2.3 Joint analysis of 11 loci of nuclear genome

**Figure 2:**
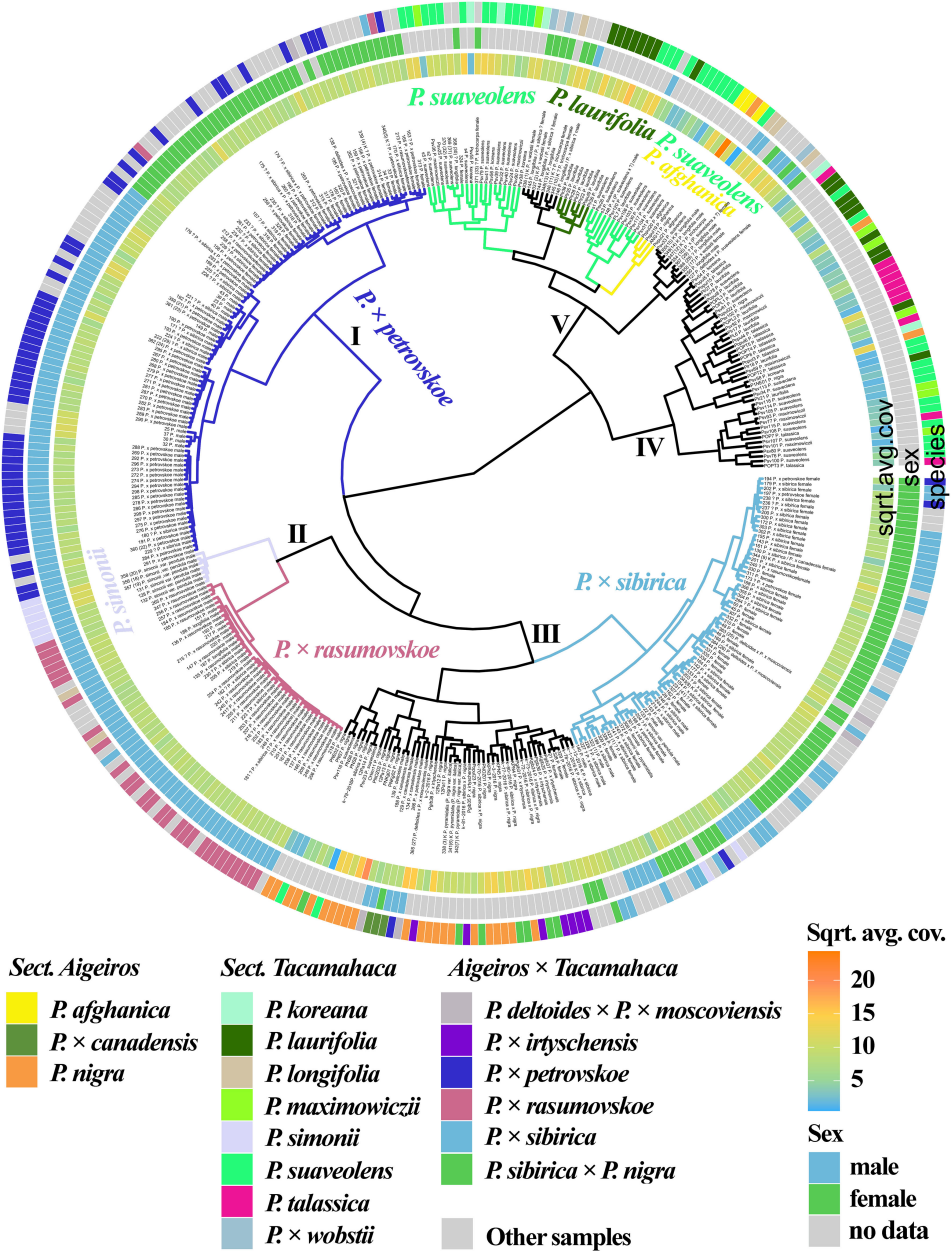
Dendrogram based on deep sequencing data for NTS 5S rDNA, ITS, *DSH 2*, *DSH 5*, *DSH 8*, *DSH 12*, *DSH 29*, *6*, *15*, *16*, and *X18* sequences. Colors corresponding to species and hybrids mark only accessions for which there were no doubts in the morphological determination of the species affiliation.

Based on the analysis of the NTS 5S rDNA, ITS, *DSH 2*, *DSH 5*, *DSH 8*, *DSH 12*, *DSH 29*, *6*, *15*, *16*, *X18* sequences (**Figure 2**, **Supplementary Data 6M**, **8M**), the *P.* × *petrovskoe* cluster (I) was isolated, with the samples divided into two subclusters according to sex. The *P.* × *rasumovskoe* subcluster was also distinguished and formed one cluster (II) with the subcluster of *P. simonii*. Samples of *P.* × *sibirica* formed a separate subcluster. A subcluster of the vast majority of *P. nigra* and *P.* × *irtyschensis* samples, all samples of *P. nigra* × *P.* × *sibirica* and *P.* × *canadensis*, and a single sample of *P. deltoides* merged into one cluster (III) with the *P.* × *sibirica* subcluster. Samples of *P. suaveolens* and *P. laurifolia* were found in two clusters (IV and V) and formed subclusters or interspersed with each other or with accessions of other species. *P. longifolia*, *P. afghanica*, and *P.* × *wobstii* were in cluster V, while *P. talassica* samples were in cluster IV. Clusters IV and V also contained one *P. nigra* accession. Thus, samples of different species were combined into small groups, but groups of different species alternated with each other – no large isolated subclusters were formed according to taxonomic affiliation.

#### 3.2.4 Analysis of chloroplast loci (*trnG‐psbK-psbI*, *rps2‐rpoC2*, *rpoC2-rpoC1*)

Based on the *trnG‐psbK-psbI* (Schroeder et al., 2012) analysis (**Supplementary Data 7A**, **8N**), *P.* × *rasumovskoe* samples were separated into a distinct cluster with the majority of *P. nigra* samples, which is considered one of the probable parental species of *P.* × *rasumovskoe* (Mayorov et al., 2020). The cluster of the majority of *P.* × *petrovskoe* samples also included a number of samples of *P. laurifolia*, a probable parental species of *P.* × *petrovskoe* (Mayorov et al., 2020). *P*. *talassica*, *P*. *afghanica*, *P*. *simonii*, and *P*. *suaveolens* formed subclusters (several groups) according to the species affiliation. Samples of *P.* × *sibirica* did not form a separate cluster but mixed with samples of other species, including *P. longifolia*, *P. suaveolens*, *P. laurifolia*, *P.* × *wobstii*, *P.* × *irtyschensis*, and several samples of *P. nigra*. The *trnG-psbK-psbI* sequence proved to be the most informative of the chloroplast loci we studied.

The *rps2‐rpoC2* (Schroeder et al., 2012) analysis (**Supplementary Data 7B**, **8O**) isolated the *P.* × *rasumovskoe* cluster, which also included most *P. nigra* samples. Subclusters of *P. suaveolens* (together with *P. koreana* and *P. maximowiczii*), *P. talassica*, and *P. simonii* were distinguished. No isolated clusters were identified for *P.* × *petrovskoe* and *P.* × *sibirica*; these accessions alternated with each other as well as with accessions of *P. laurifolia*, *P.* × *wobstii*, and *P.* × *irtyschensis*.

Based on the *rpoC2‐rpoC1* (Schroeder et al., 2012) analysis (**Supplementary Data 7C**, **8P**), a cluster of *P.* × *rasumovskoe* was identified, which also contained several samples of *P. nigra* and single accessions of other species. *P.* × *petrovskoe* accessions formed a large cluster, in which they were interspersed with samples of *P.* × *sibirica*, as well as samples of many other studied species, without forming subclusters according to taxonomic affiliation. In most cases, there was no grouping of accessions according to belonging to a particular species and section. This locus proved to be of little informative value.

**Figure 3:**
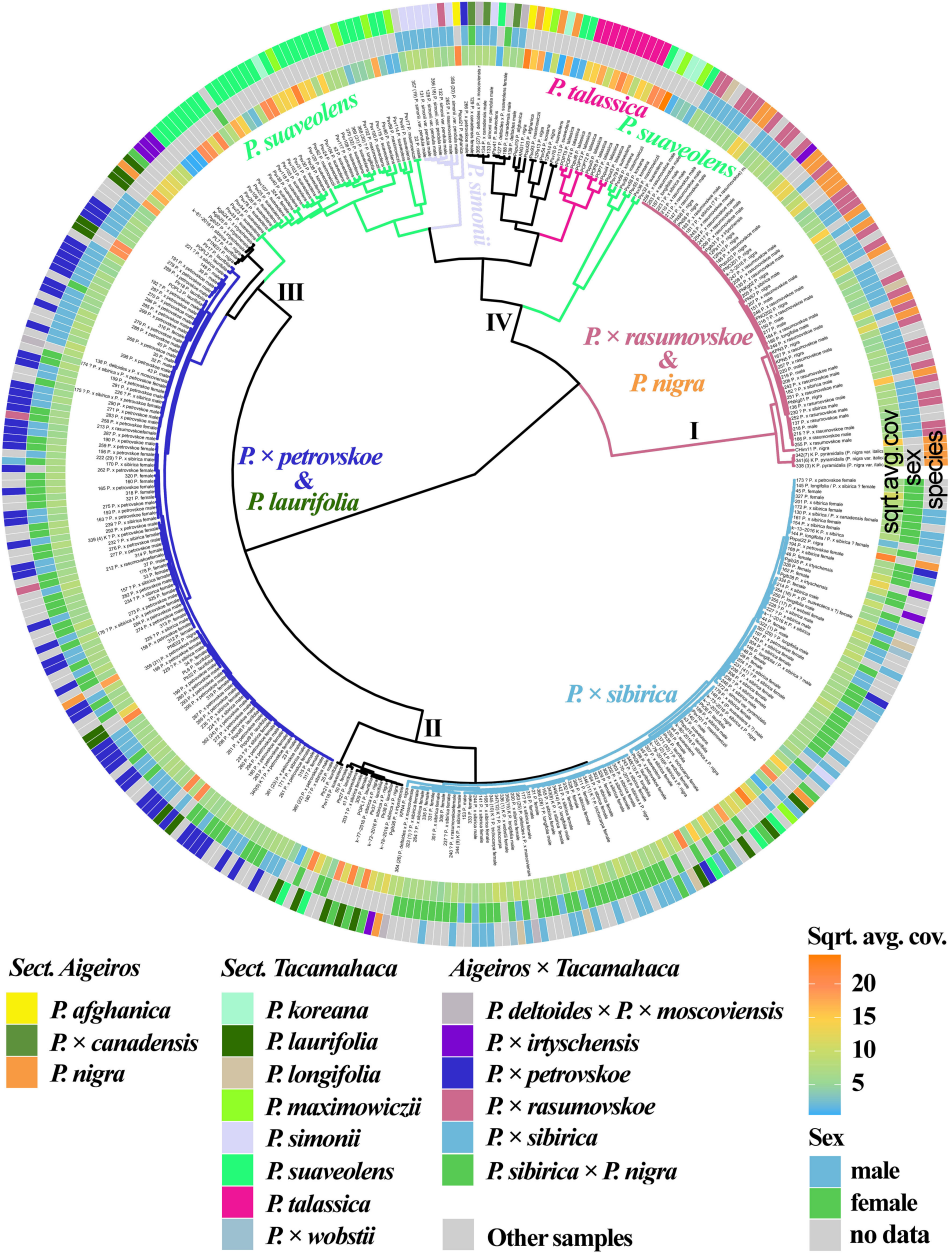
Dendrogram based on deep sequencing data for *trnG‐psbK-psbI*, *rps2-rpoC2*, and *rpoC2-rpoC1* sequences. Colors corresponding to species and hybrids mark only accessions for which there were no doubts in the morphological determination of the species affiliation.

The results based on the analysis of three chloroplast genome loci (*trnG-psbK-psbI*, *rps2-rpoC2*, *rpoC2-rpoC1*) simultaneously are shown in **Figure 3** (**Supplementary Data 7D**, **8Q**). *P.* × *rasumovskoe* formed one cluster (I) with the majority of *P. nigra* samples. It can be assumed that the female parental form for *P.* × *rasumovskoe* was *P. nigra* rather than the second probable ancestor, *P. suaveolens* (Mayorov et al., 2020). Cluster II included *P.* × *sibirica* samples which were mixed with several accessions of *P. suaveolens* (together with *P. koreana* and *P. maximowiczii*), significant number of *P. laurifolia*, *P. longifolia*, and *P.* × *wobstii* accessions, as well as several accessions of *P. nigra* and *P* × *irtyschensis*. Cluster III with most *P.* × *petrovskoe* samples also included a number of samples of *P. laurifolia*, which along with *P.* × *canadensis* is the probable parental species of *P.* × *petrovskoe* (Mayorov et al., 2020). Therefore, the probable female parental form for *P.* × *petrovskoe* was *P. laurifolia* rather than another putative ancestor, *P.* × *canadensis.* An analysis of the similarity of *P.* × *rasumovskoe* and *P.* × *petrovskoe* accessions and their probable parental forms based on genetic distances also showed that *P. nigra* was a more likely female ancestor of *P.* × *rasumovskoe* than *P. suaveolens* (the average genetic distance between accessions of *P.* × *rasumovskoe* and *P. nigra* was 0.7, and that between accessions of *P.* × *rasumovskoe* and *P. suaveolens* was 2.6), and *P. laurifolia* was a more likely female ancestor of *P.* × *petrovskoe* than *P.* × *canadensis* or another representative of the section *Aigeiros* (the average genetic distance between *P.* × *petrovskoe* and *P. laurifolia* samples was 0.5, and that between samples of *P.* × *petrovskoe* and *P.* × *canadensis*/*P. nigra* was 2.2) (**Supplementary Data 5**). Several accessions of *P. suaveolens* were also included in cluster III. Cluster IV combined subclusters of *P. suaveolens* (together with *P. koreana* and *P. maximowiczii*), *P. simonii*, and *P. talassica*, as well as accessions of *P. deltoides* and its hybrids (including *P.* × *canadensis*), *P. afghanica* and several *P. nigra* accessions, which were interspersed with each other.

Thus, the analysis by individual loci used in phylogenetic studies of *Populus* species, for a number of studied samples of sections *Aigeiros* and *Tacamahaca*, corresponded to the views on phylogenetic relationships in the genus *Populus*, but was significantly less informative than the joint analysis by 14 loci at once.

### 3.3 Analysis of the SDR and the *ARR17* gene

**Figure 4:**
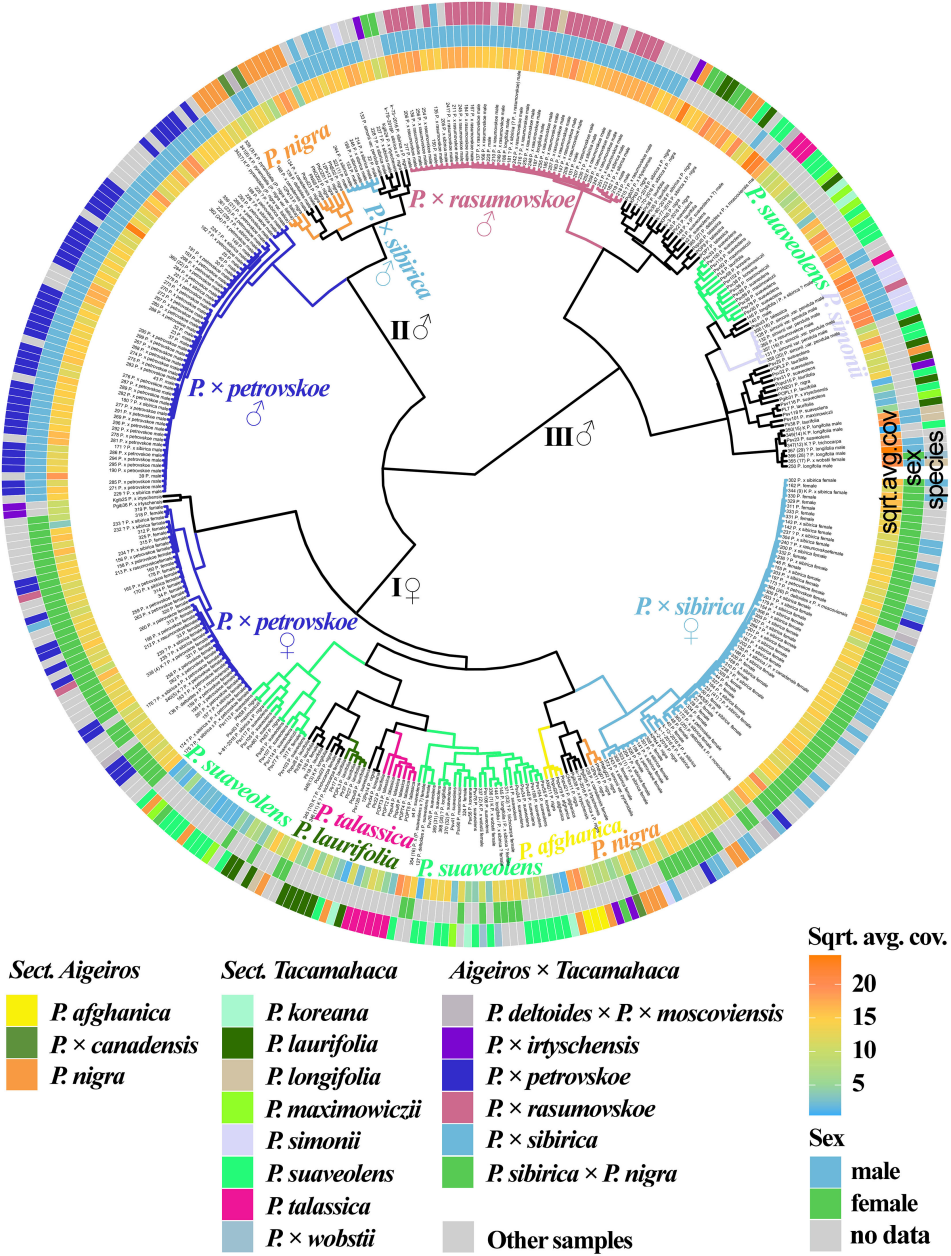
Dendrogram based on deep sequencing data for the SDR sequences. Colors corresponding to species and hybrids mark only accessions for which there were no doubts in the morphological determination of the species affiliation.

We also analyzed the genetic proximity of poplars based on the sequencing of the loci of the SDR localized on chromosome 19 (**Figure 4**, **Supplementary Data 7E**, **8R**). It should be explained that, for poplars of sections *Aigeiros* and *Tacamahaca*, the part of the SDR comprising partial repeats of the *ARR17* gene is present only in male plants, and another part is present in plants of both sexes, but differs significantly in polymorphisms (Zhou et al., 2020). The samples were divided into three large clusters, one of which (I) included only female plants and the other two (II and III) included only male plants (the only exception was sample 355(17) *P.* × *wobstii*). A subcluster of male samples of *P.* × *petrovskoe* was clearly distinguished in cluster II. A subcluster of a significant number of samples of *P. nigra* and *P. nigra* × *P. pyramidalis*, most male samples of *P.* × *sibirica*, as well as *P. deltoides* and *P.* × *canadensis*, was also separated. This subcluster merged with the *P.* × *petrovskoe* subcluster into one cluster (II). Based on these data, we may assume that in the male samples of *P.* × *petrovskoe* and *P.* × *sibirica*, the male parental form was a representative of the section *Aigeiros* (according to the morphological data, this is most likely *P.* × *canadensis* for *P.* × *petrovskoe* and *P. nigra* for *P.* × *sibirica* (Mayorov et al., 2020)). The *P.* × *rasumovskoe* subcluster was identified in cluster III. Most male poplars of the section *Tacamahaca*, including *P. suaveolens*, which is the probable parental form of *P.* × *rasumovskoe*, were located in the same cluster with *P.* × *rasumovskoe*. Based on the data obtained, we can assume that *P. suaveolens* was the male parental species of *P.* × *rasumovskoe*. Analysis of the similarity of *P.* × *rasumovskoe* and *P.* × *petrovskoe* samples and their probable parental forms based on genetic distances also showed that *P. suaveolens* is a more probable male ancestor of *P.* × *rasumovskoe* than *P. nigra* (the average genetic distance between samples of *P.* × *rasumovskoe* and *P. suaveolens* in the male cluster was 1.7, and that between samples of *P.* × *rasumovskoe* and *P. nigra* in the male cluster was 2.3), and *P.* × *canadensis* or another representative of the section *Aigeiros* is a more likely male ancestor of *P.* × *petrovskoe* than *P. laurifolia* (the average genetic distance between *P.* × *petrovskoe* and *P.* × *canadensis*/*P. nigra* of the male cluster was 1.8, and that between *P.* × *petrovsko*e and *P. laurifolia* of the male cluster was 2.6) (**Supplementary Data 5**). Besides, in cluster III of male samples, a subcluster was identified that included a significant group of interspersed *P. suaveolens*, *P. koreana*, and *P. maximowiczii*, indicating their genetic similarity. A subcluster of *P. simonii* was also revealed in cluster III. Thus, cluster III included male samples of *P.* × *rasumovskoe*, *P. suaveolens* (including *P. maximowiczii* and *P. koreana*), *P. simonii*, *P. laurifolia*, *P. talassica*, and *P. longifolia* which were absent in cluster II of male samples. Within group of male genotypes (clusters II and III), several accessions of *P. nigra* were revealed in cluster III, but most *P. nigra* samples were in cluster II.

In cluster I of female plants, a subcluster of the majority of *P.* × *sibirica* samples was separated, which was close to the subcluster of several samples of *P. nigra* and *P. afghanica*. A subcluster of female samples of *P.* × *petrovskoe* was also distinguished, which, unlike the male samples of this hybrid, was significantly distant from the cluster of *P.* × *sibirica*. In addition, the cluster of female accessions included subclusters of accessions of one species, namely *P. laurifolia* and *P. talassica*, and two subclusters of mainly *P. suaveolens* (including *P. maximowiczii* and *P. koreana*). However, several samples of *P. nigra* and *P.* × *wobstii* were also in subclusters of *P. suaveolens*.

The data obtained for the SDR significantly complement the information on the phylogenetic relationships of the studied species and hybrids of sections *Aigeiros* and *Tacamahaca*, allowing us to determine the ancestors of hybrids in the male lineage, as well as clearly separate samples into male and female ones.

We also performed clustering based on the data obtained for the *ARR17* gene (chromosome 19) (**Supplementary Data 7F**, **8S**). A subcluster of the majority of *P. nigra* samples was distinguished. A subcluster of most female and male samples of *P.* × *sibirica*, which was located in the same cluster as the *P. nigra* subcluster, was also identified. *P.* × *rasumovskoe* samples formed a separate subcluster. *P.* × *petrovskoe* divided into two clusters according to sex. A large subcluster consisted mainly of *P. longifolia*, two distant subclusters of *P. suaveolens* (including *P. maximowiczii* and *P. koreana*), and subclusters of *P. talassica* and *P. simonii* were also distinguished. Samples of *P. afghanica* formed a subcluster close to the *P. deltoides* and *P.* × *canadensis* subcluster, as well as the *P. talassica* subcluster. The *ARR17* analysis allowed us to differentiate samples of many species and hybrids into separate subclusters and was quite informative.

### 3.4 Joint analysis based on 14 loci traditionally used in poplar phylogenetic studies, SDR, and *ARR17* gene

**Figure 5:**
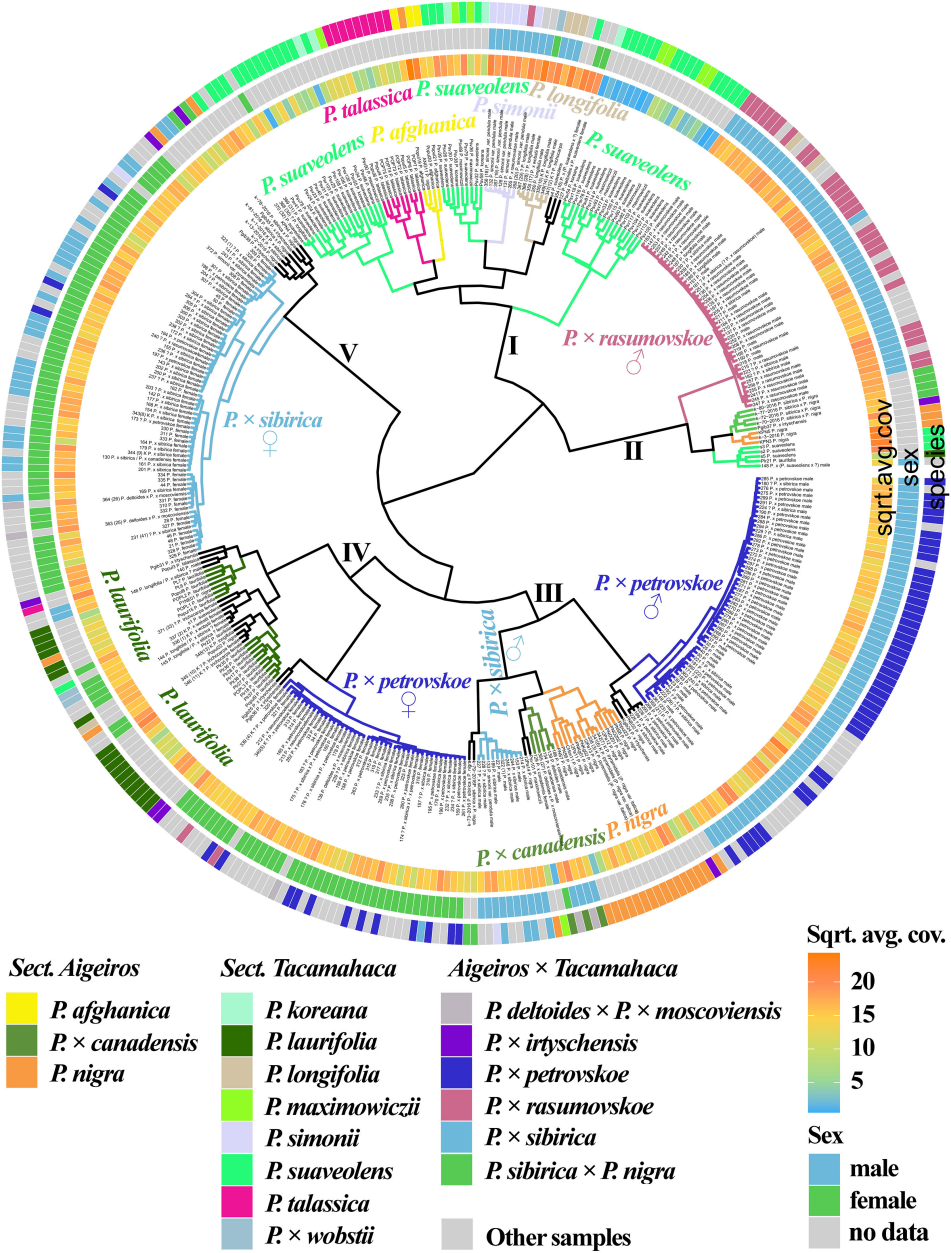
Dendrogram based on deep sequencing data for NTS 5S rDNA, ITS, *DSH 2*, *DSH 5*, *DSH 8*, *DSH 12*, *DSH 29*, *6*, *15*, *16*, *X18*, *trnG‐psbK-psbI*, *rps2-rpoC2*, *rpoC2-rpoC1*, SDR, and *ARR17* sequences. Colors corresponding to species and hybrids mark only accessions for which there were no doubts in the morphological determination of the species affiliation.

**Figure 5** (**Supplementary Data 7G**, **8T**) shows a dendrogram constructed on the basis of all the sequencing data we obtained, namely 14 loci that were previously used in phylogenetic studies of *Populus* and loci of the SDR and *ARR17* gene. Five large clusters were identified. Cluster I included mostly species. Subclusters of cluster I included mainly samples of only one species, namely *P. talassica*, *P. afghanica*, *P. simonii*, and *P. longifolia* (in this subcluster *P.* × *wobstii* and *P. trichocarpa* was located). Three more subclusters were also distinguished, in each of which samples of *P. suaveolens*, *P. maximowiczii*, and *P. koreana* were mixed. This supports the hypothesis that *P. koreana* and *P. maximowiczii* should not be distinguished as species or even subspecies, but should be assigned to *P. suaveolens* (Skvortsov and Belyanina, 2006). The subcluster of *P. afghanica* was merged with the subcluster of *P. talassica*. According to some hypotheses, *P. afghanica* can be regarded as a hybridogenic species derived from *P. nigra* and *P. talassica* (see **Supplementary Data 2** for details), and the results of the genetic analysis support the relationship between *P. afghanica* and *P. talassica*. Cluster II included samples of *P.* × *rasumovskoe*, as well as several samples of *P. suaveolens*, *P.* × *sibirica* × *P. nigra*, and *P. nigra*. All samples in this cluster for which the sex was determined were males. Cluster III was represented by predominantly male samples of *P.* × *petrovskoe*, which formed a separate subcluster. This cluster also included a significant number of *P. nigra* accessions, accessions of *P.* × *canadensis* (the probable ancestor of *P.* × *petrovskoe*), and several male *P.* × *sibirica* accessions that formed separate subclusters. In cluster IV, female samples of *P.* × *petrovskoe* and most samples of its probable ancestor *P. laurifolia* formed separate subclusters. Cluster V was represented mainly by female genotypes of *P.* × *sibirica*. In the joint analysis, subclusters of individual species and hybrids were distinguished quite well, the picture was clearer than in the analysis of individual loci/groups of loci. This dendrogram most accurately reflected the known data on the phylogenetic relationships of species and hybrids of sections *Aigeiros* and *Tacamahaca* and provided an opportunity to test a number of controversial hypotheses about the origin of the studied poplars.

Thus, the analysis by multicopy and single-copy nuclear DNA sequences, chloroplast DNA sequences, and the SDR loci simultaneously was the most informative and corresponded to the views on the systematics of the studied species and hybrids on the basis of morphological characteristics. Groups of samples of the same species/hybrid were well differentiated into separate subclusters. The results of targeted deep sequencing also shed light on a number of hypotheses derived from the morphological analysis, including the genetic proximity of *P. suaveolens*, *P. koreana*, and *P. maximowiczii*, as well as the relatedness of *P. talassica* and *P. afghanica*. The performed genetic analysis made it possible to effectively separate difficult for identification interspecific hybrids *P.* × *rasumovskoe*, *P.* × *petrovskoe*, and *P.* × *sibirica* that grow on the territory of Moscow. In addition, comparison of the results of the analysis by chloroplast DNA and the SDR allowed us to determine which species was the male parental form and which was the female parental form for male samples of *P.* × *rasumovskoe*, *P.* × *petrovskoe*, and *P.* × *sibirica*.

### 3.5 Clarification of poplar hybrid constitution based on targeted deep sequencing data

In our sample set, accessions of poplar species, especially collected in natural areas, were identified accurately enough on the basis of morphological features. However, for samples collected on the territory of Moscow, there were some difficulties with identification on the basis of morphological characteristics, which were especially evident for accessions of hybrid origin. First of all, this problem is related to cardinal pruning of trees, which is often used in cities, including to control poplar fluff. After such pruning, morphological features typically used for identification of hybrids may be distorted for some time, in particular, shape of crown, presence of shoots and leaves characteristic to a hybrid. As a result, in our sample set there were poplar accessions whose species affiliation could not be established, as well as poplar accessions whose species affiliation was doubted. Taking into account the fact that our genetic analysis allowed us to differentiate well the samples of different species and hybrids, we set the task to determine the species affiliation of those samples for which estimation of species affiliation by morphological features was difficult or was not carried out. Such analysis turned out to be especially relevant for probable hybrids collected mainly in Moscow.

In the dendrogram based on all sequenced loci (**Figure 5**, **Supplementary Data 7G**, **8T**), the *P.* × *rasumovskoe* subcluster contained 13 accessions with unknown or doubtful species affiliation: 150, 151, 181, 182, 215, 216, 217, 218, 219, 220, 223, 230, 241. When analyzed separately by the SDR (**Figure 4**, **Supplementary Data 7E**, **8R**) and by the set of 14 loci used in phylogenetic studies of poplars (**Figure 1**, **Supplementary Data 6A**, **8A**), these samples were also in the *P*. × *rasumovskoe* cluster, and not in other clusters. It is worth noting that the analysis of the genetic distances between the accessions (**Supplementary Data 5**) showed that all samples of *P.* × *rasumovskoe* were genetically very close, and samples from their cluster with uncertain species affiliation were also close to them. It can be assumed that they belong exactly to *P.* × *rasumovskoe*, and accessions of this hybrid distributed in Moscow are apparently descendants of the same clone. In addition, the *P.* × *rasumovskoe* group included accessions 186 and 187 classified as *P. longifolia* according to morphological characteristics. However, according to the SDR loci (**Figure 4**, **Supplementary Data 7E**, **8R**), the 3 chloroplast genome regions (**Figure 3**, **Supplementary Data 7D**, **8Q**), the *ARR17* gene (**Supplementary Data 7F**, **8S**), and the joint analysis of 14 loci used in poplar phylogenetic studies (**Figure 1**, **Supplementary Data 6A**, **8A**), these two samples were part of the *P.* × *rasumovskoe* cluster. A more detailed analysis of the morphological data on these accessions raised the question of the accuracy of their identification – it is very likely that they morphologically also fit the description of *P.* × *rasumovskoe* subjected to cardinal pruning. Accession 205, morphologically identified as *P.* × *sibirica*, also fell into the *P.* × *rasumovskoe* group rather than into the *P.* × *sibirica* group when analyzed on the basis of different sequenced regions. We can assume an error in the identification of this accession on the basis of morphological characteristics due to cardinal pruning as well.

In the dendrogram based on all sequenced loci (**Figure 5**, **Supplementary Data 7G**, **8T**), the subcluster of male *P.* × *petrovskoe* genotypes contained 18 samples with unknown or doubtful species affiliation: 23, 25, 30, 32, 37, 39, 40, 43, 149, 171, 180, 192, 221, 222 (29), 224, 225, 226, 229. The same pattern was observed when analyzed separately for the SDR (**Figure 4**, **Supplementary Data 7E**, **8R**) and the 14 loci used in poplar phylogenetic studies (**Figure 1**, **Supplementary Data 6A**, **8A**). It is likely that these 18 accessions belong to *P.* × *petrovskoe*, and the male genotypes of *P.* × *petrovskoe* are genetically close (**Supplementary Data 5**) and may be descendants of the same Moscow clone.

In the dendrogram based on all sequenced loci (**Figure 5**, **Supplementary Data 7G**, **8T**), the subcluster of female genotypes of *P.* × *petrovskoe* contained 27 samples with unknown or doubtful species affiliation: 33, 34, 157, 160, 163, 174, 175, 176, 178, 232, 233, 234, 235, 239, 312, 313, 314, 315, 316, 317, 318, 319, 320, 321, 325, 339 (4) K, 340 (5) K. In addition, samples 212 and 213 (identified as *P.* × *rasumovskoe*), 138 (*P. deltoides* × (*P. laurifolia* × *P. suaveolens*)), and 170 (*P.* × *sibirica*) fell into the same subcluster. In the analysis based on the 14 loci used in phylogenetic studies of poplars (**Figure 1**, **Supplementary Data 6A**, **8A**), the subcluster of *P.* × *petrovskoe* female samples included all the same accessions. When analyzed only by the SDR loci (**Figure 4**, **Supplementary Data 7E**, **8R**), all samples except 316 and 317 were also included in the *P.* × *petrovskoe* female genotype cluster (316 was in the group with *P. laurifolia*, and 317 was in the group with *P. suaveolens*). Most likely, 25 of 27 uncertain accessions belong to *P.* × *petrovskoe*. Samples 212 and 213, identified as *P.* × *rasumovskoe* (the remaining accessions of this hybrid formed a dense subcluster distant from *P.* × *petrovskoe*), and sample 170, identified as *P.* × *sibirica* (most accessions of this hybrid also formed a detached subcluster), also belong to *P.* × *petrovskoe*. For samples 138, 316, and 317, further analysis is required for final conclusions. It should be noted that in the cluster of most female accessions of *P.* × *petrovskoe*, there were more difficulties in species identification by morphological features than in the cluster of male *P.* × *petrovskoe* genotypes – in the first cluster, a significant number of samples were represented by genotypes with uncertain or doubtful species affiliation, including doubtful *P.* × *sibirica* accessions. At the same time, accessions of the subcluster of female *P.* × *petrovskoe* genotypes were genetically very close (**Supplementary Data 5**), and there is a high probability that they are the descendants of the same clone distributed in Moscow.

In the cluster of the majority of female accessions of *P.* × *sibirica* when analyzed by all sequenced loci (**Figure 5**, **Supplementary Data 7G**, **8T**), there were also 33 samples with unknown or doubtful species affiliation: 21, 28, 44, 45, 46, 48, 50, 130, 162, 173, 203, 204, 231, 236, 237, 238, 240, 264, 308, 309, 310, 311, 323 (1), 326, 327, 328, 329, 330, 331, 332, 333, 334, 335. In addition, this cluster included accessions of *P. deltoides* × *P.* × *moscoviensis* (363 (25) and 364 (26)), *P.* × *petrovskoe* (194, 197, 198), and *P. simonii* var. *fastigiata* (372). When analyzed only by the SDR (**Figure 4**, **Supplementary Data 7E**, **8R**), the same samples were also in the cluster of female *P.* × *sibirica* accessions. When analyzed on the basis of the 14 loci used in poplar phylogenetic studies (**Figure 1**, **Supplementary Data 6A**, **8A**), 30 of the 33 poplars with uncertain species affiliation (except for 21, 328, 329) were also in the *P.* × *sibirica* cluster. It is likely that these 30 accessions belong to *P.* × *sibirica*. In addition, errors in the identification of samples 194, 197, 198 as *P.* × *petrovskoe* (since most female accessions of this hybrid formed a separate cluster, and after cardinal pruning this hybrid could be confused with *P.* × *sibirica*) and error at the stage of sample preparation for accession 372, listed as *P. simonii* var. *fastigiata* (this species is difficult to confuse with *P.* × *sibirica* by its morphological features). For accessions of *P. deltoides* × *P.* × *moscoviensis* (363 (25) and 364 (26)), the question of the accuracy of identification remains open. For female accessions of *P.* × *sibirica*, we observed a different pattern from *P.* × *rasumovskoe* and *P.* × *petrovskoe*: for this group, the samples were not as genetically close to each other as in *P.* × *rasumovskoe* and *P.* × *petrovskoe* cases, and they probably originated not from one but from several original clones.

It is worth noting that in the analysis based on all loci we sequenced (**Figure 5**, **Supplementary Data 7G**, **8T**), a subcluster close to the main subcluster of *P.* × *sibirica* female samples was formed by *P.* × *irtyschensis* (Pgib35 and Pgib38), *P.* × *sibirica* × *P. nigra* (k-78-2016), *P. nigra* (KPN4 and k-2-2-2016), and *P.* × *sibirica* (k-1-2016 and k-13-2016). When analyzing only the SDR loci (**Figure 4**, **Supplementary Data 7E**, **8R**), *P. nigra* (KPN4 and k-2-2-2016) formed a separate subcluster with *P.* × *sibirica* (k-1-2016 and k-13-2016) accessions in a large subcluster of *P.* × *sibirica*, but *P.* × *irtyschensis* (Pgib35 and Pgib38) and *P.* × *sibirica* × *P. nigra* (k-78-2016) samples were in a different subcluster and were closer to *P. afghanica* than to the main *P.* × *sibirica* subcluster. When the 14 loci used in poplar phylogenetic studies were analyzed (**Figure 1**, **Supplementary Data 6A**, **8A**), the picture for the group of female *P.* × *sibirica* accessions was slightly different from those obtained on the basis of the SDR or all sequenced loci. *P.* × *sibirica* (k-1-2016 and k-13-2016) and *P. nigra* (KPN4 and k-2-2-2016) samples moved to a large subcluster of *P.* × *sibirica* × *P. nigra* and *P.* × *irtyschensis* samples. *P.* × *sibirica* samples k-1-2016 and k-13-2016 were collected in the Novokuznetsk district of Kemerovo Region, in contrast to the rest of the *P.* × *sibirica* samples collected in Moscow. This may explain their clustering with *P.* × *sibirica* × *P. nigra* and *P.* × *irtyschensis*, also collected in the Novokuznetsk district. Thus, the relationship between the geographical origin of the samples and genotype was revealed for these accessions.

In our sample set we had two accessions from Uzbekistan (ANG11 and CHim11), which were listed in the herbarium as *P. nigra*, but could belong to *P. afghanica*, because they were collected outside the area of *P. nigra*, and these two species are not always distinguishable by a single shoot on a herbarium leaf. ANG11 was in the same cluster as the three *P. afghanica* accessions we studied, and probably belongs to this species rather than to *P. nigra*. CHim11 clustered with the rest of the *P. nigra* samples and indeed belongs to this species.

In addition, a clear error, probably associated with sample preparation, is related to sample 265 (possibly confused with sample 372), identified as *P.* × *rasumovskoe*, which was in the subcluster of *P. simonii* accessions in the analysis for both all loci studied and individual loci. *P.* × *rasumovskoe* and *P. simonii* are morphologically very different, so an error in the morphological determination of species affiliation is unlikely.

Thus, we have shown a high informativity of the use of the sex-determining region to study the relationships between representatives of sections *Aigeiros* and *Tacamahaca*, especially interspecific hybrids, which are difficult for systematics. Joint analysis of the targeted deep sequencing data for the SDR and *ARR17* gene, as well as for the used in molecular phylogeny sequences of NTS 5S rDNA, ITS, *DSH 2*, *DSH 5*, *DSH 8*, *DSH 12*, *DSH 29*, *6*, *15*, *16*, *X18*, *trnG-psbK-psbI*, *rps2-rpoC2*, *rpoC2-rpoC1* was informative in determining the taxonomic affiliation of poplar accessions.

## 4 Discussion

To study the genetic relationship of poplar species and hybrids from sections *Aigeiros* and *Tacamahaca*, we chose an approach based on targeted deep sequencing of amplicons. This method drastically reduces the cost of analysis per sample compared to whole genome sequencing, has incomparable accuracy in the study of heterozygous genotypes and multicopy sequences, as well as less laboriousness in the analysis of large sample sets for a significant number of loci compared to Sanger sequencing (McCormack et al., 2013; Cruaud et al., 2017; Borkhert et al., 2018; Melnikova et al., 2019; Dmitriev et al., 2020; Novakovskiy et al., 2020; Povkhova et al., 2022). However, effective application of targeted deep sequencing requires the selection of the most informative loci.

Compared to the previous studies on *Populus* species based on the analysis of multicopy and single-copy sequences of nuclear and chloroplast genomes (Schmidt et al., 1994; Leskinen and Alström-Rapaport, 1999; Page, 2000; Huson and Scornavacca, 2011; Schroeder et al., 2012; Huang et al., 2014; Wang et al., 2014; Alexandrov and Karlov, 2018; Borkhert et al., 2018; Zhou et al., 2018; Wang et al., 2019), we sequenced regions of the SDR and the *ARR17* gene, which is the key gene in sex determination in the genus *Populus* (Muller et al., 2020). The SDR allowed us to reveal the origin of the examined genotypes in the male lineage and significantly complemented the results obtained in the analysis of the DNA sequences traditionally used in poplar studies.

We studied interspecific hybrids, including those created by breeders, actively used in landscaping, as well as hybrids of natural origin and “pure” species from sections *Aigeiros* and *Tacamahaca*, which have an XY sex-determination system and interbreed easily. In poplars of these sections, the SDR is localized on the peritelomeric region of chromosome 19 and includes male-specific region containing partial repeats of the *ARR17* gene, and a region that is present in plants of both sexes but differs significantly in DNA polymorphisms between males and females (Muller et al., 2020; Xue et al., 2020; Zhou et al., 2020; Melnikova et al., 2021; Pushkova et al., 2021), which is probably associated with the suppression of recombination. In this regard, the SDR could be promising for use in phylogenetic studies of species and hybrids of sections *Aigeiros* and *Tacamahaca*. Previously, based on whole-genome sequencing data, we showed the informativity of the SDR polymorphisms for determining putative parental forms of poplar hybrids (Melnikova et al., 2021). However, this approach requires significant costs for the analysis of one sample, so the analysis of large sample sets is complicated. Therefore, in this work, we designed primers for the amplification of the SDR loci of poplars of sections *Aigeiros* and *Tacamahaca* containing sex-specific polymorphisms (**Supplementary Data 3**) for subsequent targeted deep sequencing.

Analysis of the SDR sequences made it possible to divide the studied samples of sections *Aigeiros* and *Tacamahaca* into male and female, and within groups of the same sex, the samples were predominantly clustered according to belonging to a particular hybrid or species (**Figure 4**, **Supplementary Data 7E**, **8R**). In addition to the SDR, we also sequenced DNA sequences traditionally used in poplar phylogenetic studies, namely NTS 5S rDNA, ITS, *DSH 2*, *DSH 5*, *DSH 8*, *DSH 29*, *6*, *15*, *16*, *X18*, *trnG-psbK-psbI*, *rps2-rpoC2*, *rpoC2-rpoC1* (Schmidt et al., 1994; Schroeder et al., 2012; Huang et al., 2014; Wang et al., 2014; Zhou et al., 2018; Wang et al., 2019). Clustering based on multicopy (NTS 5S rDNA and ITS) and single-copy (*DSH 2*, *DSH 8*, *DSH 29*, *6*, *15*, *16*, *X18*) sequences of the nuclear genome and regions of the chloroplast genome (*trnG-psbK-psbI* and *rps2-rpoC2*) made it possible to separate a number of samples into groups according to belonging to a particular hybrid (often) or species (less often), however, none of the sequences allowed us to obtain a clear picture corresponding to the understanding of the similarity of the studied accessions based on morphological data. The most complete information on the phylogenetic relationships of the studied poplar accessions was achieved through the joint analysis of data for multicopy and single-copy sequences of nuclear DNA, loci of chloroplast DNA, and the SDR – clustering based on all obtained data of targeted deep sequencing most closely corresponded to the ideas about systematics of poplars based on morphological characteristics (**Figure 5**, **Supplementary Data 7G**, **8T**). Since the results of genetic analysis corresponded to the known facts, we can assume that they can be used to test hypotheses about the origin of poplars, as well as to determine the species identity of accessions whose identification on the basis of morphological features was complicated.

The analysis of sequencing data for the SDR and chloroplast genome loci made it possible to determine the origin of *P.* × *petrovskoe* [*P. laurifolia* (female tree) × *P.* × *canadensis* (male tree)] and *P.* × *rasumovskoe* (*P. nigra* (female tree) × *P. suaveolens* (male tree). The original description of *P.* × *rasumovskoe* given by M. Wolkenstein is as follows: “A hybrid between *P. nigra*, fertilised with the pollen of *P. suaveolens*” (Wolkenstein, 1882). It agrees with the results obtained by us on the basis of genetic analysis. However, for *P.* × *petrovskoe*, the original description by M. Wolkenstein is as follows: “A hybrid between *P. canadensis* fertilised by the pollen of *P. suaveolens*” (Wolkenstein, 1882). The indication of *P. suaveolens* as the parental species for *P.* × *petrovskoe* is a probable mistake by M. Wolkenstein. The Russian poplar species of the section *Tacamahaca*, of which there are two, were primarily involved in hybridization in Russia, and the “balsamic” component in *P.* × *petrovskoe* is probably represented by *P. laurifolia*, which is also proved morphologically by the ribbing of shoot axes (Mayorov et al., 2020). The sequencing data also confirm the error in the original description of *P.* × *petrovskoe*. The first parental species, *P.* × *canadensis*, was indicated correctly by Wolkenstein, although it went to the hybrid in the male lineage.

We analyzed the relationships of the studied poplar species and hybrids of sections *Aigeiros* and *Tacamahaca* based on molecular genetic (obtained results of targeted deep sequencing of NTS 5S rDNA, ITS, *DSH 2*, *DSH 5*, *DSH 8*, *DSH 29*, *6*, *15*, *16*, *X18*, *trnG-psbK-psbI*, *rps2-rpoC2*, *rpoC2-rpoC1*, *ARR17*, and *SDR* – **Figure 5**), morphological, and ecological-geographical data. Below is our interpretation of the results obtained for particular poplar species and hybrids.

It is known that not universally recognized *P. koreana* and *P. maximowiczii* species are morphologically very close to *P. suaveolens* or even coincide with it (Skvortsov and Belyanina, 2006). In **Figure 5** and dendrograms based on individual loci (**Supplementary Data 6**, **7**), *P. koreana* and *P. maximowiczii* accessions always appear together with *P. suaveolens* ones. Thus, the obtained results indicate not only genetic proximity but also the identity of *P. suaveolens*, *P. koreana*, and *P. maximowiczii*, confirming the controversial assumption of Skvortsov and Belyanina (Skvortsov and Belyanina, 2006), which is still not recognized by most botanists, that *P. koreana* and *P. maximowiczii* should not be distinguished as species, but should be assigned to *P. suaveolens*. If these taxa are understood separately, we can speak only about geographical races, which are strictly confined to certain regions (*P. koreana* to Korea, *P. maximowiczii* to the Far East, etc.). It is unfounded to consider, as it is usually done, that all three species grow simultaneously, for example, in the Far East south of Amur. In this case, we can speak only about the external deviation to the appearance peculiar to a certain race.

All *P.* × *canadensis* accessions were arranged compactly in **Figure 5**, with only *P. deltoides* and its hybrid with *P.* × *moscoviensis* falling into their subcluster, and this is natural, since *P.* × *canadensis*, according to the well-established notions, is a hybrid of *P. deltoides* and *P. nigra*. The distinction between *P.* × *canadensis* and *P. deltoides* is sometimes difficult; in the past, they were generally mixed (Skvortsov, 2010), and even errors in identification are possible. In the neighboring subclusters, exclusively *P. nigra* and *P. pyramidalis* appeared, and in the next ones by distance, almost exclusively *P.* × *petrovskoe* was observed. *P.* × *petrovskoe*, according to our recent assumption (Mayorov et al., 2020), is a hybrid of *P.* × *canadensis* and *P. laurifolia*, and the obtained molecular genetic results confirm this hypothesis. This result is not trivial at all, because previously the putative parental species of *P.* × *petrovskoe* were indicated as *P. deltoides* s.l. × *P. suaveolens* (Regel, 1889; Wolkenstein, 1882); *P. deltoides* × *P. jackii* (Dippel, 1892); *P*. × *canadensis* [or also *P. deltoides*?] × *P. suaveolens* (Bogdanov, 1965); *P. deltoides* × *P. laurifolia* (Koltzenburg, 1999; Tsvelev, 2001). Only Skvortsov (Skvortsov, 2010) indicated the same *P.* × *canadensis* and *P. laurifolia*, but in relation to *P.* × *berolinensis* K.Koch, for which this researcher mistakenly took *P.* × *petrovskoe*.

Most *P. nigra* accessions in **Figure 5** were compactly arranged in cluster III together with *P. pyramidalis*. This group is adjacent to *P.* × *canadensis*, *P. deltoides*, and *P.* × *petrovskoe*. Several more *P. nigra* accessions were in other clusters, and for 5 of them, *P.* × *irtyschensis* accessions were the closest neighbors. Another close neighbor of *P. nigra* is *P. laurifolia*, which, according to our conceptions (Nasimovich et al., 2019), is a hybridogenic species that originated with the involvement of *P. nigra*. These results are quite natural, they support theoretical insights, but even more so, they support the ability of the analysis based on the targeted deep sequencing of the SDR and other nuclear and chloroplast loci used in phylogenetic studies to examine poplars. Importantly, samples of *P. nigra* and closely related species (primarily *P.* × *irtyschensis*) formed joint compact groups simultaneously in three different clusters (II, III, V). This suggests that the distribution of accessions, in addition to species affiliation, is influenced by the general polymorphism of *P. nigra* [also shown by Wang et al. (Wang et al., 2022)] and closely related species, and this must be taken into account when working with poplars. It is also important that accessions of *P. nigra* and closely related species did not fall into cluster I, formed primarily by *P. suaveolens* (including *P. koreana* and *P. maximowiczii*). This confirms the idea expressed earlier (Nasimovich and Vasilyeva, 2019), that *P. nigra* and *P. suaveolens* are geographical, biotopic, and morphological antipodes, occupying as if opposite “poles” in the unified system of Eurasian poplars. It turns out that they are also genetic “poles” of this system.

All but one *P. laurifolia* accessions in **Figure 5** formed a compact group of subclusters in cluster IV, isolated from *P. nigra* and closely related species, and isolated from *P. suaveolens* and closely related species (cluster I). This probably implies the species independence of *P. laurifolia*, although we have argued that *P. laurifolia* emerged as a hybridogenic species at the intersection of gene flow from *P. nigra* and *P. suaveolens* (Nasimovich et al., 2019). Based on the molecular genetic analysis, *P. laurifolia* is slightly closer to *P. nigra* than to *P. suaveolens*. *P. laurifolia* is one of the “balsam” poplars, but partially shares a common habitat with *P. nigra*, which turns out to be more important. In the same or close subclusters, together with *P. laurifolia*, there were its putative hybrids: *P.* × *irtyschensis* (*P. laurifolia* × *P. nigra* – 1 sample) and *P.* × *wobstii* (*P. laurifolia* × *P. longifolia* – 2 samples), which confirms their hybrid nature and the putative composition of parental species. For *P.* × *wobstii*, this result is not quite trivial, because previously the putative parental species of *P.* × *wobstii* were indicated as *P. suaveolens* (Regel, 1889); *P. balsamifera* (Dippel, 1892); *P. suaveolens* and *P. jackii* (Sokolov et al., 1951); *P. simonii* and *P. suaveolens* (Karhu and Hamet-Ahti, 1992); *P. laurifolia* and *P. tristis* (Rehder, 1949; Koltzenburg, 1999); *P. laurifolia* and *P. tristis* (or *P. longifolia*) (Ascherson and Graebner, 1908-1913; Tsvelev, 2001).

Most *P. longifolia* accessions in **Figure 5** were in cluster I together with *P. suaveolens* and closely related species, but they were absent in clusters III (together with *P. nigra*) and IV (together with *P. laurifolia*). This indirectly confirms our hypothesis on the origin of *P. longifolia* from *P. suaveolens* (Mayorov et al., 2012), although for complete certainty it is necessary to investigate in the same way the similarity with the American species *P. balsamifera* and *P. trichocarpa*, which are also considered as ancestors of *P. longifolia* (Tsinovskis, 1977; Skvortsov, 2008).

*P. suaveolens* and closely related species constitute the vast majority in cluster I (**Figure 5**) and, in addition, are present in cluster II (3 accessions) together with *P.* × *rasumovskoe*, while in clusters III and IV, where *P. nigra* and *P. laurifolia* dominate among “pure” species, they are absent. This means a molecular genetic opposition with respect to other (more western) “pure” species, as already mentioned above. *P. longifolia*, *P. talassica*, *P. afghanica*, and the hybrid *P.* × *rasumovskoe* (*P. nigra* × *P. suaveolens*), which form clusters I and II, are also close to *P. suaveolens* (together with *P. koreana* and *P. maximowiczii*). *P. talassica* has even been combined with *P. suaveolens* into one species in the past; *P. afghanica* is geographically close to *P. suaveolens*; *P. longifolia* may have separated from *P. suaveolens*; and the situation with *P.* × *rasumovskoe* is even clearer (see above). In general, the position of *P. suaveolens* on the dendrogram is completely logical and shows great opportunities for the use of targeted deep sequencing of chloroplast and nuclear sequences, including the SDR, in the study of poplars. Analysis of sequencing data revealed the genetic proximity of *P. talassica* and *P. afghanica*. These results support the hypothesis of hybridogenic origin of *P. afghanica* – this species is close to *P. nigra* by the traits of leaves and shoots, but there are also traits characteristic of poplars of the section *Tacamahaca*, probably derived from *P. talassica* (**Supplementary Data 2**). It can also be seen that the geographical proximity of species and, therefore, the possibility of hybridization between them is sometimes more important in terms of genetics than the sectional position of species: *P. suaveolens* and *P. afghanica* belong to different sections, but are geographically close and therefore alike.

Accessions of the complex hybrid *P.* × *petrovskoe*, together with its putative parental species, *P. deltoides* and *P. nigra*, form the core of neighboring clusters III and IV (**Figure 5**), which proves their kinship. Another putative parental species of *P.* × *petrovskoe*, *P. laurifolia*, is also a part of cluster IV. It is also important that *P.* × *petrovskoe* does not overlap with *P. suaveolens* (clusters I and II) in **Figure 5**, and it is the only mass hybrid of Moscow landscaping whose parental species, according to our knowledge (Mayorov et al., 2020), does not include *P. suaveolens*. Thus, the molecular genetic analysis fully confirmed our ideas about *P.* × *petrovskoe*.

**Figure 6:**
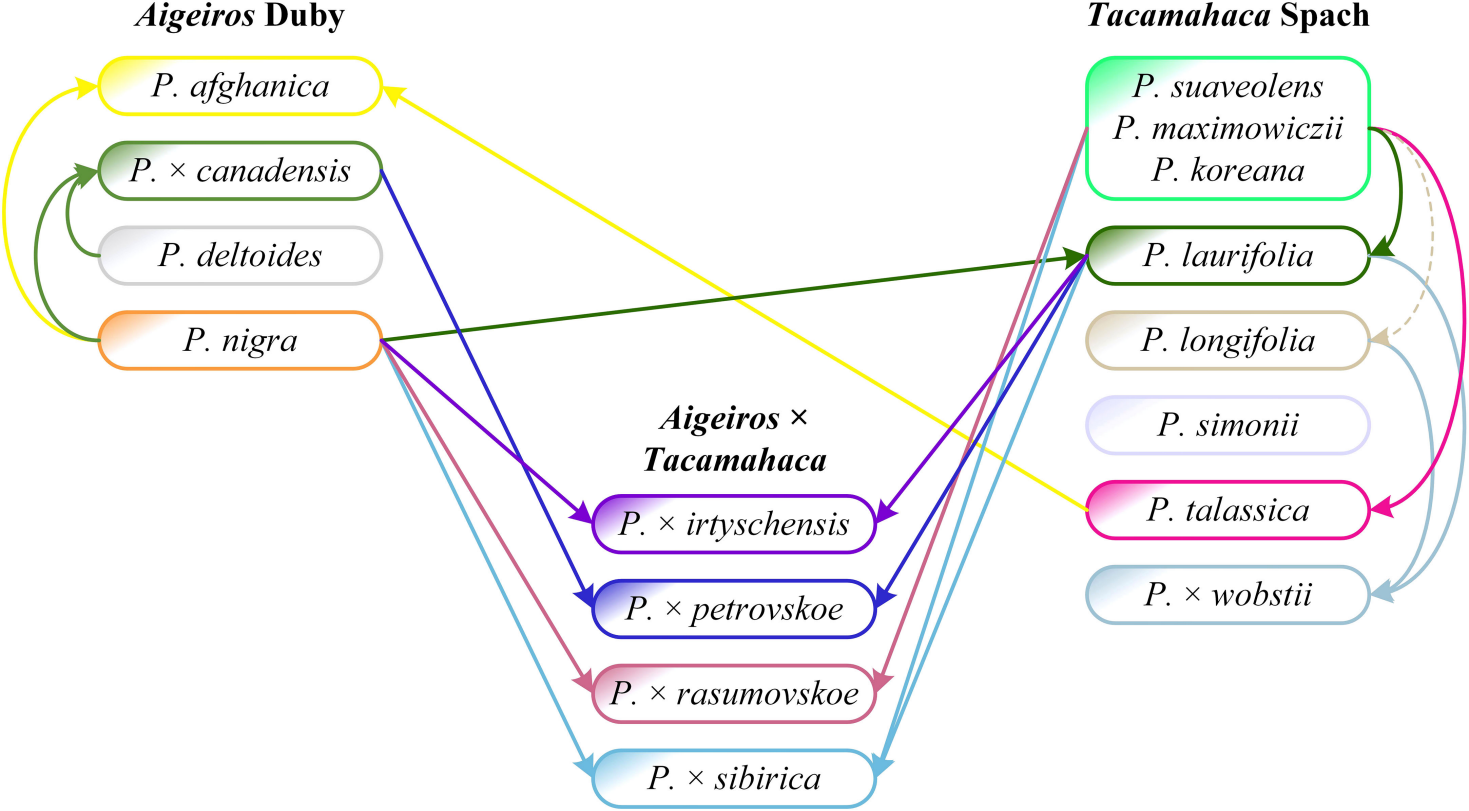
Scheme of relationships between species and hybrids of sections *Aigeiros* and *Tacamahaca*. Arrows connect a probable ancestor and a descendant, are directed from an ancestor to descendant, and have the same color as a probable descendant. A dotted arrow indicates that there are doubts about the relationship.

Based on the results of targeted deep sequencing and on the analysis of morphological and ecological-geographical data (see **Supplementary Data 2**), we present a scheme of relationships between species and hybrids of sections *Aigeiros* and *Tacamahaca* (**Figure 6**). It should be noted that relationships between taxa from different sections are traced. *P. afghanica* (*Aigeiros*) is mountain poplar and morphologically close to *P. nigra* (*Aigeiros*), but also has some features similar with “balsam” poplars. We supposed that *P. afghanica* can be a species of hybrid origin that arose at the intersection of gene flow from *P. nigra* and *P. talassica* (*Tacamahaca*). The obtained genetic data support this hypothesis. *P. laurifolia* (*Tacamahaca*), which is a fairly typical representative of “balsam” poplars and has all diagnostic features of this section, is genetically close to *P. nigra* (*Aigeiros*), but distant from another “balsam” poplar, *P. suaveolens* (*Tacamahaca*). In addition, a significant number of poplar hybrids, including *P.* × *petrovskoe*, *P.* × *rasumovskoe*, *P.* × *sibirica*, and *P.* × *irtyschensis*, come from crosses of representatives of different sections.

In the group of “black” poplars (*Aigeiros*), there are similarities of *P.* × *canadensis* with *P. nigra* and *P. deltoides*. The genetic data are consistent with the known information that *P. nigra* and *P. deltoides* are the parental species of *P.* × *canadensis*. In the group of “balsam” poplars (*Tacamahaca*), it is inexpedient to distinguish *P. suaveolens*, *P. koreana*, and *P. maximowiczii* as separate species – they are a single species according to both morphological (Skvortsov and Belyanina, 2006) and genetic data. *P. suaveolens* is a prominent representative of “balsam” poplars growing in mountain conditions. It is probably the parental species for *P. laurifolia*, *P. talassica*, and possibly *P. longifolia* based on the analysis of both morphological and genetic data. *P.* × *wobstii* probably comes from crosses between *P. laurifolia* and *P. longifolia*. According to genetic data, *P. simonii* is close to *P. suaveolens*, which can be explained by the ecological-geographical relationship of these species.

Considering the status of sections *Aigeiros* and *Tacamahaca*, it should be noted that their representatives freely interbreed in both cultivation and nature. Meanwhile, sections *Aigeiros* and *Tacamahaca* differ in a large set of traits (more details in Materials and Methods), but all these traits are adaptations to either plain (“black” poplars) or mountain conditions (“balsam” poplars) (Nasimovich et al., 2019). The traits of “black” poplars are most pronounced in *P. nigra* growing on the vast Russian plain, while the traits of “balsam” poplars are most expressed in *P. suaveolens* growing in the harsh mountain conditions of the Eastern Siberia (Nasimovich and Vasilyeva, 2019). Our study revealed significant genetic differences between *P. nigra* and *P. suaveolens*. Considering a number of other poplar species (e.g., *P. laurifolia* and *P. afghanica*), the genetic unity within sections is not revealed. Geographical proximity and the possibility of gene exchange turn out to be more important in terms of genetics than the sectional position. Therefore, sections *Aigeiros* and *Tacamahaca* are most likely ecological (plain and mountain poplars) and have no phylogenetic or genetic meaning. However, discarding these sections would make the taxonomy of poplars less convenient, as we discussed earlier (Nasimovich et al., 2019).

The performed genetic analysis also made it possible to establish belonging to the common Moscow hybrids, *P.* × *rasumovskoe*, *P.* × *petrovskoe*, and *P.* × *sibirica*, for a number of accessions with unknown or doubtful origins, as well as to find several errors in identification of accessions. To control fluff in Russian cities, poplars are often subjected to cardinal pruning leading to changes in the traits of leaves, shoots, and crowns, which makes it difficult to identify poplar species and hybrids on the basis of morphological characteristics, and for such accessions, genetic analysis can clarify the situation.

Our study allowed us to evaluate the polymorphism of the three hybrids most commonly used in Moscow landscaping: *P.* × *rasumovskoe*, *P.* × *petrovskoe*, and *P.* × *sibirica*. The analysis based on the data for all sequenced regions at once provided the clearest picture of the separation of samples of these hybrids into their respective groups, but even analyses by individual loci in most cases allowed us to separate the *P.* × *rasumovskoe*, *P.* × *petrovskoe*, and *P.* × *sibirica* groups (**Figures 1**-**5**, **Supplementary Data 6**, **7**).

*P.* × *rasumovskoe* was represented by male accessions that were genetically very close – the average genetic distance between accessions was 0.2 (**Supplementary Data 5**). *P.* × *rasumovskoe* is one of the spontaneous hybrids discovered before 1882 by R.I. Schroder at the Moscow Agricultural Academy (Wolkenstein, 1882). Probably, all accessions of *P.* × *rasumovskoe* studied by us are descendants of one clone, which was quite reasonably used in landscaping of Moscow – male plants do not form fluff and have an attractive weeping crown shape.

The studied samples of *P.* × *petrovskoe* were both male and female. Most male accessions were genetically close (the average genetic distance between the accessions was 0.2) and were probably the descendants of one clone. However, two other small groups of *P.* × *petrovskoe* male accessions were genetically different from the main group (the average genetic distance between them and the main group was 0.8) (**Supplementary Data 5**). The main group of female trees of *P.* × *petrovskoe* also probably came from one clone (the average genetic distance between the accessions was 0.2), and the three genetically different female accessions most likely belong not to *P.* × *petrovskoe* but to *P.* × *sibirica*. Thus, two clones of *P.* × *petrovskoe* were predominantly used in Moscow landscaping – male one and female one. While the use of the male clone is justified, the planting of female plants was a mistake that led to the appearance of poplar fluff in the city during the ripening of seeds (poplar fluff contains seeds).

For *P.* × *sibirica*, the picture was different from that obtained for *P.* × *rasumovskoe* and *P.* × *petrovskoe*. *P.* × *sibirica* was represented predominantly by female trees, and not as genetically similar as in the case of *P.* × *rasumovskoe* and *P.* × *petrovskoe*. It can be assumed that several predominantly female *P.* × *sibirica* clones were used in the landscaping of Moscow. Such a choice was unreasonable and led to a massive problem with poplar fluff.

It is worth noting that among Moscow citizens an opinion about the sex change of poplars is widespread, including due to the media. There is a belief that originally only male plants were planted, and then under the influence of adverse factors they have changed sex to a female, and this has led to the problem of poplar fluff, which is formed only on female plants. Our work shows that the cause of the mass appearance of fluff is errors in the choice of initial material for landscaping, and not at all the sex change of poplars. Only in the case of *P.* × *rasumovskoe*, descendants of a male clone were used. For *P.* × *petrovskoe*, both male and female descendants were planted. And for *P.* × *sibirica*, descendants of several female clones were mostly used in landscaping. We observed complete correspondence between the presence of female or male generative organs on *P.* × *rasumovskoe*, *P.* × *petrovskoe*, and *P.* × *sibirica* trees and the division of all samples of these hybrids into two sexes based on the sequences of the SDR. In this regard, the assumption of mass sex change in Moscow poplars is erroneous. The possibility of formation of male, female, and hermaphroditic generative organs on one tree and the change of their ratio during tree development was shown for hybrids of white poplar (*Populus alba* L.) and aspen (*Populus tremula* L.) – gray poplar (*Populus* × *canescens* (Aiton) Sm.) (Sabatti et al., 2020). This is due to the fact that *P. alba* has a ZW system of sex determination and *P. tremula* has an XY system of sex determination (Cronk and Muller, 2020; Muller et al., 2020). *P.* × *canescens*, as well as *P. alba* and *P. tremula*, occurs in Moscow an order of magnitude less frequently than intersectional hybrids of poplars of sections *Aigeiros* and *Tacamahaca* (Nasimovich et al., 2019; Mayorov et al., 2020); they can also be identified using genetic markers (Kim et al., 2021).

Thus, the use of the SDR loci considerably complements the sequences traditionally used for phylogenetic studies to clarify poplar systematics, makes it possible to divide accessions into male and female and determine the ancestral species in the male lineage. We shed light on a number of controversial hypotheses on the division of poplar accessions into species and on the origin of a number of species and hybrids. A detailed analysis of the obtained data on the SDR sequences and the regions of nuclear and chloroplast genomes traditionally used in phylogenetic studies of poplars with involvement of morphological data will make it possible to clarify the origin and systematics of other studied species of the genus *Populus* as well.

## Data availability statement

The datasets presented in this study can be found in online repositories. The names of the repository/repositories and accession number(s) can be found below: https://www.ncbi.nlm.nih.gov/, BioProject PRJNA943410.

## Author contributions

AD and NM conceived and designed the work. EB, EP, YN, MK, NV, RM, RN, LP, DZ, AT, ES, AS, and NB performed the experiments. EB, EP, YN, MK, RM, ED, AK, NB, GK, AD, and NM analyzed the data. EB, EP, YN, DZ, GK, AD, and NM wrote the manuscript. All authors contributed to the article and approved the submitted version.
