# Supplementary material for: Sex-determining region complements traditionally used in phylogenetic studies nuclear and chloroplast sequences in investigation of Aigeiros Duby and Tacamahaca Spach poplars (genus Populus L., Salicaceae)
Source: Front Plant Sci. 2023 Oct 4;14:1204899. doi: 10.3389/fpls.2023.1204899 (PMC10582643; doi:10.3389/fpls.2023.1204899)
Supplement: Supplementary file 2 [file DataSheet_2.docx]

**Supplementary Data 2. Description of poplar accessions of sections *Aigeiros* and *Tacamahaca* used in the study.**

Initially, we focused on studying the sex-determining region (SDR) in the genus *Populus*, poplars from sections *Aigeiros* Duby and *Tacamahaca* Spach of Moscow landscaping. The sex of the examined trees was considered, but the species affiliation did not matter.

Two years later, the goals became bigger, and this required the identification of species and hybrids from which leaves were taken. The relevant experts were involved in the work, but, not for all previously examined trees, taxonomic status could be determined despite knowledge of their coordinates. Failures were associated with the deaths of several trees, strong trimming, the presence of a highly raised crown, and other causes. Therefore, 57 trees are presented in the table with a sex indication but without species identification. All of them represent the urban landscaping of Moscow. Our further genetic studies have shown that, as a rule, these samples belong to the three main intersectional hybrids of Moscow landscaping – *P*. × *sibirica*, *P*. × *petrovskoe*, and *P*. × *rasumovskoe*.

At the second step, the material used was not only from Moscow (249 samples, including those collected earlier) and the Moscow Region (11 samples), but also from the other Russian regions (96 samples): Orenburg Region (4 samples), Novosibirsk Region (Novosibirsk) (8 samples), Altai Republic (11 samples), Kemerovo Region (19 samples), Republic of Khakassia (2 samples), Krasnoyarsk Territory (4 samples), Transbaikal Territory (10 samples), Republic of Sakha (Yakutia) (3 samples), Jewish Autonomous Region (2 samples), Khabarovsk Territory (20 samples), Primorsky Territory (11 samples), Sakhalin Region (1 sample), and Magadan Region (1 sample). In addition, samples from Central Asia (16) were studied: 7 from Kazakhstan, 5 from Kyrgyzstan, 4 from Uzbekistan. Besides, 3 samples from Mongolia and 4 samples from Italy were used. We imply both our collected samples (Moscow, Moscow Region, Novosibirsk, Novokuznetsk, Krasnoyarsk Territory, Western Sayan, Transbaikalia) and herbarium materials of the other collectors. In total, 379 trees belonging to 10 “pure” species and 12 hybrids (simple, complex, backcross) were studied.

We basically had no difficulties with the identification of “pure” species, although we have to indicate the description of some taxa, as they can be understood in a broad or narrow sense. Unfortunately, taking “pure” species from a city landscaping is incorrect, which means that collecting is associated with long journeys and, therefore, the corresponding material for several species is relatively scarce.

The identification of poplar hybrids is almost always complicated. Difficulties are associated with both the similarity of some hybrid taxa and their undeveloped systematics. The fact is that some hybrid taxa at the end of the XIX century and at the beginning of the XX century were described both in Russia and Western Europe incorrectly – unacceptably briefly, without the illustration of leaves, and sometimes without a typical herbarium specimen. As a result, in some cases, we do not reliably know what phenotype these or other binary names belong to. The presence of a typical specimen also does not always solve these problems, as hybrid poplars cannot always be determined by one branch without knowledge of the tree age, crown shape, and the type of reproduction (forms or does not form root suckers). Some hybrid taxa are understood differently in Russia and Western Europe, i.e., the same binary names belong to hybrids with different parental species. In cases when parental species are only approximately determined, the situation becomes completely confusing. Thus, for example, in Russia from the middle of the XX century, Berlin poplar (*P.* × *berolinensis*) is absolutely erroneously regarded as Petrovsky poplar (*P.* × *petrovskoe*) (Mayorov et al., 2020), and balsam poplar from North America (*P.* *balsamifera*) as Siberian poplar (*P.* × *sibirica*), which is a hybrid of three Russian poplar species (Mayorov et al., 2012; Mayorov et al., 2020). These errors were transferred to the descriptions of hybrid cultivars created by leading Russian breeders: their true parental species do not correspond to those indicated in the descriptions. Besides, in Russia, the descriptions were very often made only in Russian (for example, *P.* × *nevensis* Bogdanov, nom. inval.), and such taxa and their descriptions are not accepted outside Russia, although the corresponding hybrids (hybrid cultivars) actually exist. Working with hybrid poplars, we essentially find ourselves in the pre-Linnaean period of botany, when the name per se does not mean anything and we must give at least a brief description of each taxon. This is exactly what we do in this article, indicating at least the main diagnostic features, and sometimes giving a full description.

**Section *Aigeiros* Duby [“Black” poplars]**

The section combines mainly plain poplars, and all diagnostic features of the section (long glabrous laterally flattened petioles without groove, rhombic-like or deltate leaf blades, etc.) can be explained by the growth of trees in the valleys of lowland rivers (Nasimovich et al., 2019). Some species in this section grow in the valleys of mountain rivers, but in this case, they have some features of “balsam” poplars.

***Populus afghanica*** C.K.Schneid. (*P. usbekistanica* Kom.) [Afghan poplar, Uzbek poplar, Ili poplar, Tajik poplar] – three samples from Central Asia were studied: 2 from different points of Uzbekistan, 1 from Kazakhstan. There are two more samples from Uzbekistan, which were originally (in herbarium) listed as *P. nigra*, but may belong to *P. afghanica* (collected outside the area of *P. nigra*; these two species are not always distinguishable by one shoot in herbarium). The native species, collected in natural habitats. Samples collected by V.B. Kuvaev and A.K. Skvortsov are stored in the Herbarium of the Tsitsin Main Botanical Garden, Russian Academy of Sciences (international acronym – MHA). Mountain poplar. It is morphologically close to *P. nigra* (rhombic-like or deltate leaf blades, long laterally flattened petioles, yellowish or light gray axes of 1-2-year-old shoots), which is why it is usually considered to be one of the “black” poplars (section *Aigeiros*). Nevertheless, it has certain features bringing it closer to “balsam” poplars (the edges of the leaf blades are very rounded, which is why the leaves sometimes turn out to be almost round or wide-oval; the petioles are slightly pubescent, they sometimes have a slightly intended adaxial groove). Probably, it can be considered a species of hybrid origin that arose at the intersection of gene flows from *P. nigra* and *P. talassica*.

***Populus* × *canadensis*** Moench (*P. deltoides* × *P. nigra*) [Canadian poplar] – 3 samples from Moscow landscaping, from three different streets. We collected the samples in 2019. A set of cultivars of hybrid origin. Currently, it is unambiguously considered a hybrid of American *P. deltoides* s.l. and Eurasian *P. nigra*. In the XIX and early XX centuries, it was often mixed with “pure” *P. deltoides*, and this should be taken into account when working with old literature data (Skvortsov, 2010), but we consider these taxa separately, although some forms of *P.* × *canadensis* and some forms and species (when understood separately) of *P. deltoides* are very similar. *P.* × *canadensis* was introduced to Russia from Western Europe (Kotelova and Stelmakhovich, 1963; Recommendations, 1976), where its numerous varieties are known (Koltzenburg, 1999). We do not know if Moscow trees belong to specific forms, but the following cultivars are most likely for Moscow: *P.* × *canadensis* var. *serotina* (Hartig) Rehder (*P. nigra* var. *nigra* × *P. deltoides* var. *monilifera* (Aiton) A.Henry); *P.* × *canadensis* var. *marilandica* (Bosc Ex Poir.) Rehder (*P. nigra* var. *nigra* × *P.* × *canadensis* var. *serotina*) [clone #239 from the GDR or clones from Czechoslovakia]; *P.* × *canadensis* var. *regenerata* Rehder; *P.* × *canadensis* var. *robusta* (C.K.Schneid.) Hyl. (? *P. angulata* Aiton × *P. nigra* var. *plantiensis* C.K.Schneid.); In Russia, based on *P.* × *canadensis* var. *robusta*, *P.* × *rubrinervis* Hort., nov. inval. was created (Kotelova and Stelmakhovich, 1963; Recommendations, 1976). Features of Moscow accessions of *P.* × *canadensis* are usually intermediate between those of *P. deltoides* and *P. nigra*: leaf blades are smaller than those of *P. deltoides* and larger than those of *P. nigra*; at the petiole near the base of the leaf blade, there is a pair of glands, but only in a half of the tree leaves; there are cilia on the leaf margin, but they are rare, located only at the apex of the leaf and not on every leaf; the teeth on the margin of the leaf blade are of intermediate size. *P. × canadensis* leaves become yellow and fall off later than for most poplars of Moscow, and this feature is brought about by *P. deltoides*. Powerful trees have on average 1.5-2-fold larger trunk widths than other poplars of Moscow of the same age (although the width is inferior to the oldest accession by *P.* × *petrovskoe* near the main building of the Moscow Timiryazev Agricultural Academy, MTAA). The crown is thick, not pyramidal. Another intermediate feature can be noted, which is usually ignored in literature: the base of the leaf blade has irregular shape for some or many leaves – attenuate near the petiole and almost truncate or cuneate at a distance from the petiole (authors’ observation). Such a base is obtained by hybridization of poplars with a truncate base (*P. deltoides*) and a cuneate (*P. nigra*). Nevertheless, not all *P.* × *canadensis* have an irregular base of leaf blades; sometimes leaves are large but have no glands and cilia; or leaves are small but deltate and with glands (authors’ observation). In all of these cases, it is difficult to distinguish *P.* × *canadensis* from *P. deltoides* s.l. Perhaps, this is because some forms (species? geographic races?) of *P. deltoides* s.l. does not have very large leaves (for example*, P. deltoides* var. *monilifera* (Aiton) A.Henry) or lack glands (for example, *P. wislizeni* (S.Watson) Sarg.) (Rehder, 1949). Generally, in Moscow, *P.* × *canadensis* is variable and is likely represented by several cultivars of different origins; however, we have never seen here some well-recognized cultivars that are specific for Western Europe and Russia. By number, *P*. × *canadensis* is the fourth poplar in Moscow after three intersectional hybrids, which are described below (Nasimovich et al., 2019).

***Populus deltoides*** W.Bartram ex Marshall [Deltoid poplar] – 1 sample from the Moscow landscaping, from Sheremetyevskaya street. It is a representative of the variable American species *P. deltoides* s.l., within which some researchers distinguish a number of independent species confined to different regions of North America. For example, A. Rehder (Rehder, 1949) considers four geographical forms, which sometimes appear as independent species, and, in addition, he lists another eight American species from this section that are close to them. Probably, this situation is similar to the attempts to distinguish numerous species within *P. suaveolens* in our country (see below). In its homeland, it is mainly a poplar of plains (Rehder, 1949). Probably, high geographical variability can be explained by the varying climate of different parts of the poplar area (transitions from the sea climate to the continental one, from warm southern – to cold northern), as well as the influence of the Appalachian Mountains in the center of the main area of *P. deltoides* s.l.

***Populus nigra*** L. (*P. nigra* var. *nigra*) [Black poplar]. A typical wild form of *P. nigra* with a wide spreading crown. The natural area of *P. nigra* is very extensive, covers a significant part of Europe and Asia (Borodina et al., 1966), but, nevertheless, the species is surprisingly monolithic over most of the habitat and is characterized by almost the same morphological features; and there were no attempts to distinguish other species or subspecies within *P. nigra* (unless cultivars are taken into account). Such monolithicity can be associated with the plain character of the species habitat, where there are no such geographical obstacles as mountains or seas. *P. nigra* is most characteristic of plain poplars in the world, and its features are the maximum expression of the features of section *Aigeiros* (Nasimovich and Vasilieva, 2018; Nasimovich et al., 2019). However, on the southern and eastern borders of the habitat, *P. nigra* is in contact or almost in contact with several species of mountain poplars, and the corresponding hybrids and hybrid species occur there (see *P.* × *irtyschensis*). The overlapping of the poplar area with that of *P. laurifolia* is especially great (Altai, Western Sayan) (Kostina et al., 2018). Additionally, *P. nigra* is in contact with numerous poplar species and hybrids of urban landscaping within its habitat, which largely violate its “purity”. For example, extensive hybridization of *P. nigra* with *P.* × *sibirica* occurs in Novosibirsk in the floodplain of the Ob river (Kostina et al., 2016) and in Novokuznetsk in the floodplain of the Tom river (Proshkin and Klimov, 2017). There is evidence that there are hardly any “pure” *P. nigra* samples in Western Europe (Cagelli et al., 1995; Broeck et al., 2004; Brus et al., 2010), but in Russia, except in large cities near large rivers with natural river territories inside the city, such hybridization is almost absent (Nasimovich et al., 2019); therefore, now we can still study the natural genotype of *P. nigra*. For our analysis, we took 22 samples from Russia, Central Asia, and Italy that were originally determined to be *P. nigra* by us or other researchers. Later, it turned out that only a few of them were collected outside the natural areas of other poplars from sections *Aigeiros* and *Tacamahaca*, and, therefore, can be rather confidently considered “pure” *P. nigra*. Thus, four samples from two points in the Orenburg Region (Vasilevka, Aituar steppe) can be considered “pure” *P. nigra*. Four samples from Italy (from the outskirts of Parma) collected by I.A. Schanzer are also important. The other 12 samples are visually close to *P. nigra*, but were collected in the area of contact with *P. laurifolia*, and, therefore, can be hybrids close to *P.* × *irtyschensis*. This type of plant material is presented by 8 samples from three points in the Novokuznetsk Region (Mitino, Upper Ters river, Slavino), 2 samples from one point in the Krasnoyarsk Territory (Verkhneimbatsk), 2 samples from one point in the Republic of Khakassia (Abakan river; collected by I.A. Schanzer). Another 2 samples (from Uzbekistan) may belong to *P. afghanica*; however, they are similar to *P. nigra* (collected in the natural habitat of *P. afghanica* and outside the natural habitat of *P. nigra*; these two species are not always distinguishable by one shoot in the herbarium).

***Populus nigra* × *Populus pyramidalis*** Rozier (*P. nigra* var. *nigra* × *P. nigra* var. *italica* Munchh.). Three samples from Moscow landscaping were collected on two different streets. Northern black pyramidal poplar, a set of cultivars. The cultivars were obtained by crossing the wild form of *P. nigra* (with a spreading crown) with the old cultivar *P. pyramidalis* Rozier (with a narrow pyramidal crown), which is widespread in the south of Russia and beyond its borders but cannot grow at the latitude of Moscow. According to several researchers (Komarov, 1936; Sokolov et al., 1951), *P. pyramidalis* [*P. nigra* var. *italica* Munchh.; *P. nigra* var*. pyramidalis* (Rozier) Spach; *P. italica* Du Roi] [Pyramidal poplar, Italian poplar, Lombardy poplar, Raina] originated from Afghanistan and then it spread throughout the Mediterranean; however, P.L. Bogdanov (Bogdanov, 1965) considered the Himalayas as its origin place. Recently, this poplar has been considered only a cultivated pyramidal form of *P. nigra* (Skvortsov, 2010), but, according to our perceptions, it is an intersectional hybrid with little involvement of some of “balsam” poplars (likely *P. talassica*), which is proved by pubescence of individual petioles, although weak, as well as the roundness of leaf blades compared to those of *P. nigra* var. *nigra* (our observations). Therefore, it is more convenient for us to consider *P. pyramidalis* and *P. nigra* separately. Nevertheless, *P. pyramidalis* is very close to *P. nigra*, and, hence, is considered a representative of the section of “black” poplars. As for the hybrid *P. nigra* × *P. pyramidalis*, it is definitely a representative of this section. We have not determined what cultivar of *P. nigra* × *P. pyramidalis* is spread in Moscow, but all black pyramidal poplars in this city are characterized by a narrow pyramidal crown and are very similar to each other. The attempts to create such a hybrid were made by many Russian breeders and had different outcomes (Kotelova and Stelmakhovich, 1963; Recommendations, 1976). Black pyramidal poplar #6 by V.T. Bakulina ((*P. nigra* × *P. pyramidalis*) × *P. nigra* (backcross hybrid)) was created in Siberia and used in Novosibirsk; therefore, its finding in Moscow is not very likely. Much the same is true for the hybrid #121 by A.M. Berezina from Bashkiria. In addition, it has a semi-pyramidal crown (Recommendations, 1976). Spontaneous hybrid *P. × charkowiensis* R.I.Schrod. (descr. ross., nom. inval.) [Kharkov poplar] is considered to have been preserved only in Kharkov already in the middle of the XX century, and its crown was different from a narrow pyramidal one (Kotelova and Stelmakhovich, 1963; Redko, 1966). Probably, cultivars of A.S. Yablokov, created in Ivanteevka near Moscow, are more common for Moscow. However, V.A. Bryntsev et al. (Bryntsev et al., 2019) indicated that the cultivars ‘Michurinets’, ‘Maxim Gorky’, and ‘Russian’ were relatively short-lived and were removed from urban landscaping at the age of 50-60, i.e. several decades ago. Within these varieties, only ‘Michurinets’ had a narrow pyramidal crown (Kotelova and Stelmakhovich, 1963). As for another variety by A.S. Yablokov ('Pioneer'), it does not have a narrow pyramidal crown (Kotelova and Stelmakhovich, 1963), it develops a rather spreading crown with age and, therefore, is prone to windbreak, which is why it is also removed from urban landscaping currently (Bryntsev et al., 2019). If the black pyramidal poplars by A.S. Yablokov are really absent or extremely rare in the landscaping of Moscow, then the only candidate for Moscow cultivar of *P. nigra* × *P. pyramidalis* is left*.* It is the black pyramidal poplar by A.V. Albensky. It is also obtained by crossing *P. nigra* var. *nigra* with *P. pyramidalis*. It has almost no specific features: the crown is narrow, columnar; the trunk is straight; the bark is light or blackish; the base of the leaf blade is varied (rounded, cuneate, truncate or cordate); venation is pinnate. This pyramidal poplar is winter-hardy to the line of St. Petersburg-Moscow-Samara (Kotelova and Stelmakhovich, 1963); therefore, its cultivation in Moscow is not excluded.

**Section *Tacamahaca* Spach [“Balsam” poplars]**

The section mainly combines mountain poplars, and all diagnostic features of the section (terete (rounded in cross-section), short and, as a rule, pubescent petioles with an adaxial groove; lanceolate, ovate, and other leaf blades; etc.) can be explained by the growth of trees in mountain river valleys (Nasimovich et al., 2019). Some species of this section grow outside the mountains, but this is associated with the culturing.

***Populus koreana*** Rehder [Korean poplar]. Five samples from four natural areas of the Primorsky Territory were studied. It is believed that *P. koreana* is different from *P. suaveolens* in strongly leathery leaves, pubescence of the midrib at the abaxial side, and glandular-adhesive axes of young shoots (Kotelova and Stelmakhovich, 1963); while from *P. maximowiczii*, it is different in angular (not flat) leaves, as well as the aroma of buds and young leaves (Komarov, 1936). In addition, the leaves are matte and the midrib is glabrous on the adaxial side (Kotelova and Stelmakhovich, 1963). *P. koreana* is common for the Amur Region, Primorye, and Sakhalin, but was first described in Korea (Kotelova and Stelmakhovich, 1963). A.K. Skvortsov and N.B. Belyanina (Skvortsov and Belyanina, 2006) believed that there are no morphological gaps between these three “species”, and in any part of their common range there are accessions with any set of the listed characteristics in different proportions, i.e., there is only intraspecific geographical variability, which is not enough even for the status of a subspecies.

***Populus laurifolia*** Ledeb. [Laurel poplar]. Eighteen samples from the natural area were studied: 11 from Gorny Altai, 2 from the Krasnoyarsk Territory (from the Western Sayan), 2 from the outskirts of Novokuznetsk (the Upper Ters river), 2 from Mongolia, 1 from Kazakhstan. It is pointless to talk about the complete “purity” of this species, since almost the entire habitat is located inside the natural area of *P. nigra* (Borodina et al., 1966). Nevertheless, the ecological niches are divided: *P. laurifolia* grows in the mountains, while *P. nigra* grows on the plains, but in the foothills they meet and form a hybrid *P. × irtyschensis*. Additionally, in the east of the habitat, *P. laurifolia* is in contact with *P. suaveolens*, or rather, it is immediately replaced by this species, when goes beyond the natural area of *P. nigra*. This allows us to consider *P. laurifolia* as a dynamic state of *P. suaveolens* in the conditions of a continuous gene flow from *P. nigra* (Nasimovich et al., 2019). According to the sectional features, *P. laurifolia* coincides with *P. suaveolens* (a typical representative of his section), according to the rest features – with *P. nigra* (at least, according to these features, partially evades from *P. suaveolens* and becomes closer to *P. nigra*). Nevertheless, *P. laurifolia* is not a hybrid, but a hybridogenic species, since it has at least one feature that does not exist in both parental species – strongly grooved axes of 1-2-year-old shoots. The dual nature of *P. laurifolia* (independent species, dynamic state at the intersection of gene flows from two other species) should be taken into account when interpreting the results of molecular genetic analysis.

***Populus longifolia*** Fisch. (IPNI, Tsinovskis, 1977; Tsvelev, 2001) – *P*. *longifolia* Fisch. ex Loudon, nom. inval. (IPNI) – *P*. *candicans* var. *elongata* Dippel (Dippel, 1892) – *P*. *balsamifera* var. *elongata* hort. (Karhu and Hamet-Ahti, 1992) – *P*. *tristis* var. *longifolia* (Fisch.) A.K.Skvortsov, descr. ross., nom. inval. (Skvortsov, 2008). We considered *P. longifolia* as an independent Russian and generally European species, separately from *P. tristis* Fisch. [*P. tristis* var. *tristis*] [Sad poplar]. Six samples (and 3 more samples with doubts in identification) from Moscow were studied: 3 from two points of street landscaping, 3 from the MHA territory (planted near the Laboratory building; perhaps hybrids with large involvement of *P. longifolia*), 3 from the Shchukinsky peninsula (sandy natural area near the Moscow river, the most typical representatives of this species, male clone). The ambiguous attitude of modern botanists to this species forces us to make several comments regarding its systematic position and origin. Here are the main versions of its origin: 1) one of a form of *P. balsamifera*, which was brought to Russia from North America through Western Europe (common view in the recent past (Dippel, 1892; Karhu and Hamet-Ahti, 1992)); 2) natural species of Central Asia (Rehder, 1949); 3) one of the separate American clones of *P. trichocarpa* Torr. et A.Gray ex Hook. from sucker shoots (Skvortsov, 2008); 4) separate East Siberian clone of *P. suaveolens* from sucker shoots (Mayorov et al., 2012; our assumption); i.e., the origin of *P. longifolia* is not clarified. Currently, *P. longifolia* is sporadically found in the northern half of the European Russia and often in the natural environment. This is the only adventive type of poplar that is prone to complete naturalization (Mayorov et al., 2012). The tree is up to 15-18 m in height, very often with an inclined trunk. It gives abundant root suckers. The axes of the 1-2-year-old shoots in the crown of an adult tree are brown, reddish-brown, dark in color, terete or slightly angular in the section. The terminal buds are large, up to 3.5 cm long, but usually 2-2.5 cm. Axillary buds are up to 2 cm long. Petioles are on average longer than those of other “balsam” poplars – up to 5-8 cm, but petioles are not so large relative to long leaf blades – 1.5-3 times shorter than leaf blades; often terete, with a wide or narrow adaxial groove. Leaf blades are quite large, up to 10-14 cm long and up to 6-7 cm wide, although usually much narrower than (the length exceeds the width by 2-3 times); lanceolate or wide-lanceolate; maximally expanded in the middle, less often with shift to the base or apex. The base of the leaf blade is rounded (in the largest leaves) or cuneate, cordate bases do not occur practically (the difference from *P. tristis* Fisch.); the base smoothly passes into a slightly convex lateral edge. The margin of the leaf blade is serrate or crenate (2-5 teeth per 1 cm), without a narrow transparent border. The apex of the leaf is acute or attenuate with 1-5 mm tip, sometimes only acute (an important difference from *P. suaveolens*). The adaxial side of leaves is dark green (almost black-green), the abaxial side is whitish, and this contrast is the most important distinguishing feature of the species, marks its hybrids. The glands at the petiole near the base of the leaf blade are absent or poorly marked as eroded or densely pubescent areas. The leaf blades of leaves of annual shoots are almost the same, although more often lanceolate, with a cuneate base, and less often with a rounded one; up to 15 cm long, the length is 2-4 times larger than the width. The leaves of root suckers can be much larger, and the maximum expansion is sharply shifted toward the base (MHA). The petioles are poorly pubescent, 3-6 cm long, 4-5 times shorter than leaf blades. Leaf blades are large, up to 17 cm long and up to 10 cm wide (the length exceeds the width by 1.5-2 times); ovate; maximally expanded in 1/4-1/3 of the leaf blade from the base. The base is rounded or cuneate. At the petiole near the base of the leaf blade, there are often large glands (description of Yu.A. Nasimovich in nature and according to herbarium samples in the MHA). Capsules are glabrous, almost sessile, open with 3 valves (Kostina and Nasimovich, 2012).

***Populus maximowiczii*** Henry [Maximovich poplar]. Eight samples from six natural areas of the Primorsky Territory were studied. It is believed that *P. maximowiczii* is different from *P. suaveolens* in slightly leathery leaves, pubescent midrib (on both abaxial and adaxial sides) (Kotelova and Stelmakhovich, 1963), and from *P. koreana*, it is different in flat leaves, as well as almost odorless buds and young leaves (Komarov, 1936). Additionally, the leaves of *P. maximowiczii* are glossy, and the midrib is pubescent on adaxial side (Kotelova and Stelmakhovich, 1963). This poplar is common for the Amur Region, Primorye, and Sakhalin (Kotelova and Stelmakhovich, 1963). A.K. Skvortsov and N.B. Belyanina (Skvortsov and Belyanina, 2006) believed that there are no morphological gaps between these three “species”.

***Populus simonii*** Carriere [Simon poplar, Chinese poplar, Przewalski poplar]. The likely origin place of the wild form of *P. simonii* is Northern China (Rehder, 1949) and the neighboring regions of Russia – Tarbagatai, Dzungarian Alatau (Kachalov, 1970). Of the clones cultivated on the Russian plain, two are the most common, which are antipodes in many ways: *P. s. fastigiata* C.K.Schneid., f. – pyramidal or wide-pyramidal poplar with obovate leaves; *P. s. subpendula* (the same as *P. pendula* C.K.Schneid., f.) – a semi-weeping poplar with lanceolate leaves.

*P. s.* f. *fastigiata* is characterized by porrect strongly angular annual branches with 4-5 faces. The petioles are short, with a length of 0.3-1.2 (up to 2) cm. The leaf blades are obovate, less often lanceolate, with the maximum expansion sometimes located in the middle, but often strongly shifted to the apex. The base of leaf blades is always attenuate. Margin without cilia, with 6-9 teeth per 1 cm. The leaf apex is obtuse or acute, to some extent apiculate with 0.5-3 mm tip. One sample of *P. s.* f. *fastigiata* from the MHA was studied.

*P. s.* f. *subpendula* is characterized by the ascending upper branches (directed up and in breadth), as well as the lower pendulous branches (hanging down in the form of lashes) up to 1 m long. The lower branches have numerous short shoots. The petioles of the leaves from these shoots are relatively long – from 0.5 to 4 cm. Leaf blades are lanceolate with the maximum extension in the middle or (less often) slightly closer to the apex. The base of leaf blades is cuneate. The margin has rare cilia and 3-5 teeth per 1 cm. The leaf apex is attenuate with 5-10 mm tip. Seven samples of *P. s.* f. *subpendula* from landscaping of the Moscow region were studied: 4 from two points in Moscow and 3 from one point in the Moscow Region.

***Populus suaveolens*** Fisch. [Fragrant poplar], including *P. koreana* Rehder, *P. maximowiczii* A.Henry, and less famous *P. baicalensis* Kom., *P. usuriensis* Kom. (Komarov, 1936), and also *P. komarovii* J.J.Vassil. ex Vorosch. that was described in 1965 (Skvortsov, 2010). The areas of *P. koreana* and *P. maximowiczi* almost coincide in Russia (Borodina et al., 1966), and the area of *P. usuriensis*, judging by the herbarium specimens (MHA), is also located here, which seems very strange, taking into account biotopic identity (bottom of valleys of mountain rivers) and the ease of hybridization in poplars. Also some of the specified “species” are described according to the single herbarium specimens of other researchers (Komarov, 1936), which, taking into account the variability of poplars, seems to us erroneous. Generally, we completely agree with A.K. Skvortsov and N.B. Belyanina (Skvortsov and Belyanina, 2006) in the inappropriateness of the allocation of these “species” and even “subspecies”. Nevertheless, in our work, we separately consider samples defined by Russian botanists as *P. suaveolens, P. koreana,* and *P. maximowiczii* to ensure that they do not differ at the genetic level. When considered separately from all or at least some forms (*P. koreana, P. maximowiczii*), the features of *P. suaveolens* are relevant in the narrow sense: the leaves are not leathery and wrinkled, midrib without pubescence; young branches are not glutinous; these forms prevail in the East Siberia and in the Far East, north of the Amur river (Kotelova and Stelmakhovich, 1963). Two samples from the MHA and 36 samples from the natural area of *P. suaveolens* were studied: 10 from Transbaikalia (Chita), 3 from Yakutia, 20 from the Khabarovsk Territory, 1 from the Magadan Region, and 1 from Mongolia, i.e. there almost were no samples from the Primorsky Territory. This is important since it allows to compare *P. suaveolens* in the narrow sense with two close species from the Primorsky Territory and, at least, to evaluate the geographical variability within the generalized area of *P. suaveolens* *sensu lato*. Additionally, there are enough samples for comparison of *P. suaveolens* from Transbaikalia (10 samples) and Khabarovsk Territory (20 samples).

***Populus talassica*** Kom. [*P*. *macrocarpa* (Schrenk) Pavlov et Lipsch.], [Talas poplar]. The native range covers Kyrgyzstan, Uzbekistan, as well as Afghanistan and part of China’s territory. *P. talassica* is similar to *P. laurifolia*: crown is spreading; axes of the 1-2-year-old shoots are grooved; the leaf blades are lanceolate with cuneate base and acute apex (MHA). It has a set of features of “balsam” poplars with a small “shift” toward “black” poplars (Nasimovich and Vasilieva, 2019; Nasimovich et al., 2019). Ten samples of *P. talassica* from Central Asia (MHA) were studied: 5 from two points of Kyrgyzstan and 5 from four points of Kazakhstan (possibly, accessions from Kazakhstan were planted).

**? *Populus trichocarpa*** Torr. et A.Gray ex Hook. [*P. balsamifera* subsp. *trichocarpa* (Torr. et A.Gray) Brayshaw] [Black cottonwood]. The natural range includes the west of North America from Alaska to California (Rehder, 1949). P. *trichocarpa* is different from other poplars of its section, first of all, by pubescent capsules, although, in some cases, *P. laurifolia* can also have such capsules (our observations). It has leaf blades of various shapes, which often have a truncate base; at least, the maximum expansion of the leaf is very strongly shifted toward the base; the leaf blade is sometimes almost deltate, albeit with rounded lateral edges, which is not typical for the Russian representatives of this section. However, the shape of the leaf does not always differ from the American *P. balsamifera* L., but it differs well from it in three-valve pubescent capsules (in *P. balsamifera* they are two-valve and glabrous). We have 4 samples from Moscow, which may belong to *P. trichocarpa* or its hybrids with its large involvement. Three samples were collected on the Shchukinsky peninsula in Moscow (on a sandy natural area), where there is a planted or self-sowing group of several middle-aged trees with three-valve and strongly pubescent capsules and numerous root suckers. Presumably, this is a hybrid cultivar *P.* × *trichocarpa* ‘Lettland’ [*P.* × ‘Lettland’] [Latvian poplar]. Its origin has not been established, but it is clear that one of the parental species is *P. × trichocarpa*; it is distributed in the Baltic States and Scandinavia, often appears in Western literature as a “pure” *P.* × *trichocarpa*, which is erroneous (Tsinovskis, 1977). Other features of this poplar from the Shchukinsky peninsula: trees with a spreading crown and light whitish (almost white) trunks, has a complex of features of “balsam” poplars (the petiole is terete with a clearly visible adaxial groove and etc.), adaxial and abaxial sides of the leaf blade have large color contrast (green and whitish respectively), but in terms of the shape of the leaf blade, these poplars are not similar to the North American collections of A.K. Skvortsov, stored in the MHA: leaf blades are very similar (slightly variable), ovate, with attenuate leaf apex; the base of the leaf blade is rounded and somewhat irregular, as in *P. × sibirica*. Another sample of *P. × trichocarpa* ‘Lettland’ was collected on the MHA territory and was listed in the documents as *P. × trichocarpa*. This sample was less similar in leaf blade shape to *P. × sibirica*, but also differs from the American samples of A.K. Skvortsova. Its capsules were also pubescent and three-valve.

***Populus* × *wobstii*** R.I.Schrod. One of the spontaneous hybrids [“Shroeder species”], spontaneously which arose at the turn of the XIX and XX centuries on the MTAA territory near Moscow (now in Moscow). There is no typical herbarium sample, and good descriptions were not made in time. We currently attribute this name to the hybrid of two Russian narrow-leaved “balsam” poplars: *P. laurifolia* and *P. longifolia* (Mayorov et al., 2020), i.e., we imply the set of the corresponding hybrids, and not a specific cultivar. Nevertheless, the views of different researchers on the original *P.* × *wobstii* differed sharply: a variety of *P. suaveolens* (Regel, 1889); a variety of *P. balsamifera* (Dippel, 1892); *P. suaveolens* × *P. jackii* (Sokolov et al., 1951); *P. simonii* × *P. suaveolens* (Karhu and Hamet-Ahti, 1992); *P. laurifolia* × *P. tristis* (Rehder, 1949; Koltzenburg, 1999); *P. laurifolia* × *P. tristis* (or *P. longifolia*) (Ascherson and Graebner, 1908; Tselov, 2001). Generally, *P.* × *wobstii* is similar to *P. laurifolia*: the axes of 1-2-year-old shoots of undergrowth and root suckers are light and grooved, and the leaves on them are relatively narrow (lanceolate, narrowly-lanceolate). In the crown of adult trees, only slightly angular and less light axes of shoots (grayish-beige) prevail, and the leaves on them, as a rule, have rounded-cuneate or rounded bases and longer petioles (only 2-4 times shorter than leaf blades). From the highly variable *P.* × *moscoviensis* R.I.Schrod. ex Wolkenst. (*P. laurofolia* × *P. suaveolens*), *P.* × *wobstii* is distinguished by the long petioles, as well as “neatness”: the leaves of mature trees are approximately the same shape and size, all relatively large and elongated. The difference in the color of the adaxial and abaxial surfaces of the leaf is also slightly larger. The differences of *P.* × *wobstii* from *P. longifolia* are even more: the axes of the shoots are angular, the tree is high (up to 20-25 m), the trunk does not incline with age, and there are no abundant root suckers. Three samples of *P.* × *wobstii* from two points of the Moscow Region were studied.

***Aigeiros* × *Tacamahaca* [intersectional hybrids of “black” and “balsam” poplars]**

Representatives of this group have intermediate features of two sections (the petiole, for example, is slightly laterally flattened, but has an adaxial groove, although narrow and interrupted). This group includes the most widespread poplar hybrids (hybridogenic species, spontaneous hybrids, cultivars) of urban landscaping. They often grow spontaneously on wastelands, along railroads and in other similar places, and, therefore, it is especially surprising that they are not in regional guides and “floras”; the trees are little known to professional botanists and urban landscaping workers. Some of them are known under erroneous names in Russia and sometimes beyond its borders. These names may not coincide in Russia and other countries, be different in the understanding of different authors within the same country, and, therefore, we provide the main diagnostic features of these hybrids, and sometimes complete descriptions.

? *Populus* × *berolinensis* K.Koch [*P. laurifolia* × *P. pyramidalis*] – see *Populus* × *petrovskoe*.

***Populus* × *canadensis*** */* ***P*. × *sibirica***. A hybrid of these two hybrids or one of these hybrids, but with a morphological bias toward another hybrid, which is associated with the specifics of growing conditions. One sample from Moscow landscaping was studied.

***Populus deltoides* × (*P. laurifolia* × *P*. *suaveolens*)** – an unnamed complex hybrid that crosses with *P.* × *jackii* Sarg. or with *P.* × *angulata* Aiton; simultaneously, it especially often appears among landscapers as “large-leaved poplar” – *P. candicans* Aiton, i.e., the same *P.* × *jackii*. If the issue of parental species is considered in the literature, then *P. deltoides* almost always appears as the “black” component, and *P. laurifolia* or *P. suaveolens* is considered as the “balsam” component, but we decided that “balsam” component came from a hybrid between these two species, namely *P.* × *moscoviensis* R.I.Schrod. ex Wolkenst. If it was a hybrid of *P. deltoides* only with *P. laurifolia*, then the leaf blade bases would be predominantly truncate, and if it was a hybrid of *P. deltoides* only with *P. suaveolens*, the leaf blade bases would be predominantly cordate. Rounded leaf blade bases indicate the involvement of *P.* × *moscoviensis*. The same is evidenced by the variability of the shoot axes in terms of their angularity and the slightly attenuate apex of the leaf (for *P. laurifolia,* apex of the leaf is acute, but not attenuate, for *P. suaveolens,* it is acuminate). *P.* × *moscoviensis* at the beginning of the 20th century was the main poplar in the landscaping of Moscow (Syreyshchikov, 1907; Nasimovich et al., 2019), and, therefore, its involvement is most likely. The nomenclature issue cannot yet be considered resolved, but poplars with large rounded leaves are occasionally found in landscaping, and we still do not see the possibility of naming them as something else. Here is a more complete description of them. The crown is spreading. Axes of 1-2-year-old shoots are almost terete or slightly angular, sometimes grooved, with terminal and axillary buds up to 2 cm long. Petioles are from 3 to 7 (up to 9) cm long, 1.8-2.5 times shorter than leaf blades, slightly flattened laterally; on adaxial side, as a rule, with a narrow and usually interrupted groove, less often without a groove. Leaf blades are large, up to 12-14 cm in length and up to 10 cm in width (the length exceeds the width by an average of 1.5 times), ovate or broadly ovate, with a maximum expansion strongly shifted toward the base (1/4-1/3 of the leaf blade from the base). Leaf blade base is cuneate with subsequent rounding or rounded, rarely cordate, cordate-truncate or has irregular shape (as in *P.* × *nevensis*), very smoothly passes into a convex lateral edge. Margin is crenate or serrate (1.5-5 teeth per 1 cm, sometimes 1 tooth per 1 cm at the base), without cilia or with sparse cilia closer to the apex, with a narrow (0.1 mm) translucent border. The apex is attenuate with 1-2 cm tip. The adaxial surface of the leaf is light green, the abaxial surface is whitish-grayish-greenish, but the difference in color is not very great. The midrib is light yellowish-greenish, up to 1 mm wide, does not stand out sharply on the adaxial surface of the leaf. On average 5 (max. 7) veins extend from the base of the leaf blade. There is a pair of glands at the petiole near the base of the leaf blade in most cases, not on all leaves however. Leaves do not turn yellow or fall off for a long time in autumn. Leaf blades of annual shoots are ovate, rarely broadly ovate or deltate, up to 12-16 cm in length and up to 10-12 cm in width, with a cordate-truncate, cordate-rounded or rounded base. Capsules, as a rule, open with 3 valves. Four samples from two points of Moscow landscaping were studied.

***Populus deltoides* × *P*. *suaveolens***. One sample from Moscow landscaping with characteristics intermediate between the indicated species was studied. It is very similar to the American *P.* × *jackii* (*P. deltoides* × *P. balsamifera*) (large leaf blades with a cordate base, shaped like the leaves of *Tilia cordata* L.), but the petioles are quite often very densely pubescent, which in not typical for *P.* × *jackii*.

***Populus* × *irtyschensis*** Chang Y.Yang [*P.* × *berolinensis* nothovar. *irtyschensis* (Chang Y.Yang) C.Shang; *Populus laurifolia* × *P. nigra* var. *nigra*] [Irtysh poplar]. As an admixture to parental species, it occurs everywhere in nature at the points of contact between *P. laurifolia* and *P. nigra* var. *nigra* (Kemerovo Region, Novosibirsk Region, south of the Krasnoyarsk Territory, along the Irtysh river outside Russia), and in some places it forms “pure” populations (without parental species), and then it can be considered a hybrid species. We found samples in the MHA herbarium, which, according to the main features, can be recognized as hybrids of these two species, and made a more detailed description of them. In addition, this description was compared with samples from the natural range (from Siberia) brought by M.V. Kostina, and it corresponded to them. Axes of 1-2-year-old shoots are relatively light, from terete to strongly grooved, with medium-sized buds up to 1 cm in length. Petioles of leaves from brachyblasts (short shoots) are slightly pubescent, 0.5 to 4 cm in length, 1.5-3 times shorter than the leaf blade, usually slightly flattened laterally, with a narrow interrupted groove (less often without a groove). Leaf blades from brachyblasts from the crown of an adult tree are glabrous, up to 7-8 cm long, up to 5-6 cm wide (the length is 1.5-, less often almost 2-, times larger than the width), ovate-lanceolate, slightly rhombic-like with maximum expansion strongly or slightly shifted toward the base if the leaf blade is large, or the maximum expansion is almost in the middle if the leaf blade is small. The base of leaf blade is cuneate, almost rounded or irregular (rounded at the petiole, then cuneate), smoothly turning into a slightly convex or rounded lateral edge. The margin is crenate-serrate (4-7 teeth per 1 cm, at the base – 1-3 teeth per 1 cm), with a narrow translucent border, with sparse cilia or without cilia. The leaf apex is acute or attenuate. If there is an apical tip, then it is about 1 cm long. The adaxial surface of the leaf is light green, the abaxial surface is grayish-greenish, but the difference in color is not very great. The midrib on the adaxial side of the leaf is pale yellowish, up to 0.5 mm wide. On average 5 (min. 3) veins depart from the base. Glands at the petiole near the base of the leaf blade are usually absent (MHA). Capsules open with 2 or 3 valves. In general, there are few specific features, and you can recognize this hybrid by ignoring other hybrids and cultivars that have more characteristic features. The similarity with *P.* × *sibirica* is especially great, but *P.* × *sibirica* more often has an irregular base of the leaf blade, and grooved shoot axes are less typical. In addition, the leaves of *P.* × *irtyschensis* are more variable. Seven samples collected at two points in Novokuznetsk (Kemerovo Region) were studied.

***Populus longifolia*** / ***P.* × *sibirica*** or ***P*. *longifolia* × *P.* × *sibirica***. A hybrid of the above two species or one of the species with morphological deviation toward another species. Three samples from one point in Moscow were studied.

***Populus nigra* × *P*. × *sibirica***. Samples with characters strictly intermediate between *P. nigra* and *P. × sibirica* were observed, in particular, in Novosibirsk (Kostina et al., 2016), Novokuznetsk (Proshkin and Klimov, 2017), and Irkutsk (Kostina et al., 2018), Saratov (Kostina and Nasimovich, 2018); and these hybrids are common within the natural range of *P. nigra*. We studied eight samples from Novosibirsk.

***Populus* × *petrovskoe*** R.I.Schrod. ex Wolkenst. [*P.* × *petrowskiana* R.I.Schroed. ex Dippel;]. Known to Russian landscapers under the erroneous name *P.* × *berolinensis* K.Koch, but the real *P.* × *berolinensis* may not be present in Russia at present. One of the spontaneous hybrids discovered by R.I. Schroeder on the MTAA territory (Petrovsky Academy in those times) at the end of XIX century. More often is mentioned as *P.* × *petrowskiana* R.I.Schroed. ex Dippel, but the corresponding monograph (Dippel, 1892) appeared a decade later than Wolkenstein's article (Wolkenstein, 1882) signed only with the initials (P.W.), and, in addition, L. Dippel considered this hybrid as one of the forms of *P.* × *canadensis* with which we disagree. We consider *P.* × *petrovskoe* as a hybrid of *P.* × *canadensis* and *P. laurifolia*, and the corresponding arguments were given earlier (Mayorov et al., 2020). Other versions about the parent species of *P.* × *petrovskoe* are the following: *P. deltoides* s.l. × *P. suaveolens* (Wolkenstein, 1882; Regel, 1889); *P. deltoides* × *P. jackii* (Dippel, 1892); *P.* × *canadensis* [or also *P. deltoides*?] × P*. suaveolens* (Bogdanov, 1965); *P. deltoides* × *P. laurifolia* (Karhu and Hamet-Ahti, 1992; Koltzenburg, 1999; Tsvelev, 2001); *P.* × *canadensis* × *P. laurifolia* (Skvortsov, 2010), but in relation to *P.* × *berolinensis*, for which he mistook this cultivar). M. Wolkenstein (Wolkenstein, 1882) gave the following description of *P.* × *petrovskoe*: “A hybrid between *P. canadensis* fertilised by the pollen of *P. suaveolens*. The leaves are large rhomboid, young shoots winged.”. The involvement of *P. suaveolens* instead of *P. laurifolia* is Wolkenstein's obvious mistake. *P. suaveolens* rounds the leaves of its hybrids and they cannot be rhombic-like, and without *P. laurifolia* the shoot axes will not be grooved. But otherwise this brief description is sufficient to recognize *P.* × *petrovskoe* if one considers the main cultivars used in Moscow. Here is a detailed description of *P.* × *petrovskoe*, which was made in 2010 by Yu.A. Nasimovich using the example of a powerful old accession in front of the facade of the MTAA main building (the corresponding herbarium sample on several sheets was transferred to the MHA herbarium: 08/31/2010, D.A. Medvedeva, Yu.A. Nasimovich), as well as the example of other accessions of *P. × petrovskoe* of different ages from landscaping in Moscow. This description is published in full for the first time. A slender tree up to 25 m high, with a very wide (sometimes the same as height) crown in youth, and then the crown becomes less wide and even semi-pyramidal. The branches of the first order are relatively straight and long, and can be up to half the height of the tree. A young tree is characterized by a low crown; the lower branches depart from the trunk almost horizontally or slightly upwards (ascending), the middle ones come upwards at an angle of about 45 degrees, the upper branches are almost appressed, due to which the crown turns out to be globose in the first approximation, although uneven, and in the section it is widely flabellate. As the lower branches die off, the crown becomes elongated upwards and almost semi-pyramidal. If the trees are planted closely and depressed, then the crown looks pyramidal, but the branches of the lower and middle parts of the tree are ascending or porrect (directed sideways or obliquely upwards), and not appressed, as in true pyramidal trees. Branches of the second order are directed mainly upwards. In old trees, the trunk from the very ground is densely covered with vertical sucker shoots from axillary buds, because of which the crown seems to be lowered low and expanding sharply closer to the top of the tree. The tree is surrounded by root suckers of different heights if it is not removed from the lawn by landscapers. Young annual branches from the crown are slightly pubescent, light beige, angular or grooved, with terminal buds up to 1.5 cm long. Short shoots (brachyblasts) from the crown are also slightly pubescent. Petioles of leaves from a brachyblasts are slightly pubescent, 1.5 to 6 cm long, slightly or 2-3 times shorter than the leaf blade, slightly flattened laterally, with a narrow interrupted adaxial groove. Leaf blades are glabrous or with rare hairs along the veins, medium in size, up to 9 (rare 12) cm long and up to 8 (rare 10) cm wide (the length slightly exceeds the width, usually 1.2 times, rarely exceeds it by 1.5 times or inferior to it). Large leaf blade are deltate-ovate, and small ones are ovate or rhombic-like as in *P. nigra*; all leaves, as a rule, with a maximum extension of 1/3 of the leaf blade from the base. Leaves from brachyblasts have a very characteristic appearance that other cultivars do not have. The base of the leaf blades is rounded-truncated or cuneate, uneven, very often with two symmetrically located shallow notches, smoothly passes into a slightly convex or almost straight lateral edge. The margin is crenate-serrate (4-5 teeth per 1 cm), with a narrow (0.1 mm) translucent border and sparse cilia. The apex is attenuate with 0.5-1.5 cm tip (Figure, panel B). In comparison with the leaves of *P.* × *sibirica*, the leaves of *P.* × *petrovskoe* are shorter (length is 1.2 rather than 1.5-2 times greater than width), but otherwise very similar. The adaxial surface is light green, the abaxial surface is even lighter, but the difference in color is not very great. The midrib is pale yellowish-greenish, up to 0.6 mm in width. Three-five veins extend from the base. The glands at the petiole near the base of the leaf blade are usually absent in leaves in the crown. The foliage turns yellow and falls off much later than that of the Siberian poplar. The leaf blades of annual shoots in the crown of an adult tree can be as in *P.* × *sibirica* (relatively narrow, the length is 1.5-2 times greater than width), and at the apical part of the shoots they can be wide and cordate. Generally, leaves are very variable and not suitable for the identification of *P.* × *petrovskoe*. Leaf blades on shoots from dormant buds in the lower part of the trunk have the lanceolate shape, with a cuneate base. Root suckers are strongly grooved and with larger leaf blades – up to 17 cm long and up to 15 cm wide. Petioles reach a length of 8 cm, 1.5-2.5 times shorter than leaf blades, strongly flattened laterally. Leaf blades are cordate-deltate, with a maximum expansion of 1/8-1/6 of the leaf blade from the base, with a cordate or (less often) truncate-rounded base. The margin is crenate, often without cilia. The apex is acute or attenuate with 2-2.5 cm tip. There are often glands at the petiole near the base of the leaf blade. The second widespread poplar in Moscow after *P.* × *sibirica*. Since the middle of the XX century appears among Russian landscapers under the erroneous name *P.* × *berolinensis* K.Koch [*P. laurifolia* × *P. pyramidalis*]. However, the real *P.* × *berolinensis*, if it was in Moscow, had come out of landscaping by that time, and the name passed to the similar *P.* × *petrovskoe*. Michael Koltzenburg wrote about the similarity of *P.* × *berolinensis* with *P.* × *petrovskoe*, but *P.* × *berolinensis* has longer leaf blades and does not form root suckers (Koltzenburg, 1999). Fifty-seven samples (and 5 more samples with doubts in identification) from 18 points of the Moscow landscaping were studied.

**? *Populus* × *petrovskoe* × *P*. × *sibirica***. A hybrid of the above two cultivars, or a cultivar with a random morphological bias toward the other cultivar. Three samples from one point in Moscow were studied.

***Populus* × *rasumovskoe*** R.I.Schrod. ex Wolkenstein [*P.* × *rasumowskiana* R.I.Schroed. ex Dippel] More often appears in the literature as *P.* × *rasumowskiana* R.I.Schroed. ex Dippel, but the corresponding monograph (Dippel, 1892) appeared a decade later than Wolkenstein's article (Wolkenstein, 1882). One of the spontaneous hybrids discovered before 1882 by R.I. Schroeder on the MTAA territory near Moscow. M. Wolkenstein gave the following description: “A hybrid between *P. nigra*, fertilised with the pollen of *P. suaveolens*. A large tree, leaves roundish, smaller than in the previous variety [in *P.* × *petrovskoe*]. Shoots cylindrical”. This description was enough to recognize this cultivar. Let us give a more complete description made by Yu.A. Nasimovich on the basis of his own observations and the samples he collected, some of which were transferred to the MHA herbarium. The tree of medium height. The crown is spreading, uneven. The apical parts of many branches hang down, forming whips up to 0.5-1 m long, i.e., the crown is semi-weeping, although there are individual trees in which this feature is not expressed or is weakly expressed. Young branches (axes of 1-2-year-old shoots) are glabrous, light beige, terete or angular, but not grooved, with rather large glabrous beige buds: terminal – up to 1.8 cm long, axillary – up to 1.5 cm long. Axes of brachyblasts in the crown of an adult tree are slightly pubescent. Petioles of leaves on brachyblasts are slightly or strongly pubescent (sometimes seem to be strongly pubescent due to adhering fluff), from 1 to 4-5 (rare up to 7) cm long, 1.5-4 times shorter than leaf blades, flattened, terete or slightly flattened, without an adaxial groove or with a narrow interrupted groove. Leaf blades are glabrous or slightly pubescent along the veins, up to 8-9 (rare 11) cm long and up to 5-6 (rare 8) cm wide (the length slightly or 1.5-2 times greater than the width, but mostly beyond account of an acuminate apex region); ovate, slightly less often rounded or broadly ovate, with a maximum expansion strongly or slightly shifted toward the base (if we neglect the acuminate apex, then the maximum expansion is almost in the middle of the leaf blade). The base is cuneate or rounded, smoothly turning into a convex rounded lateral edge. The margin is crenate-serrate with obtuse teeth (3-4 teeth per 1 cm), with a narrow translucent border and sparse cilia. The apex is acuminate with 1-1.5 cm tip (may be 0.5-2 cm very rare) (Figure, panel C). The adaxial surface is light green, and the abaxial surface is grayish-greenish, but the difference in color is not very sharp. The midrib on the adaxial side of the leaf blade is pale green, up to 0.5 mm wide. The glands at the petiole near the base of the leaf blade are absent (MHA). In autumn, the foliage turns yellow and falls rather early, but later than in *P. × sibirica*. No capsules were observed (male clone cultivated). The third widespread poplar of Moscow landscaping, the second widespread poplar near the MTAA (Nasimovich et al., 2019), but for some reason since the middle of XX century does not appear in the botanical and landscaping literature of Moscow and Russia in general if we do not take into account our works of the last decade (probably mixed with *P. balsamifera* L., *P. suaveolens*, or *P.* × *moscoviensis* R.I.Schrod. ex Wolkenst.). Thirty samples (and 4 more samples with doubts in identification) from 12 points of the Moscow landscaping were studied.

**? *Populus* × *rasumovskoe* × *P*. × *sibirica***. A hybrid of the above two cultivars, or a cultivar with a random morphological bias toward the other cultivar. One accession from Moscow landscaping with intermediate features was studied.

***Populus* *suaveolens* × (*P. suaveolens* × *P*. *sp*.)**. Not rather “pure” *P. suaveolens*. It can be considered as a spontaneous hybrid of incomprehensible nature, characteristic of the city, with large involvement of *P. suaveolens*. Two samples from two points of the Moscow landscaping were studied.

***Populus* × *sibirica*** G.V.Krylov et G.V.Grig. ex A.K.Skvortsov [*P*. × *sibirica* G.V.Krylov, nom. inval.] The most widespread poplar of urban landscaping in Russia, which until recently was mistaken for *P. balsamifera* from the North America. At present, we consider it a Siberian hybridogenic species that arose in nature at the intersection of gene flows from three Siberian species – *P. nigra*, *P. laurifolia*, and *P. suaveolens*; the occurrence as a culture is also not excluded, but it is strange why it went unnoticed by botanists and breeders. Since the “black” and “balsam” components are approximately equal, the composition can be expressed as follows: *P. nigra* × (*P. laurifolia* × *P. suaveolens*) (Mayorov et al., 2012; Mayorov et al., 2020). The other views on the origin are the follows: the same as *P. balsamifera* L. ((Syreyshchikov, 1907; Albensky, 1954); current prevailing ideas of plant breeders, foresters, and landscapers); *P. balsamifera* × *P. laurifolia* (opinion of G.V. Krylov and N.G. Salatova in 1950 (Krylov, 1961)); *P. laurifolia* × *P. nigra* (opinion of G.V. Krylov and N.G. Salatova in 1952 (Krylov, 1961)); an independent natural Siberian species from the section of “balsam” poplars (Krylov, 1961); *P. balsamifera* × *P. nigra* (Yakushina, 1982; Skvortsov, 2007; Reshetnikova et al., 2010); *P. × moscoviensis* (*P. laurifolia* × *P. suaveolens*) × *P. nigra* (Mayorov et al., 2012; Mayorov et al., 2020). We give its description, since not all the main diagnostic features are reflected in the descriptions known to us. A tree up to 30 m in height. The crown is high, wide, spreading, irregular (as if “sloppy”, with separate large branches growing in the other direction than most branches grow in) [important, but not previously mentioned feature]. Young annual branches in the crown of an adult tree are light beige, brownish-grayish, rather light, terete or slightly angular (but in undergrowth and on powerful sucker shoots they can be grooved). Leaf petioles on brachyblasts are slightly pubescent, 1.5 to 4 cm long, 2-3 times shorter than leaf blades, slightly flattened laterally, without an adaxial groove or with a narrow interrupted groove [the most important feature, but it was noted by A.K. Skvortsov (Skvortsov 2007)]. Leaf blades are glabrous, medium in size, up to 8-10 cm long and up to 5-7 cm wide (the length exceeds the width by an average of 1.5 times, rarely 2 times); ovate, ovate-lanceolate, or ovate-rhombic (rounded-rhombic); with a maximum expansion a little closer to the base (1/4-1/3 of the leaf blade from the base). The leaf base is attenuate, cuneate or irregular (rounded or slightly cordate near the petiole and cuneate at a distance from it); rather smoothly passes into a convex or almost straight lateral edge [an irregular base was not mentioned in the works of other authors, but this is one of the main differences between this hybrid and many other species and hybrids]. The margin is crenate-serrate (4-6 teeth per 1 cm) (Figure, panel A). The tip is attenuate with 1-2 cm tip. The adaxial surface of the leaf is light green, and the abaxial one is whitish-greenish, but the difference in color is not very great. The midrib is relatively narrow, not very pronounced on the adaxial surface of the leaf. The glands at the petiole near the base of the leaf blade are usually absent or present only on the largest leaves, small or slightly moved away from the petiole, although sometimes there are many glands (if on half or almost all the leaves, then this may be *P.* × *nevensis* Nasim., late shedding foliage – see below). The leaves of the annual shoots are about the same or slightly larger. The leaves turn yellow in autumn and fall off earlier than in other poplars (our observations in Moscow, as well as measurements of MHA herbarium specimens). Capsules are glabrous, elongated (length on average 9 mm, width on average 5 mm), with attenuate tip, open with 2, much less often 3 valves, stalked on 1-1.5 mm long pedicels; fully opened valves twist outwards (Kostina and Nasimovich, 2012). Thirty-four samples (and 27 more samples with doubts in identification): 59 samples from fifteen points in Moscow and 2 samples from one point in the Kemerovo Region.

We also describe one hybrid with which *P.* × *sibirica* can be confused.

***Populus* × *nevensis*** Nasim. [? *P*. × *nevensis* Bogdanov, nom. inval.; (*P. deltoides* × *P. nigra*) × (*P. laurifolia* × *P. suaveolens*)] The cultivar or spontaneous hybrid from Moscow that was described by us (Kostina et al., 2017), which is close to *P.* × *sibirica* (*P. nigra* × (*P. laurifolia* × *P. suaveolens*)) and differs only in the features that *P. deltoides* adds to the three coinciding parental species: the presence of a larger number of glands at the petiole near the base of the leaf blade, on average, larger and, most importantly, slightly wider leaves, very late yellowing and flying off leaves (sometimes trees are green until snow), and less disease susceptible. Probably, it is very close to *P. × nevensis* P.L. Bogdanov, nom. inval., which was created in Leningrad (now St. Petersburg), and then lost and, perhaps, recently found again by local historians. One way or another, but good descriptions of this cultivar were not made at that time, and there are no good descriptions of new findings, and, therefore, we had to describe the corresponding accessions from Moscow as a new taxon to be able to publish information about them. *P. × nevensis* P.L. Bogdanov was described as a hybrid of *P. balsamifera* × *P.* × *canadensis* (*P. deltoides* × *P. nigra*) (Bogdanov, 1965; Yakushina, 1982). Russian botanists and breeders at the time of Bogdanov mistook *P.* × *sibirica* for *P. balsamifera* (Mayorov et al., 2012; Mayorov et al., 2020). It is also possible that under the name of *P.* × *nevensis* Nasim the following poplar was described – “Balsam poplar improved All-Union Research Institute of Agroforestry (AURIA)”, obtained by A.V. Albensky and A.V. Delitsyna from crossing of “*P. balsamifera*” and “*P. × berolinensis*” (Kotelova and Stelmakhovich, 1963), although *P.* × *sibirica* and *P.* × *petrovskoe* were meant. Then, the formal composition of “**Balsam poplar improved by AURIA**” is 37.5% *P. laurifolia*, 37.5% *P. nigra*, 12.5% *P. deltoides*, and 12.5% *P. suaveolens*, which is close to *P.* × *nevensis* in our understanding. Unfortunately, the descriptions of AURIA poplar known to us are not enough for reliable conclusions, and this cultivar also does not have a correct international name.


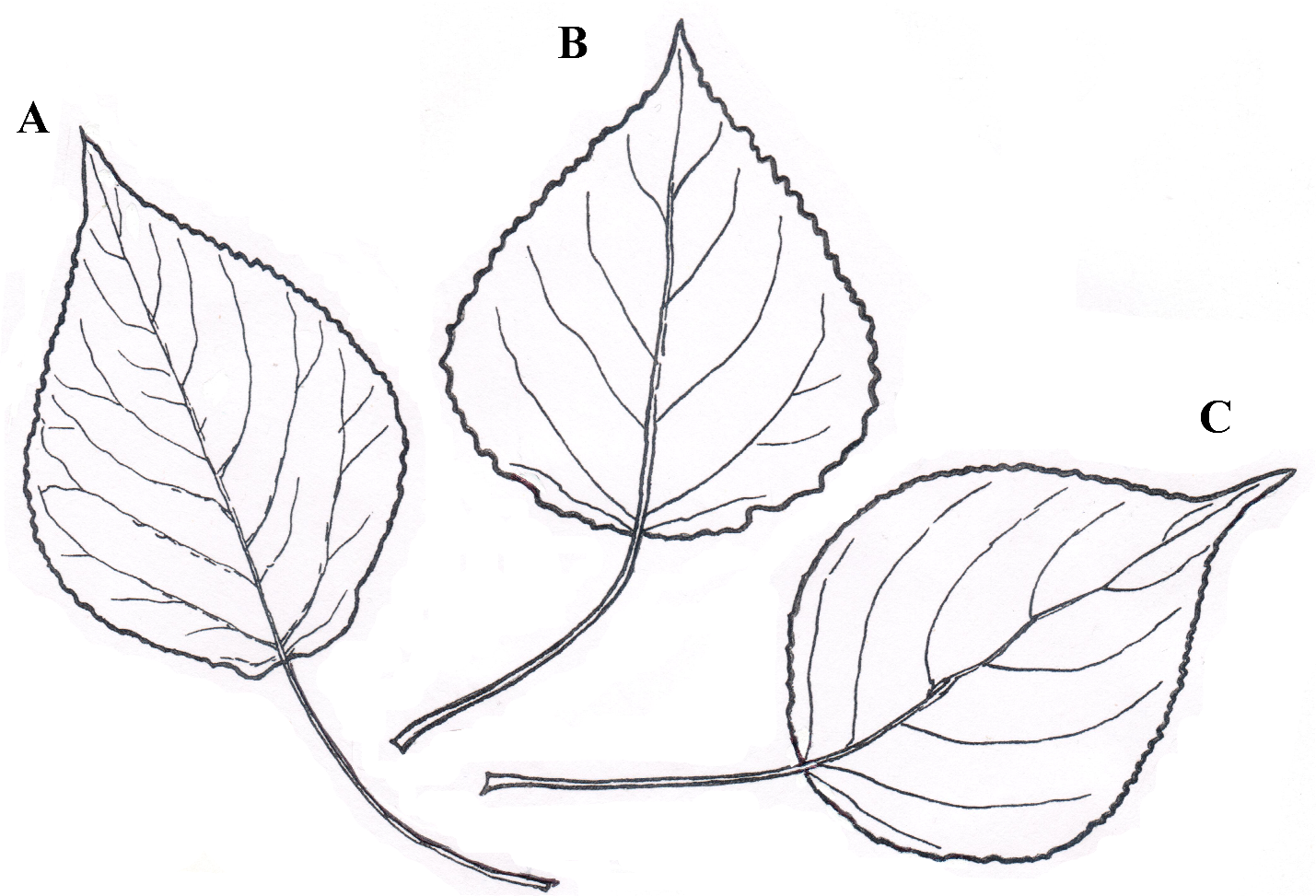


**Figure.** Leaves of *P.* × *sibirica* (A), *P.* × *petrovskoe* (B), and *P.* × *rasumovskoe* (C).

**References**

Albensky, A.V. (1954). Techniques for improving tree species. [Metody uluchsheniya drevesnyh porod]. Moscow – Leningrad: Goslestechizdat. (In Russian)

Ascherson, P., Graebner, P. (1908-1913). Synopsis der Mitteleuropaischen Flora. Leipzig: W. Engelmann.

Bogdanov, P.L. (1965). Poplars and their cultivation [Topolya i ih kul'tura]. Moscow: Forest industry. (In Russian)

Borodina, N.A., Nekrasov, V.I., Nekrasov, N.S., et al. (1966). Trees and shrubs of the USSR [Derev'ya i kustarniki SSSR], eds. P.I. Lapin. Moscow: Mysl'. (In Russian)

Broeck, A.V., Storme, V., Cottrell, J.E., Boerjan, W., Van Bockstaele, E., Quataert, P., Van Slycken, J. (2004). Gene flow between cultivated poplars and native black poplar (*Populus nigra* L.): a case study along the river Meuse on the Dutch-Belgian border. Forest Ecology and Management. 197:1, 307-310.

Brus, R., Galien, U., Božič, G., Jarni, K. (2010). Morphological study of the leaves of two European black poplar (*Populus nigra* L.) populations in Slovenia. Periodicum biologorum. 112:3, 317-325.

Bryntsev, V.A., Makhrova, T.G., Aksenov, P.A. (2019). Poplars A.S. Yablokova in green areas of the city of Moscow and settlements of the Moscow Region [Topolya selekcii A.S. Yablokova v zelenyh nasazhdeniyah Moskvy i Moskovskoj oblasti]. Forestry Information. 2, 103-110. (In Russian)

Cagelli, L., Lefevre, F. (1995). The conservation of *Populus nigra* L. and gene flow with cultivated poplars in Europe. Forest Genetics. 2:3, 135-144.

Dippel, L. (1892). Handbuch der Laubholzkunde. Berlin: Paul Parey. 190-211.

IPNI, International Plant Name Index, https://www.ipni.org/

Kachalov, A.A. (1970). Trees and shrubs [Derev'ya i kustarniki]. Moscow: Forest industry. (In Russian)

Karhu, N., Hamet-Ahti, L. (1992). Gen. *Populus*. Suomen puu-ja pensaskasvio. Helsinki: Dendrologian Seura. 142-152.

Koltzenburg, M. (1999). Bestimmungsschlussel fur in Mitteleuropa heimische und kultivierte Pappelarten und -sorten (*Populus* spec.). Floristische Rundbriefe. Göttingen: E. Goltze.

Komarov, V.L. (1936). Genus *Populus*. Flora of the USSR [Rod Topol'. Flora SSSR]. Moscow – Leningrad: Akad. Nauk SSSR. 5, 215-242. (In Russian)

Kostina, M.V., Chindyaeva, L.N., Vasilieva, N.V. (2016). Hybridization between *Populus*×*sibirica* G. Krylov et Grig. ex Skvortsov and *Populus nigra* L. in Novosibirsk [Gibridizaciya *Populus* × *sibirica* G. Krylov et Grig. ex Skvortsov i *Populus nigra* L. v Novosibirske]. Environment and Human: Ecological Studies. 4, 20-31. (In Russian)

Kostina, M.V., Nasimovich, Yu.A. (2012). To the taxonomy of poplars growing in urban plantings of Moscow and the Moscow Region [K sistematike topolej, proizrastayushchih v gorodskih posadkah Moskvy i Moskovskoj oblasti]. Biodiversity: problems of study and conservation: materials of the Intern. scientific conf., dedicated 95th anniversary of the department. botany of the Tver state. University (Tver, November 21-24, 2012). Tver: Tver. state un-t. 189-192. (In Russian)

Kostina, M.V., Nasimovich, Yu.A. (2018). Cultivated poplars (*Populus*, Salicaceae) of Saratov and Engels (Saratov province) [Kul'tiviruemye topolya (*Populus*, Salicaceae) Saratova i Engel'sa (Vologodskaya oblast')]. Environment and Human: Ecological Studies. 3, 33-42. (In Russian)

Kostina, M.V., Nasimovich, Yu.A. (2014). On the systematics of *Populus* L. II. Importance of fruit characters for identification of cultivated and adventive species in Moscow Region [K sistematike roda *Populus* L. II. Znachenie priznakov korobochek dlya opredeleniya sistematicheskogo statusa topolej, kul'tiviruemyh i dichayushchih v Moskovskom regione]. Bulletin of Moscow Society of Naturalists. Biological series. 119:5, 74-79. (In Russian)

Kostina, M.V., Vasilieva, N.V., Nasimovich, Yu.A. (2018). Natural and cultivated poplars of Irkutsk Province and Buryat Republic [Prirodnye i kul'tiviruemye topolya Irkutskoj oblasti i Buryatii]. Environment and Human: Ecological Studies. 3, 9-21. (In Russian)

Kotelova, N.V., Stelmakhovich, M.L. (1963). Poplars and their use in green areas [Topolya i ih ispol'zovanie v zelyonyh nasazhdeniyah]. Moscow: Sel'hozizdat. (In Russian)

Krylov, G.V. 1961. Forests of Western Siberia. History of study, types of forests, zoning, ways of use and improvement [Lesa Zapadnoj Sibiri. Istoriya izucheniya, tipy lesov, rajonirovanie, puti ispol'zovaniya i uluchsheniya]. Moscow: Akad. Nauk SSSR. (In Russian)

Mayorov, S.R., Alekseev, Yu.E., Bochkin, V.D., Nasimovich, Yu.A., Shcherbakov, A.V. (2020). Alien flora of the Moscow Region: the composition, origin and the vectors of formation [Chuzherodnaya flora Moskovskogo regiona: sostav, proiskhozhdenie i puti formirovaniya]. Moscow: KMK. (In Russian)

Mayorov, S.R., Bochkin, V.D., Nasimovich, Yu.A., Shcherbakov, A.V. (2012). Adventive flora of Moscow and Moscow Region [Adventivnaya flora Moskvy i Moskovskoi oblasti]. Moscow: KMK. (In Russian)

Nasimovich, Yu.A., Kostina, M.V., Vasilieva, N.V. (2019). The concept of species in poplars (genus *Populus* L., Salicaceae) based on the example of the subgenus *Tacamahaca* (Spach) Penjkovsky representatives growing in Russia and neighbouring countries [Koncepciya vida u topolej (genus *Populus* L., Salicaceae) na primere predstavitelej podroda *Tacamahaca* (Spach) Penjkovsky]. Environment and Human: Ecological Studies. 9:4, 426-466. (In Russian)

Nasimovich, Yu.A., Vasilieva, N.V. (2019). Comparison of morphological characters of different poplar species (*Populus*, Salicaceae) using the example of Russian and Central Asian *Tacamahaca* (Spach) Penjkovsky subgenus representatives [Sravnenie po morfologicheskim priznakam raznyh vidov topolej (*Populus*, Salicaceae) na primere rossijskih i sredneaziatskih predstavitelej podroda *Tacamahaca* (Spach) Penjkovsky]. Environment and Human: Ecological Studies. 9:3, 285-301. (In Russian)

Proshkin, B.V., Klimov, A.V. (2017). Spontaneous hybridization of *Populus* *× sibirica* and *Populus nigra* in the city of Novokuznetsk (Kemerovo Region) [Spontannaya gibridizaciya *Populus* × *sibirica* i *Populus nigra* v gorode Novokuznecke (Kemerovskaya oblast')]. Turczaninowia. 20:4, 206-218. (In Russian)

Recommendations on the range of introduced species and hybrids of tree species for forest crops and landscaping in the RSFSR [Rekomendacii po assortimentu introducentov i gibridov drevesnyh porod dlya lesnyh kul'tur i ozeleneniya v RSFSR]. (1976). Moscow: VNIILM. (In Russian)

Redko, G.V. (1975). Biology and cultivation of poplars [Biologiya i kul'tura topolej]. Leningrad: Leningrad. Univ. press. (In Russian)

Regel, E. (1889). Russian dendrology [Russkaya dendrologiya]. Issue 2. St. Petersburg: Izdanie Karla Rikkera. (In Russian)

Rehder, A. (1949). Manual of cultivated trees and shrubs. New York: MacMillan.

Reshetnikova, N.M., Mayorov, S.R., Skvortsov, A.K., Krylov, A.V., Voronkina, N.V., Popchenko, M.I., Shmytov, L.L. (2010). Flora of Kaluga: annotated list of vascular plants of the Kaluga Region [Kaluzhskaya flora: annotirovannyj spisok sosudistyh rastenij Kaluzhskoj oblasti]. Moscow: KMK. (In Russian)

Skvortsov, A.K. (2007). About the Siberian Balsamic Poplar [O sibirskom "bal'zamicheskom" topole]. Bulletin of the Main Botanical Garden. 193, 41-45. (In Russian)

Skvortsov, A.K. (2010). Systematic synopsis of the genus *Populus* in Eastern Europe, Northern and Central Asia [Sistematicheskij konspekt roda *Populus* v vostochnoj Evrope, Severnoj i Srednej Azii]. Bulletin of the Main Botanical Garden. 196, 62-73. (In Russian)

Skvortsov, A.K. (2008). About some poplars described by F.B. Fischer in 1841 [O nekotoryh topolyah, opisannyh F.B. Fisherom v 1841 g]. Bulletin of the Main Botanical Garden. 194, 61-67. (In Russian)

Skvortsov, A.K., Belyanina, N.B. (2006). About balsam poplars (*Populus*, section *Tacamahaca*, Salicaceae) in the east of Asian Russia [O bal'zamicheskih topolyah (*Populus*, section *Tacamahaca*, Salicaceae) na vostoke aziatskoj Rossii]. Botanicheskii Zhurnal. 91:8, 1244-1252. (In Russian)

Sokolov, S.Ya., Shipchinsky, N.V., Yarmolenko, A.V. (1951). Genus *Populus*. Trees and shrubs of the USSR [Rod Topol'. Derev'ya i kustarniki SSSR]. Moscow – Leningrad: Akad. Nauk SSSR. 2, 174-217. (In Russian)

Syreyshchikov, D.P. (1907). Illustrated flora of the Moscow province [Illyustrirovannaya flora Moskovskoj gubernii]. Part 2. Moscow. (In Russian)

Tsinovskis, R. (1977). Two rare half-forgotten species of the genus Poplar (*Populus* L.) from the north-west of North America and related species and hybrids in Latvia [Dva redkih poluzabytyh vida roda topol' (*Populus* L.) s severo-zapada Severnoj Ameriki i blizkie im vidy i gibridy v Latvii]. Botanicheskie sady Pribaltiki. Okhrana rastenii. Riga: Zinatne. 175-196. (In Russian)

Tsvelev, N.N. (2001). About poplars (*Populus*, Salicaceae) of St. Petersburg and Leningrad Region [O topolyah (*Populus*, Salicaceae) Sankt-Peterburga i Leningradskoj oblasti]. Botanicheskii Zhurnal. 86:2, 70-78. (In Russian)

Wolkenstein M. [P.W.] (1882). New Plants at the Moscow Exhibition. The gardeners' chronicle. A weekly Illustrated Journal of Horticulture and Allied Subjects. XVIII. New series. London: Bradbury, Agnew, and Co. 108.

Yakushina, E.I. (1982). Woody plants in the landscaping of Moscow [Drevesnye rasteniya v ozelenenii Moskvy]. Moscow: Nauka. (In Russian)
