# Supplementary material for: Sex-determining region complements traditionally used in phylogenetic studies nuclear and chloroplast sequences in investigation of Aigeiros Duby and Tacamahaca Spach poplars (genus Populus L., Salicaceae)
Source: Front Plant Sci. 2023 Oct 4;14:1204899. doi: 10.3389/fpls.2023.1204899 (PMC10582643; doi:10.3389/fpls.2023.1204899)
Supplement: Supplementary file 3 [file DataSheet_3.docx]

**Supplementary Data 3.** Table of primers used in the study and Figure showing the localization of the obtained amplicons in the sex-determining region (SDR) and the *ARR17* gene of poplars of sections *Aigeiros* and *Tacamahaca*.

**Table. Sequences of primers for the preparation of DNA libraries for targeted deep sequencing on the Illumina platform.**

| **Primers** | **Sequences** | **References to the genome-specific parts (marked in regular font) of primers** |
| --- | --- | --- |
| **Primers for the amplification of nuclear and chloroplast genome loci previously used in poplar phylogenetic studies (NTS 5S rDNA, ITS, *DSH 2*, *DSH 5*, *DSH 8*, *DSH 12*, *DSH 29*, *6*, *15*, *16*, *X18*, *trnG‐psbK-psbI*, *rps2‐rpoC2*, and *rpoC2-rpoC1*)** | | |
|  |  |  |
| 5S1 F | **TCGTCGGCAGCGTCAGATGTGTATAAGAGACAG**GGATGGGTGACCTCCCGGGAAGTCC | Falistocco et al.,2007 |
| 5S2 R | **GTCTCGTGGGCTCGGAGATGTGTATAAGAGACAG**CGCTTAACTGCGGAGTTCTGATGGG | Falistocco et al.,2007 |
|  |  |  |
| ITS5 Illu F | **TCGTCGGCAGCGTCAGATGTGTATAAGAGACAG**TCGTAACAAGGTTTCCGTAGGTG | Hsiao et al., 1995 |
| ITS4 Illu R | **GTCTCGTGGGCTCGGAGATGTGTATAAGAGACAG**TCCTCCGCTTATTGATATGC | Hsiao et al., 1995 |
|  |  |  |
| DSH 2 F | **TCGTCGGCAGCGTCAGATGTGTATAAGAGACAG**CATCTTTTGCCTTATTGTCTGCT | Du et al., 2014 |
| DSH 2 R | **GTCTCGTGGGCTCGGAGATGTGTATAAGAGACAG**TGCGTTAAATGATCTTTCTGGTA | Du et al., 2014 |
|  |  |  |
| DSH 5 F | **TCGTCGGCAGCGTCAGATGTGTATAAGAGACAG**TGGCAGAATCACCAGACCCTC | Du et al., 2014 |
| DSH 5 R | **GTCTCGTGGGCTCGGAGATGTGTATAAGAGACAG**CCAATTTAGCATCTTCAGCCTCAT | Du et al., 2014 |
|  |  |  |
| DSH 8 F | **TCGTCGGCAGCGTCAGATGTGTATAAGAGACAG**GTTTGTTGTTCTGTTGATTGT | Du et al., 2014 |
| DSH 8 R | **GTCTCGTGGGCTCGGAGATGTGTATAAGAGACAG**GGCTTCTCTTCTCTGATATTT | Du et al., 2014 |
|  |  |  |
| DSH 12 F | **TCGTCGGCAGCGTCAGATGTGTATAAGAGACAG**CACCACATCCCGCTTTCTCTCTTCACTT | Du et al., 2014 |
| DSH 12 R | **GTCTCGTGGGCTCGGAGATGTGTATAAGAGACAG**TAAACCCCAGGAGGCAAAACAGCACCAG | Du et al., 2014 |
|  |  |  |
| DSH 29 F | **TCGTCGGCAGCGTCAGATGTGTATAAGAGACAG**GTATCCAATGCCAATCAC | Wang et al., 2014 |
| DSH 29 R | **GTCTCGTGGGCTCGGAGATGTGTATAAGAGACAG**AACAAGGAACAGCACCAC | Wang et al., 2014 |
|  |  |  |
| 6 F | **TCGTCGGCAGCGTCAGATGTGTATAAGAGACAG**TCTGCTTTCCACTTCTTGC | Wang et al., 2019 |
| 6 R | **GTCTCGTGGGCTCGGAGATGTGTATAAGAGACAG**CATACTCTCCCATTGTCCC | Wang et al., 2019 |
|  |  |  |
| 15 F | **TCGTCGGCAGCGTCAGATGTGTATAAGAGACAG**GCCTCCTGATTATTATGC | Wang et al., 2019 |
| 15 R | **GTCTCGTGGGCTCGGAGATGTGTATAAGAGACAG**TATTACAAGCCCTTCCAG | Wang et al., 2019 |
|  |  |  |
| 16 F | **TCGTCGGCAGCGTCAGATGTGTATAAGAGACAG**GGTAGCGTATTCAAAGATGGCAGAGG | Wang et al., 2019 |
| 16 R | **GTCTCGTGGGCTCGGAGATGTGTATAAGAGACAG**TGGACGGACCAAGAAAAACGGAGGAT | Wang et al., 2019 |
|  |  |  |
| X18 F | **TCGTCGGCAGCGTCAGATGTGTATAAGAGACAG**TATGGAAAAAGTTATGCCAAGAGGA | Wang et al., 2019 |
| X18 R | **GTCTCGTGGGCTCGGAGATGTGTATAAGAGACAG**CAAAGGAGCAGAAGGCTATATCAAG | Wang et al., 2019 |
|  |  |  |
| trnG-psbK F | **TCGTCGGCAGCGTCAGATGTGTATAAGAGACAG**gaaggattcgaacctccgaatg | Schroeder et al., 2012 |
| trnG-psbK R | **GTCTCGTGGGCTCGGAGATGTGTATAAGAGACAG**ctggcataacatctacgattgg | Schroeder et al., 2012 |
|  |  |  |
| psbK-psbI F | **TCGTCGGCAGCGTCAGATGTGTATAAGAGACAG**ccaatcgtagatgttatgccag | Schroeder et al., 2012 |
| psbK-psbI R | **GTCTCGTGGGCTCGGAGATGTGTATAAGAGACAG**ggattacgccctggatcattag | Schroeder et al., 2012 |
|  |  |  |
| rps2-rpoC2 F | **TCGTCGGCAGCGTCAGATGTGTATAAGAGACAG**ccttacgctttgcagagatataag | Schroeder et al., 2012 |
| rps2-rpoC2 R | **GTCTCGTGGGCTCGGAGATGTGTATAAGAGACAG**gagagatattttgtttcaccacag | Schroeder et al., 2012 |
|  |  |  |
| rpoC2-rpoC1 F | **TCGTCGGCAGCGTCAGATGTGTATAAGAGACAG**gcagtggcttgatggaaacccag | Schroeder et al., 2012 |
| rpoC2-rpoC1 R | **GTCTCGTGGGCTCGGAGATGTGTATAAGAGACAG**gagaagctcccatcgaagctgac | Schroeder et al., 2012 |
|  |  |  |
| **Primers for the amplification of the SDR and *ARR17* gene loci** | | |
|  |  |  |
| N R7 2 F | **TCGTCGGCAGCGTCAGATGTGTATAAGAGACAG**CATGGAGATCTCTTGAAGAAAGGG | present study |
| N R7 2 R | **GTCTCGTGGGCTCGGAGATGTGTATAAGAGACAG**ATATCTACCTTCAAAAAACACACCAC | present study |
|  |  |  |
| N R7 1 F | **TCGTCGGCAGCGTCAGATGTGTATAAGAGACAG**TTGATTTTGATGAGAAACCACATGTG | present study |
| N R7 1 R | **GTCTCGTGGGCTCGGAGATGTGTATAAGAGACAG**GTGTTCTATGTTTTTTCCCTTTTGCA | present study |
|  |  |  |
| N R3 2 F | **TCGTCGGCAGCGTCAGATGTGTATAAGAGACAG**TTTGATGATTGGATTATGAATGGTT | present study |
| N R3 2 R | **GTCTCGTGGGCTCGGAGATGTGTATAAGAGACAG**ATAACAAGGTCACACAGCCAT | present study |
|  |  |  |
| R3 6 F | **TCGTCGGCAGCGTCAGATGTGTATAAGAGACAG**AGGTATGAGGAAGAATAAAAAAGGGA | present study |
| R3 6 R | **GTCTCGTGGGCTCGGAGATGTGTATAAGAGACAG**TCCTTCCAAAACCATCACACCTA | present study |
|  |  |  |
| N R3 1 F | **TCGTCGGCAGCGTCAGATGTGTATAAGAGACAG**AGGAAATCTGAGACACAGTAGTGG | present study |
| N R3 1 R | **GTCTCGTGGGCTCGGAGATGTGTATAAGAGACAG** AACCATTCATAATCCAATCATCAAA | present study |
|  |  |  |
| 42 604 1028 F | **TCGTCGGCAGCGTCAGATGTGTATAAGAGACAG**TAAGTGGTCGTGGTCAAGGC | present study |
| 42 604 1028 R | **GTCTCGTGGGCTCGGAGATGTGTATAAGAGACAG**TCCATCACCAAATTCATTCTTCCA | present study |
|  |  |  |
| 42 1097 1507 F | **TCGTCGGCAGCGTCAGATGTGTATAAGAGACAG**CCCCATGTTTTTCACCAACCC | present study |
| 42 1097 1507 R | **GTCTCGTGGGCTCGGAGATGTGTATAAGAGACAG**GGCACARTACCAAGCCCAAA | present study |
|  |  |  |
| 42 4250 4648 F | **TCGTCGGCAGCGTCAGATGTGTATAAGAGACAG**TTGCCGATGCTTGTGTTGAA | present study |
| 42 4250 4648 R | **GTCTCGTGGGCTCGGAGATGTGTATAAGAGACAG**TGGAAGATTGGATTGGGAAGGG | present study |
|  |  |  |
| 19 455 1035 F | **TCGTCGGCAGCGTCAGATGTGTATAAGAGACAG**TCAACATCATCCATTRCTTTAACA | present study |
| 19 455 1035 R | **GTCTCGTGGGCTCGGAGATGTGTATAAGAGACAG**GGCTTCRGATATGCAWTCCA | present study |
|  |  |  |
| 19 978 1611 F | **TCGTCGGCAGCGTCAGATGTGTATAAGAGACAG**TCYAAARAARGGGTAAAAGAAA | present study |
| 19 978 1611 R | **GTCTCGTGGGCTCGGAGATGTGTATAAGAGACAG**CCTTCAAAGAACACACCACA | present study |
|  |  |  |
| 19 1592 2304 F | **TCGTCGGCAGCGTCAGATGTGTATAAGAGACAG**TGTGGTGTGTTCTTTGAAGGT | present study |
| 19 1592 2304 R | **GTCTCGTGGGCTCGGAGATGTGTATAAGAGACAG**GACADGATATTCTTTTYCTTTWYA | present study |
|  |  |  |
| 19 1740 2406 F | **TCGTCGGCAGCGTCAGATGTGTATAAGAGACAG**AYATGYAGAGGTATGAAMAGAAC | present study |
| 19 1740 2406 R | **GTCTCGTGGGCTCGGAGATGTGTATAAGAGACAG**TGRATTTKCAATGTTMTGAGTTAT | present study |
|  |  |  |
| 19 1740 2406 2 F | **TCGTCGGCAGCGTCAGATGTGTATAAGAGACAG**AYATGYAGAGGTATGAAMAGAAC | present study |
| 19 1740 2406 2 R | **GTCTCGTGGGCTCGGAGATGTGTATAAGAGACAG**TGRATTTKCAATGTTMTAAGTTAT | present study |
|  |  |  |
| 19 2282 2729 F | **TCGTCGGCAGCGTCAGATGTGTATAAGAGACAG**RWAAAGRAAAAGAATATCHTGTCA | present study |
| 19 2282 2729 R | **GTCTCGTGGGCTCGGAGATGTGTATAAGAGACAG**ACYTGTCATTCYTGGCRTACT | present study |
|  |  |  |
| 19 2282 2792 F | **TCGTCGGCAGCGTCAGATGTGTATAAGAGACAG**GAAAAGGAAAAGAATATCCTGTCA | present study |
| 19 2282 2792 R | **GTCTCGTGGGCTCGGAGATGTGTATAAGAGACAG**ATGGTARTAACTARATCATTGAAG | present study |
|  |  |  |
| 19 2578 3125 F | **TCGTCGGCAGCGTCAGATGTGTATAAGAGACAG**CCCAGYCATAGTGTAAGTAACC | present study |
| 19 2578 3125 R | **GTCTCGTGGGCTCGGAGATGTGTATAAGAGACAG**TGTARGTGHTTRTCATTAKAYAT | present study |
|  |  |  |
| 19 3011 3548 F | **TCGTCGGCAGCGTCAGATGTGTATAAGAGACAG**TCAARCGRTAACYTAYAAYTTTCA | present study |
| 19 3011 3548 R | **GTCTCGTGGGCTCGGAGATGTGTATAAGAGACAG**ACTACAACCACCGGTATCTCC | present study |
|  |  |  |
| 19 3494 3973 F | **TCGTCGGCAGCGTCAGATGTGTATAAGAGACAG**TGGTTTCTCTCAAATAGGAATCACC | present study |
| 19 3494 3973 R | **GTCTCGTGGGCTCGGAGATGTGTATAAGAGACAG**AAAGGGATGAAACAGTGAAATG | present study |
|  |  |  |
| 19 3909 4407 F | **TCGTCGGCAGCGTCAGATGTGTATAAGAGACAG**GCTACTGCAATCAATAGAAGTTGTT | present study |
| 19 3909 4407 R | **GTCTCGTGGGCTCGGAGATGTGTATAAGAGACAG**RTAACATCTGCTTCGYAATGTTC | present study |
|  |  |  |
| 19 4050 4592 F | **TCGTCGGCAGCGTCAGATGTGTATAAGAGACAG**GGCATTTGCAGATAATTTCTTGT | present study |
| 19 4050 4592 R | **GTCTCGTGGGCTCGGAGATGTGTATAAGAGACAG**GGCAATTTCCCCTGCAAAATCT | present study |
|  |  |  |
| 19 4050 4681 F | **TCGTCGGCAGCGTCAGATGTGTATAAGAGACAG**GGCATTTGCAGATAATTTCTTGT | present study |
| 19 4050 4681 R | **GTCTCGTGGGCTCGGAGATGTGTATAAGAGACAG**ARAAATCACCTCAACCTTATTCA | present study |
|  |  |  |
| 19 5533 6115 F | **TCGTCGGCAGCGTCAGATGTGTATAAGAGACAG**TRYATC AAADCATTRTTCATA A | present study |
| 19 5533 6115 R | **GTCTCGTGGGCTCGGAGATGTGTATAAGAGACAG**ATATG TCTRCTTWGTYAAAATAAT | present study |
|  |  |  |
| 19 4569 5092 F | **TCGTCGGCAGCGTCAGATGTGTATAAGAGACAG**GGAGATTTTGCAGRGGAAATTG | present study |
| 19 4569 5092 R | **GTCTCGTGGGCTCGGAGATGTGTATAAGAGACAG**TRGACACRTKRGCCTCTCTA | present study |
|  |  |  |
| 19 4760 5329 F | **TCGTCGGCAGCGTCAGATGTGTATAAGAGACAG**CGAGTCATTTGCTCACCCAT | present study |
| 19 4760 5329 R | **GTCTCGTGGGCTCGGAGATGTGTATAAGAGACAG**TTTTWAAATTGCTTTTKACKTTTTT | present study |
|  |  |  |
| 19 5272 5735 F | **TCGTCGGCAGCGTCAGATGTGTATAAGAGACAG**GCAAKWTATAAATGATAYATGCAA | present study |
| 19 5272 5735 R | **GTCTCGTGGGCTCGGAGATGTGTATAAGAGACAG**MTYTTGCAGRTTAGTTMAGTGT | present study |
|  |  |  |
| 19 6007 6565 F | **TCGTCGGCAGCGTCAGATGTGTATAAGAGACAG**AAATCYTTAAYCGATGTGGAA | present study |
| 19 6007 6565 R | **GTCTCGTGGGCTCGGAGATGTGTATAAGAGACAG**ATRTACTATTATAAGARSCATCACA | present study |
|  |  |  |
| 19 5714 6030 F | **TCGTCGGCAGCGTCAGATGTGTATAAGAGACAG**ACACTK AACTAACYTGCAARA | present study |
| 19 5714 6030 R | **GTCTCGTGGGCTCGGAGATGTGTATAAGAGACAG**TTATT CCACATCGRTTAARGA | present study |
|  |  |  |
| 14 819 1245 F | **TCGTCGGCAGCGTCAGATGTGTATAAGAGACAG**AGAAGTGCTCTTCRTATCTTCAAT | present study |
| 14 819 1245 R | **GTCTCGTGGGCTCGGAGATGTGTATAAGAGACAG**TCTTRGAATGGAGRTCAAGCA | present study |
|  |  |  |
| 14 3096 3614 F | **TCGTCGGCAGCGTCAGATGTGTATAAGAGACAG**TCMYTTTTCTWTTAGCWRACCC | present study |
| 14 3096 3614 R | **GTCTCGTGGGCTCGGAGATGTGTATAAGAGACAG**AGYAATGAAAACACTRHCARC | present study |
|  |  |  |
| 9 962 1489 F | **TCGTCGGCAGCGTCAGATGTGTATAAGAGACAG**GCRCRCACATTATCCAATTTCA | present study |
| 9 962 1489 R | **GTCTCGTGGGCTCGGAGATGTGTATAAGAGACAG**CCAAACAAAGACCACCGCAA | present study |
|  |  |  |
| 4 836 1413 F | **TCGTCGGCAGCGTCAGATGTGTATAAGAGACAG**GGACAYADGAATCARTTCAGAGG | present study |
| 4 836 1413 R | **GTCTCGTGGGCTCGGAGATGTGTATAAGAGACAG**TRCAYTMGTCTGGACCTCCT | present study |
|  |  |  |
| Ferr 1 F | **TCGTCGGCAGCGTCAGATGTGTATAAGAGACAG**GGTYATCCRKGTTGCATTTGG | present study |
| Ferr 1 R | **GTCTCGTGGGCTCGGAGATGTGTATAAGAGACAG**CAAGGACATTMAGTGCAAGGGTTATG | present study |
|  |  |  |
| 19 2282 2729 F | **TCGTCGGCAGCGTCAGATGTGTATAAGAGACAG**RWAAAGRAAAAGAATATCHTGTCA | present study |
| Ferr 2 R | **GTCTCGTGGGCTCGGAGATGTGTATAAGAGACAG**CtatgRctgggatgttgtccatca | present study |
|  |  |  |
| **Primers for the second PCR** | | |
|  |  |  |
| Nextera XT v2 (i7) | CAAGCAGAAGACGGCATACGAGAT[i7]**GTCTCGTGGGCTCGG** | Illumina universal primers |
| Nextera XT v2 (i5) | AATGATACGGCGACCACCGAGATCTACAC[i5]**TCGTCGGCAGCGTC** | Illumina universal primers |

*Note:* Illumina overhang adapter sequences are in bold. Sequences of the genome-specific parts of primers are marked in regular font. Nextera XT v2 (i7) and Nextera XT v2 (i5) are Nextera XT v2 Illumina universal primers.


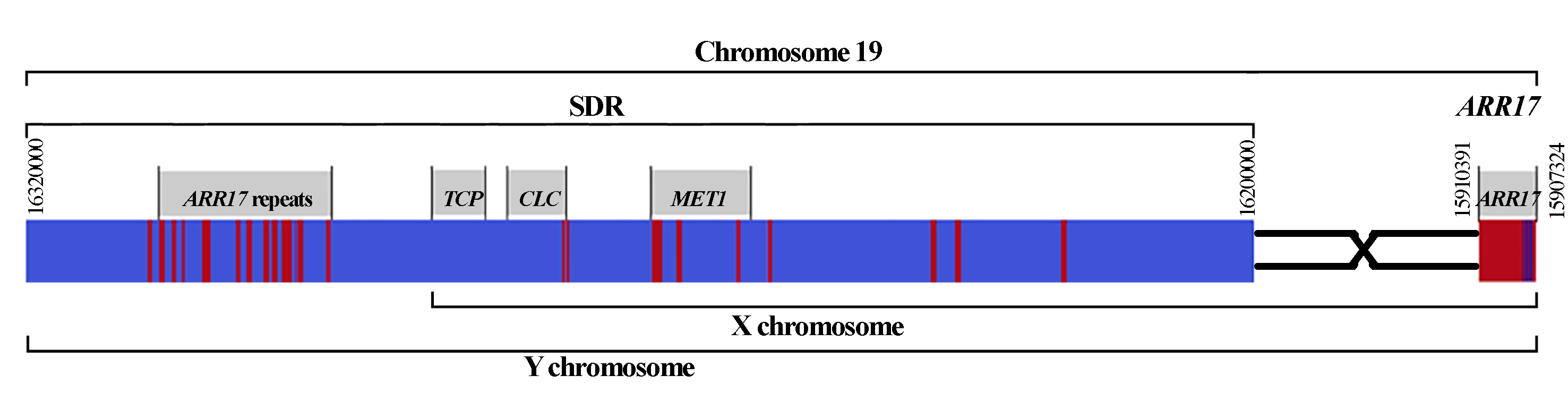


**Figure. Location of the amplified fragments in the SDR and *ARR17* gene of poplars of sections *Aigeiros* and *Tacamahaca*.** Red color corresponds to the regions that were amplified using the designed primers; blue color corresponds to the areas that we did not amplify and did not analyze. The coordinates are given according to the *P. trichocarpa* “Stettler 14” genome v1.1 (https://phytozome-next.jgi.doe.gov/info/PtrichocarpaStettler14_v1_1; the coordinates correspond to chromosome 18 for the SDR and chromosome 19 for the *ARR17* gene). It should be clarified that in the *P. trichocarpa* “Stettler 14” genome v1.1, the SDR was located on chromosome 18, that was incorrect based on the genetic marker analysis and the SDR should be placed on chromosome 19 (Zhou et al., 2020). The Y chromosome-specific region of the SDR contains partial repeats of the *ARR17* gene. The SDR region shared by the X and Y chromosomes contains *T-complex protein 1 subunit gamma* (*TCP*), *Chloride channel protein* *CLC-c* (*CLC*), and *DNA-methyltransferase 1* (*MET1*) genes, however, X and Y variants of the SDR differ significantly in DNA polymorphisms in the shared part. Location of partial repeats of the *ARR17* gene, as well as *TCP*, *CLC*, *MET1*, and *ARR17* genes is marked.

**References**

Du, S., Wang, Z., and Zhang, J. (2014). A novel set of single-copy nuclear DNA markers for the genetic study of Salicaceae. *Genetics and molecular research* 13(3), 4911-4917.

Hsiao, C., Chatterton, N.J., Asay, K.H., and Jensen, K.B. (1995). Phylogenetic relationships of the monogenomic species of the wheat tribe, Triticeae (Poaceae), inferred from nuclear rDNA (internal transcribed spacer) sequences. *Genome* 38(2), 211-223.

Falistocco, E., Passeri, V., and Marconi, G. (2007). Investigations of 5S rDNA of *Vitis vinifera* L.: sequence analysis and physical mapping. *Genome* 50(10), 927-938. doi: 10.1139/g07-070.

Schroeder, H., Hoeltken, A.M., and Fladung, M. (2012). Differentiation of *Populus* species using chloroplast single nucleotide polymorphism (SNP) markers – essential for comprehensible and reliable poplar breeding. *Plant Biol (Stuttg)* 14(2), 374-381. doi: 10.1111/j.1438-8677.2011.00502.x.

Wang, Z., Du, S., Dayanandan, S., Wang, D., Zeng, Y., and Zhang, J. (2014). Phylogeny reconstruction and hybrid analysis of *Populus* (Salicaceae) based on nucleotide sequences of multiple single-copy nuclear genes and plastid fragments. *PLoS One* 9(8), e103645. doi: 10.1371/journal.pone.0103645.

Wang, D., Wang, Z., Kang, X., and Zhang, J. (2019). Genetic analysis of admixture and hybrid patterns of *Populus hopeiensis* and *P. tomentosa*. *Sci Rep* 9(1), 4821. doi: 10.1038/s41598-019-41320-z.

Zhou, R., Macaya-Sanz, D., Schmutz, J., Jenkins, J.W., Tuskan, G.A., and DiFazio, S.P. (2020). Sequencing and Analysis of the Sex Determination Region of *Populus trichocarpa*. *Genes (Basel)* 11(8). doi: 10.3390/genes11080843.
