# Supplementary material for: Sex-determining region complements traditionally used in phylogenetic studies nuclear and chloroplast sequences in investigation of Aigeiros Duby and Tacamahaca Spach poplars (genus Populus L., Salicaceae)
Source: Front Plant Sci. 2023 Oct 4;14:1204899. doi: 10.3389/fpls.2023.1204899 (PMC10582643; doi:10.3389/fpls.2023.1204899)
Supplement: Supplementary file 6 [file DataSheet_6.docx]

**Supplementary Data 6.** Dendrograms for 379 poplar accessions of sections *Aigeiros* and *Tacamahaca* based on deep sequencing data for NTS 5S rDNA, ITS, *DSH 2*, *DSH 5*, *DSH 8*, *DSH 12*, *DSH 29*, *6*, *15*, *16*, *X18*, *trnG-psbK-psbI*, *rps2-rpoC2*, and *rpoC2-rpoC1*, as well as their combinations (**6A** – NTS 5S rDNA, ITS, *DSH 2*, *DSH 5*, *DSH 8*, *DSH 12*, *DSH 29*, *6*, *15*, *16*, *X18*, *trnG‐psbK-psbI*, *rps2‐rpoC2*, and *rpoC2-rpoC1*; **6B** – NTS 5S rDNA; **6C** – ITS; **6D** – *DSH 2*; **6E** – *DSH 8*; **6F** – *DSH 29*; **6G** – gene *6*; **6H** – gene *15*; **6I** – gene *16*; **6J** – *X18*; **6K** – *DSH 5*; **6L** – *DSH 12*; **6M** – NTS 5S rDNA, ITS, *DSH 2*, *DSH 5*, *DSH 8*, *DSH 12*, *DSH 29*, *6*, *15*, *16*, and *X18*).


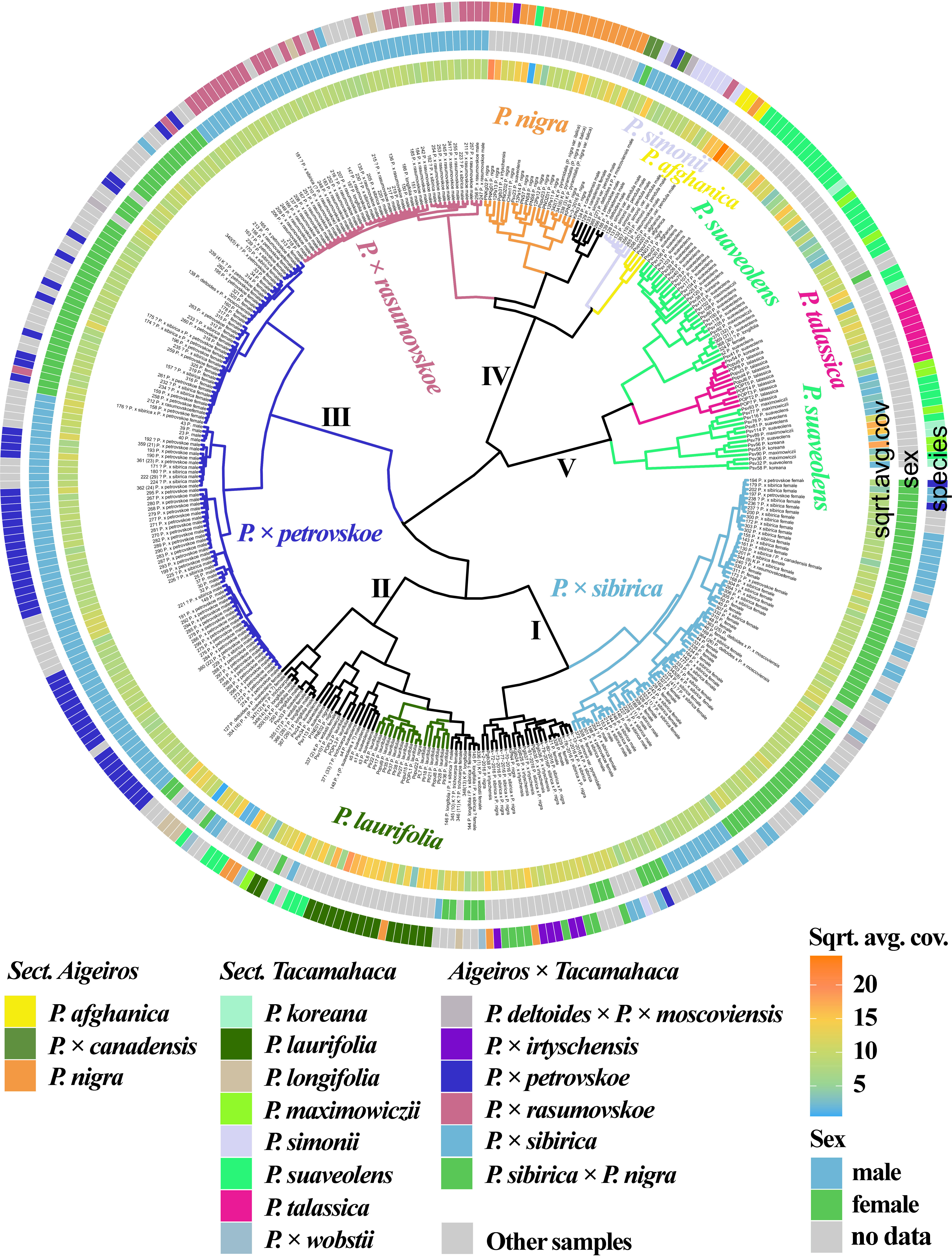


**Supplementary Data 6A.** Dendrogram based on deep sequencing data for NTS 5S rDNA, ITS, *DSH 2*, *DSH 5*, *DSH 8*, *DSH 12*, *DSH 29*, *6*, *15*, *16*, *X18*, *trnG‐psbK-psbI*, *rps2-rpoC2*, and *rpoC2-rpoC1* sequences. Colors corresponding to species and hybrids mark only accessions for which there were no doubts in the morphological determination of the species affiliation.


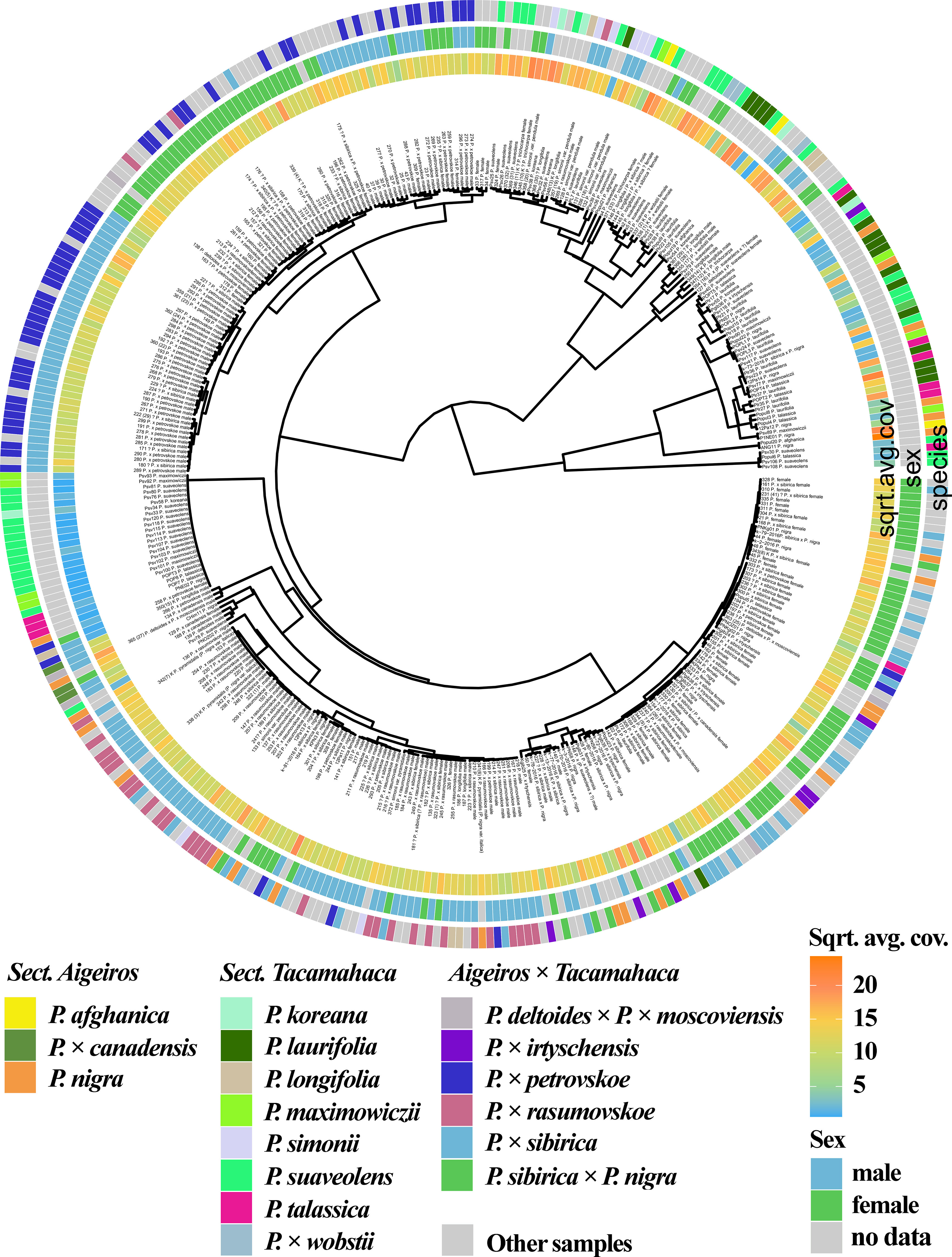


**Supplementary Data 6B.** Dendrogram based on deep sequencing data for NTS 5S rDNA sequences. Colors corresponding to species and hybrids mark only accessions for which there were no doubts in the morphological determination of the species affiliation.


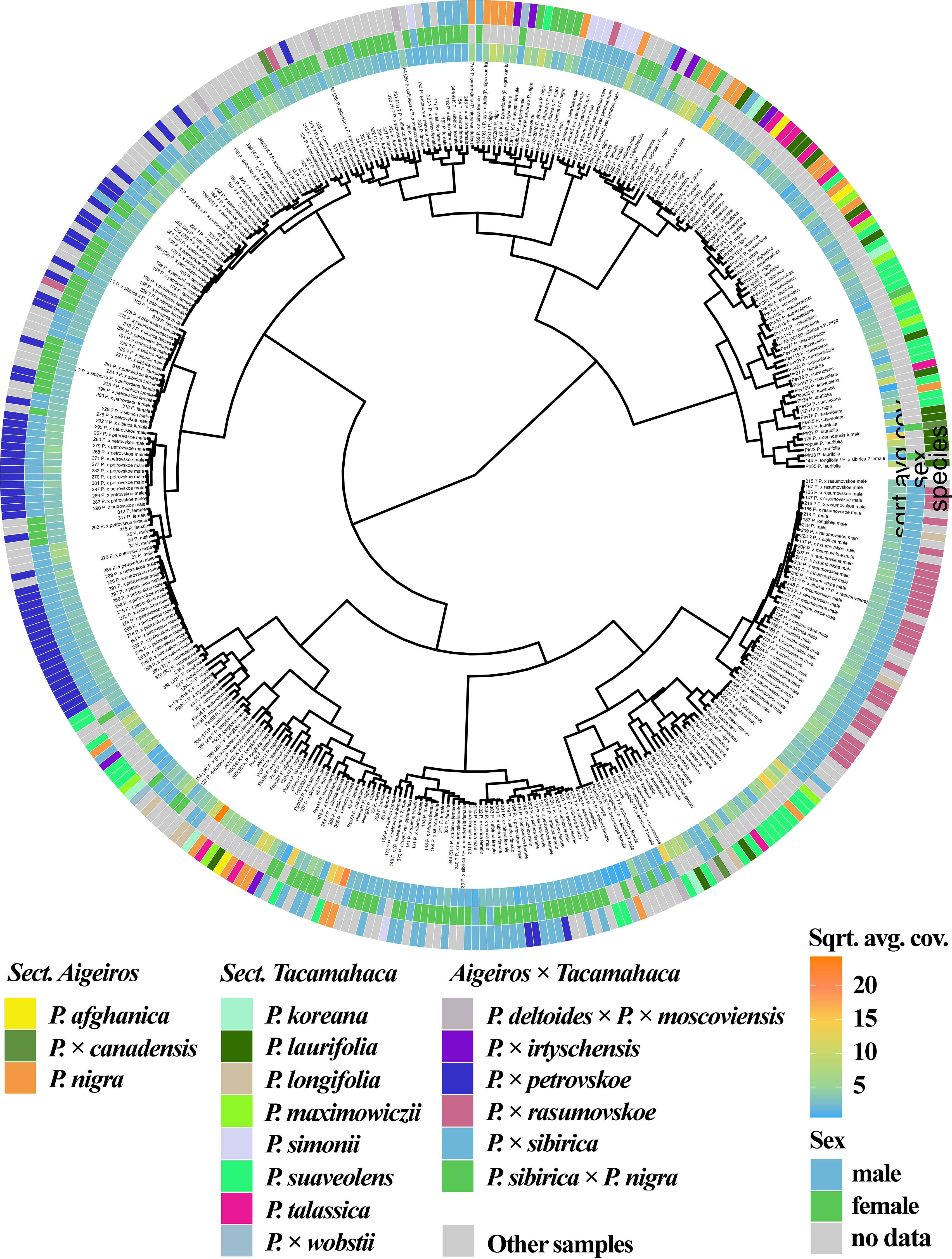


**Supplementary Data 6C.** Dendrogram based on deep sequencing data for ITS sequences. Colors corresponding to species and hybrids mark only accessions for which there were no doubts in the morphological determination of the species affiliation.


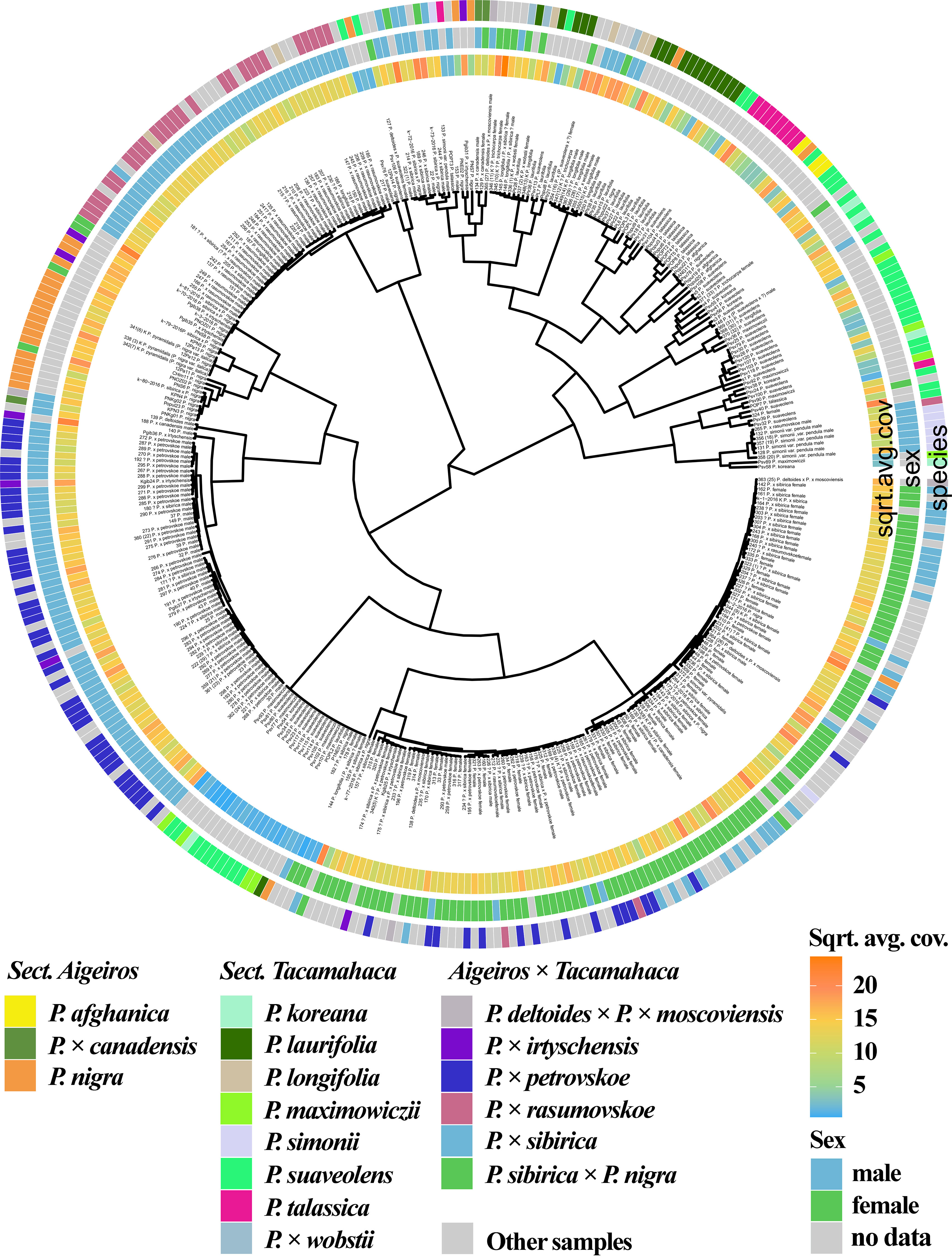


**Supplementary Data 6D.** Dendrogram based on deep sequencing data for *DSH 2* sequences. Colors corresponding to species and hybrids mark only accessions for which there were no doubts in the morphological determination of the species affiliation.


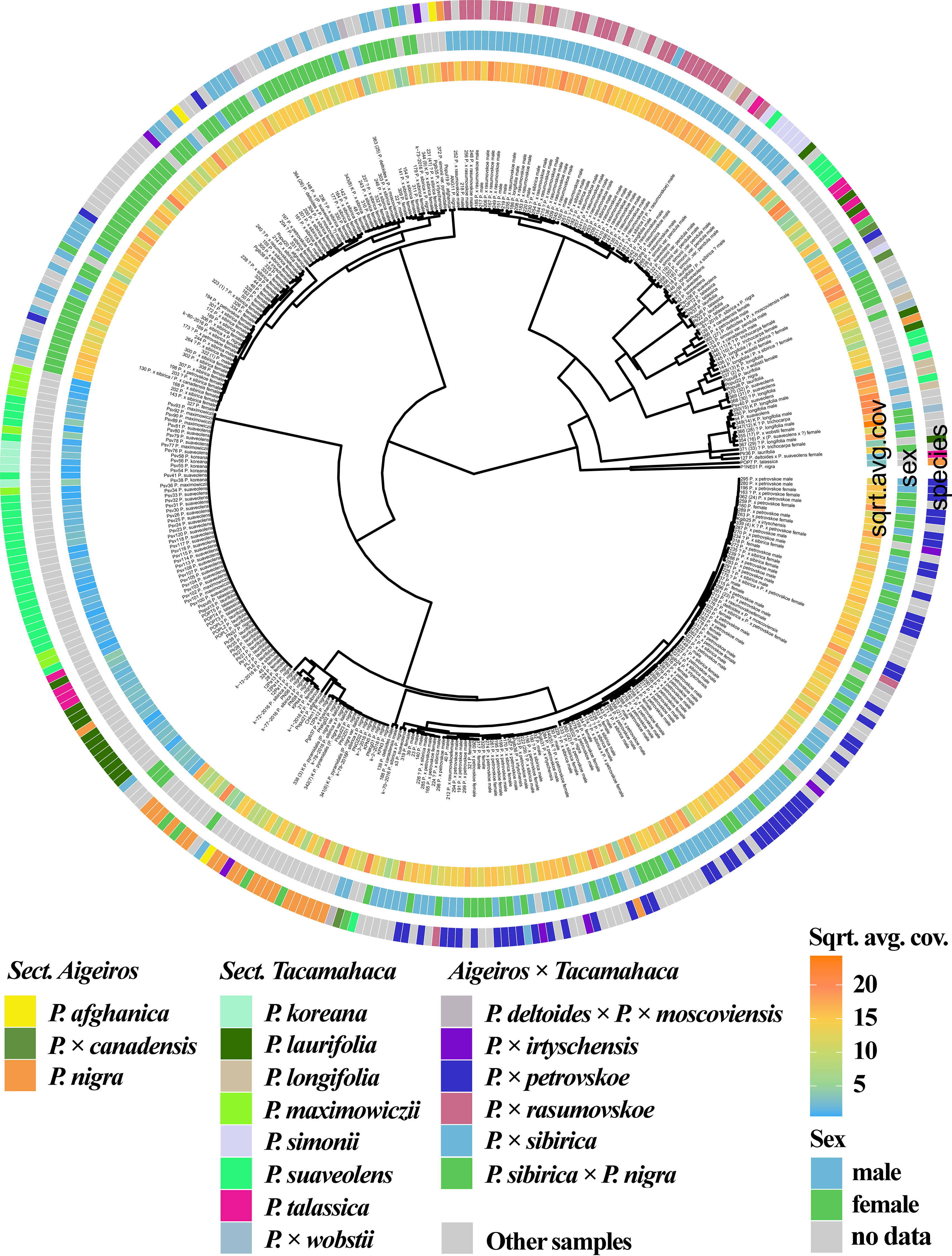


**Supplementary Data 6E.** Dendrogram based on deep sequencing data for *DSH 8* sequences. Colors corresponding to species and hybrids mark only accessions for which there were no doubts in the morphological determination of the species affiliation.


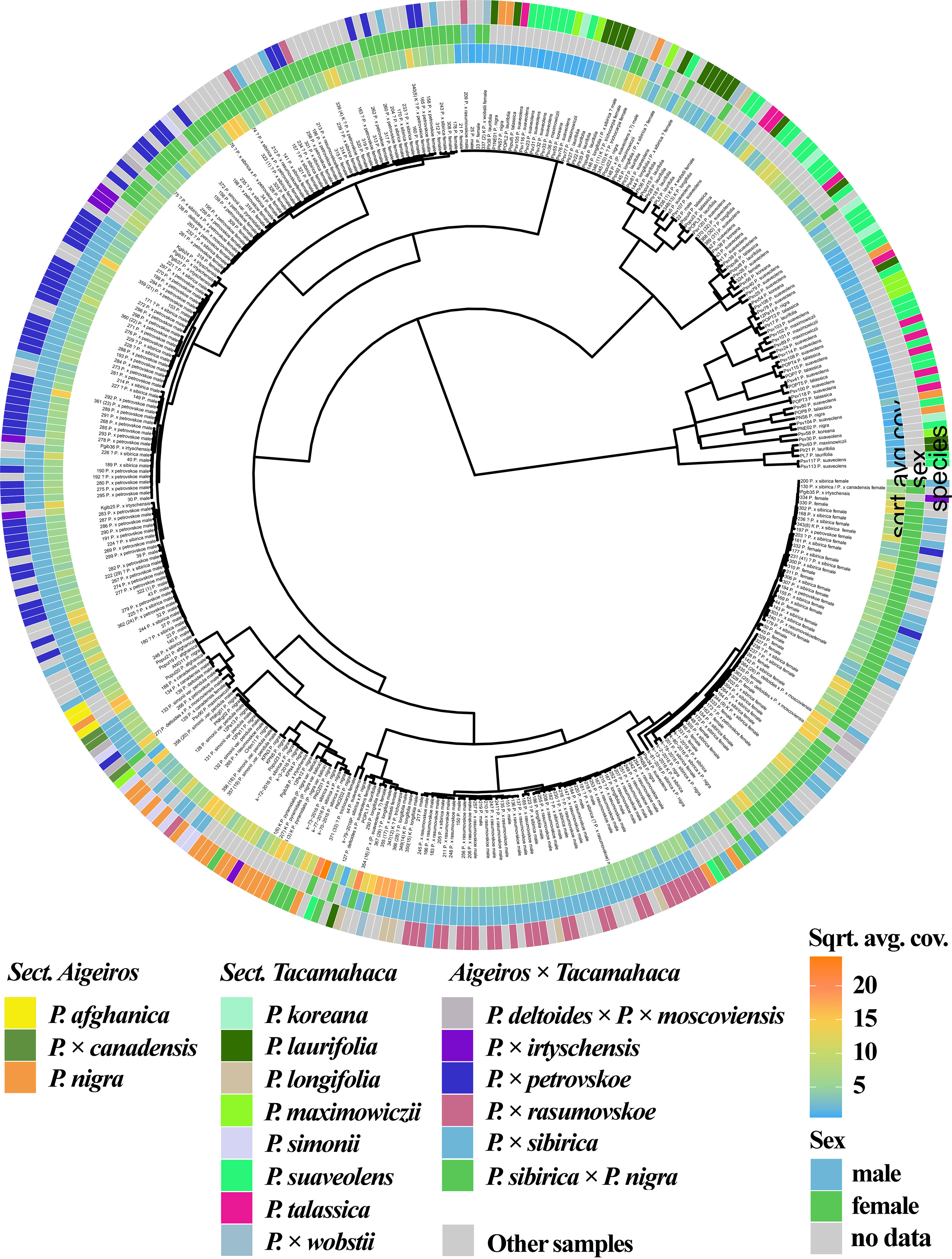


**Supplementary Data 6F.** Dendrogram based on deep sequencing data for *DSH 29* sequences. Colors corresponding to species and hybrids mark only accessions for which there were no doubts in the morphological determination of the species affiliation.


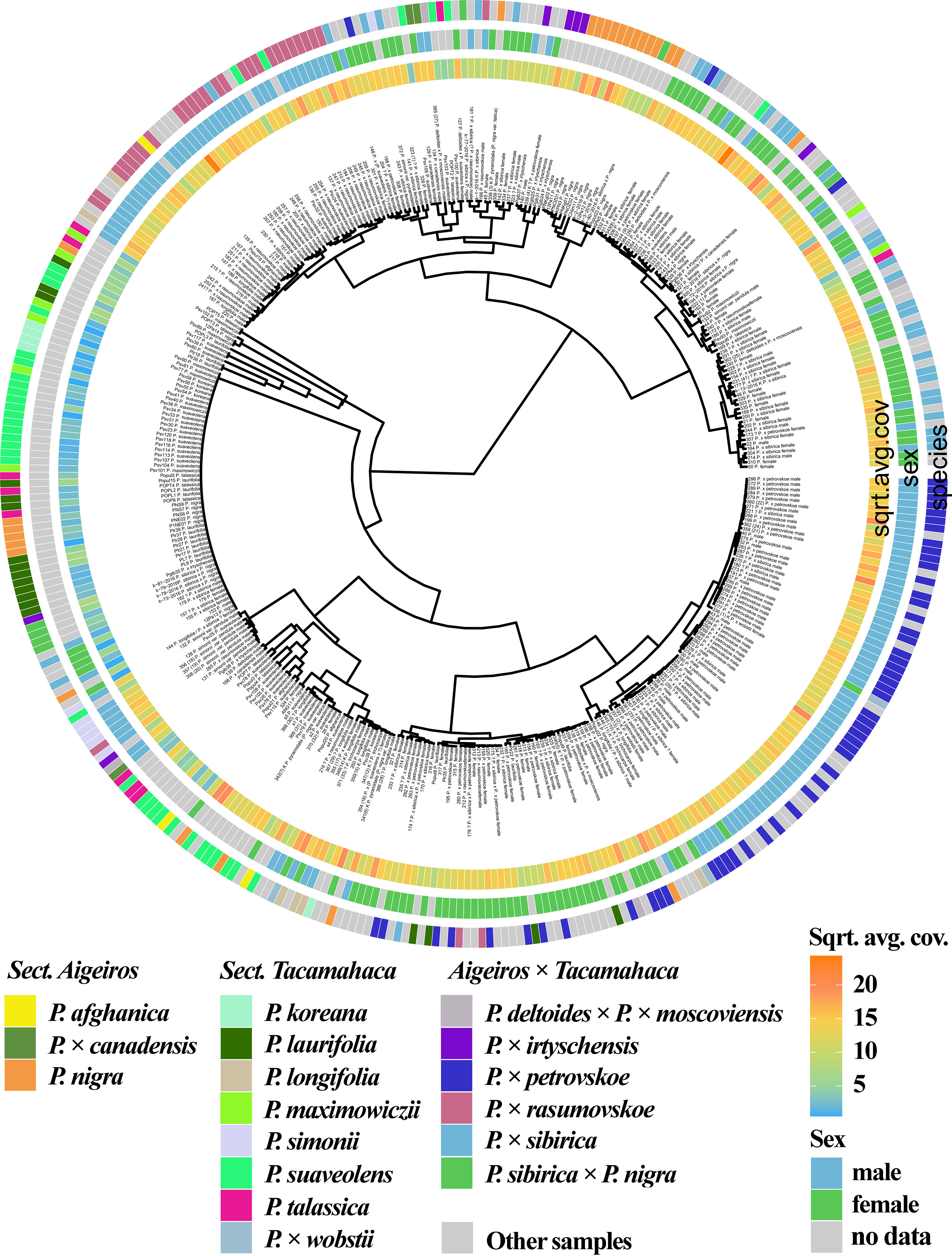


**Supplementary Data 6G.** Dendrogram based on deep sequencing data for gene *6* sequences. Colors corresponding to species and hybrids mark only accessions for which there were no doubts in the morphological determination of the species affiliation.


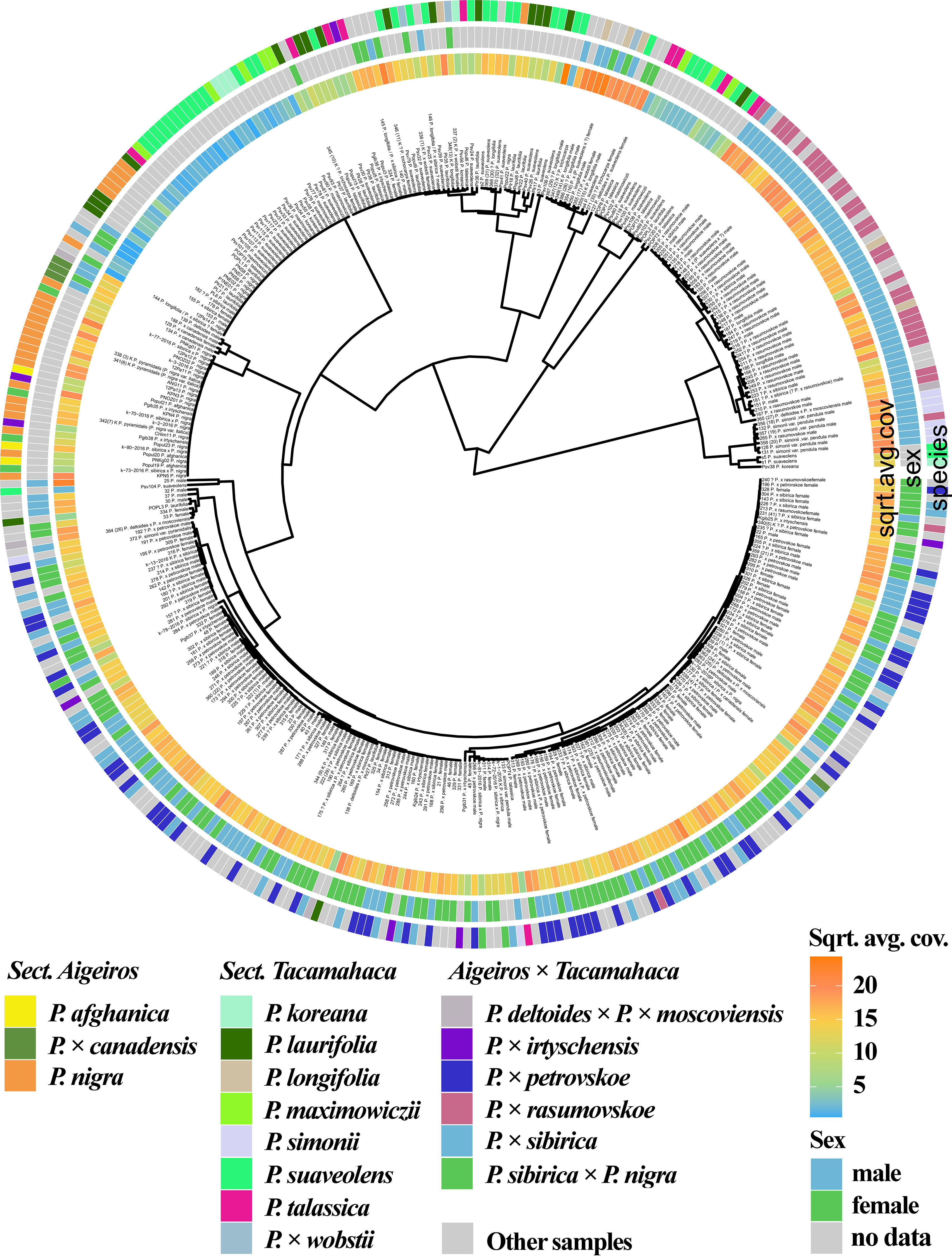


**Supplementary Data 6H.** Dendrogram based on deep sequencing data for gene *15* sequences. Colors corresponding to species and hybrids mark only accessions for which there were no doubts in the morphological determination of the species affiliation.


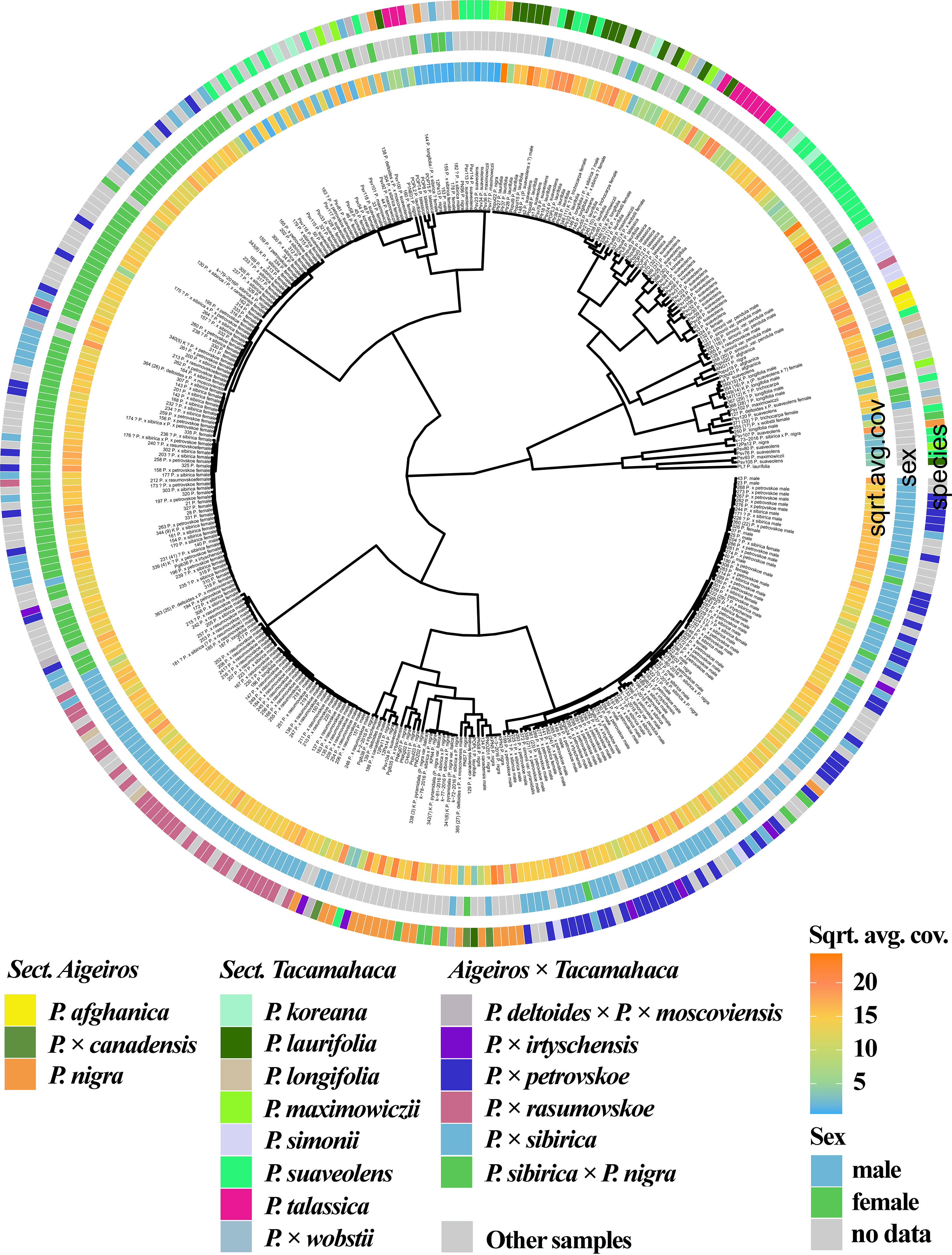


**Supplementary Data 6I.** Dendrogram based on deep sequencing data for gene *16* sequences. Colors corresponding to species and hybrids mark only accessions for which there were no doubts in the morphological determination of the species affiliation.


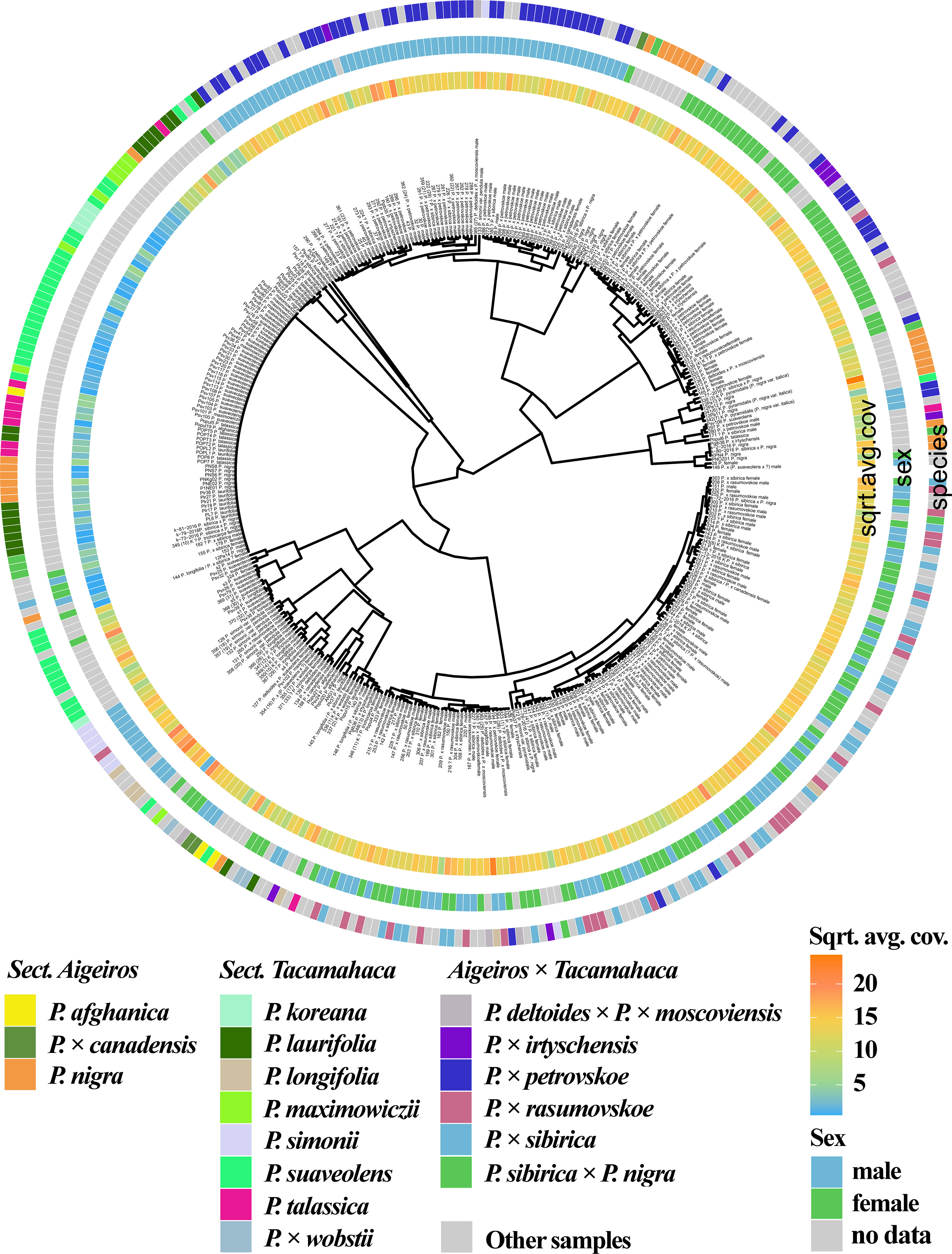


**Supplementary Data 6J.** Dendrogram based on deep sequencing data for *X18* sequences. Colors corresponding to species and hybrids mark only accessions for which there were no doubts in the morphological determination of the species affiliation.


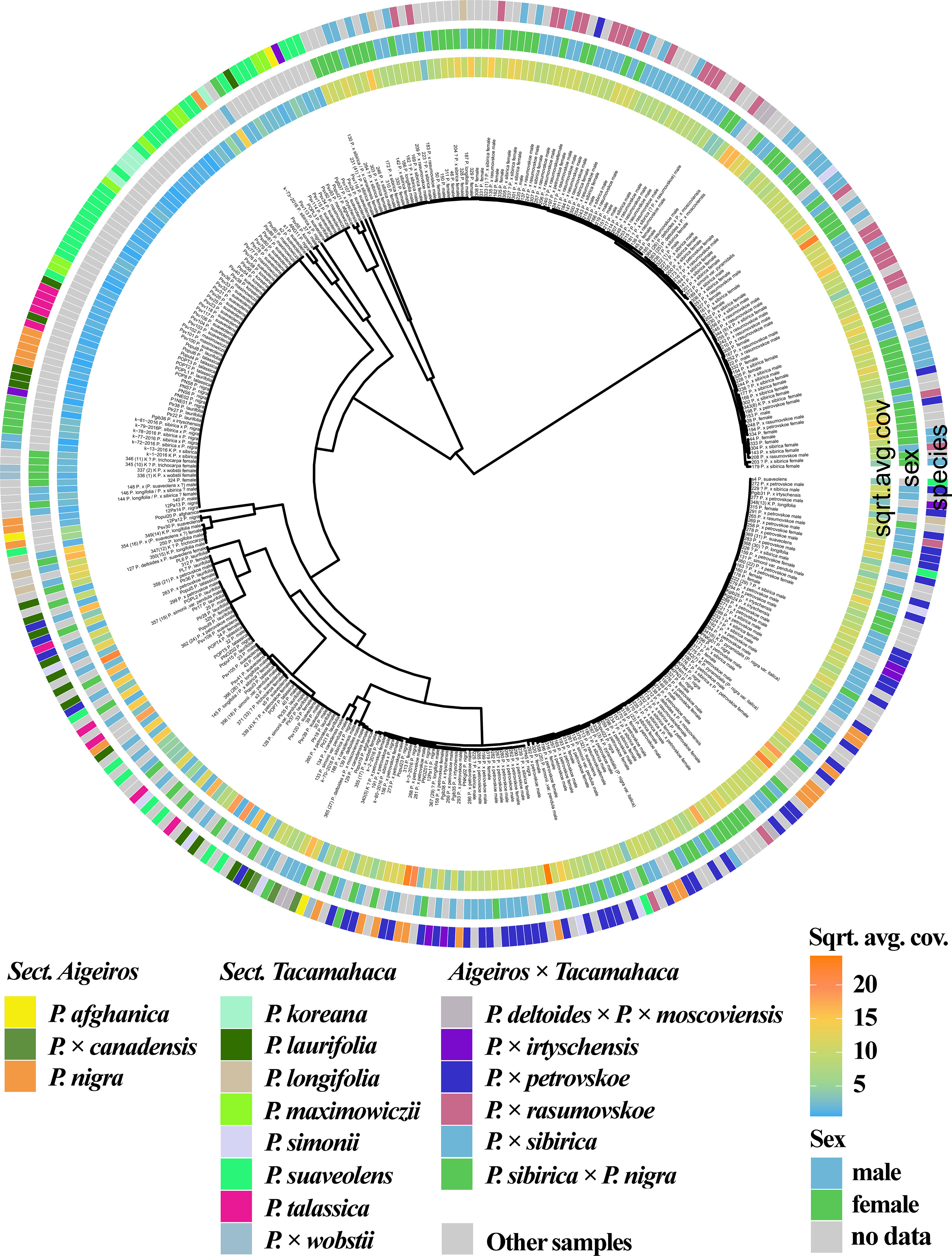


**Supplementary Data 6K.** Dendrogram based on deep sequencing data for *DSH 5* sequences. Colors corresponding to species and hybrids mark only accessions for which there were no doubts in the morphological determination of the species affiliation.


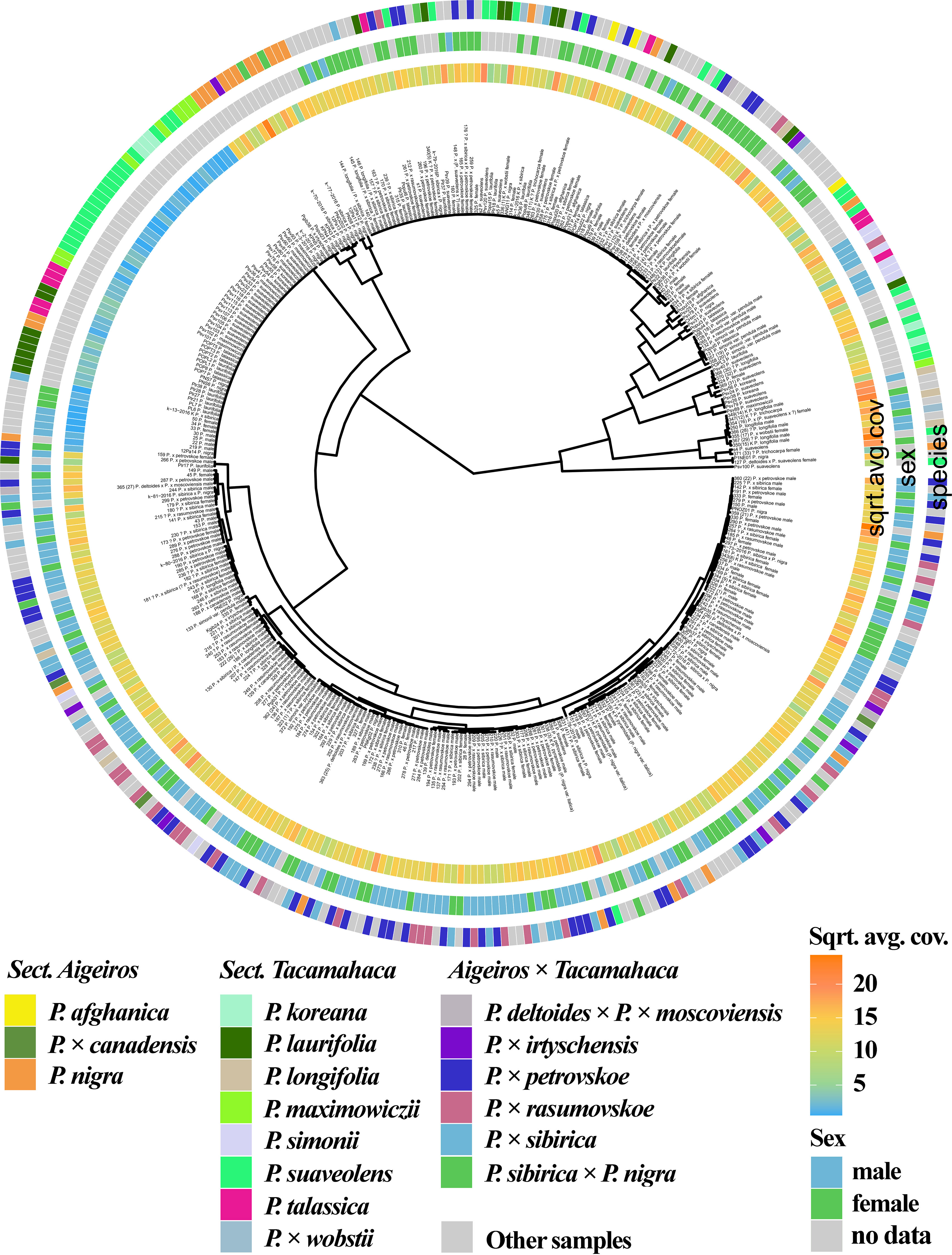


**Supplementary Data 6L.** Dendrogram based on deep sequencing data for *DSH 12* sequences. Colors corresponding to species and hybrids mark only accessions for which there were no doubts in the morphological determination of the species affiliation.


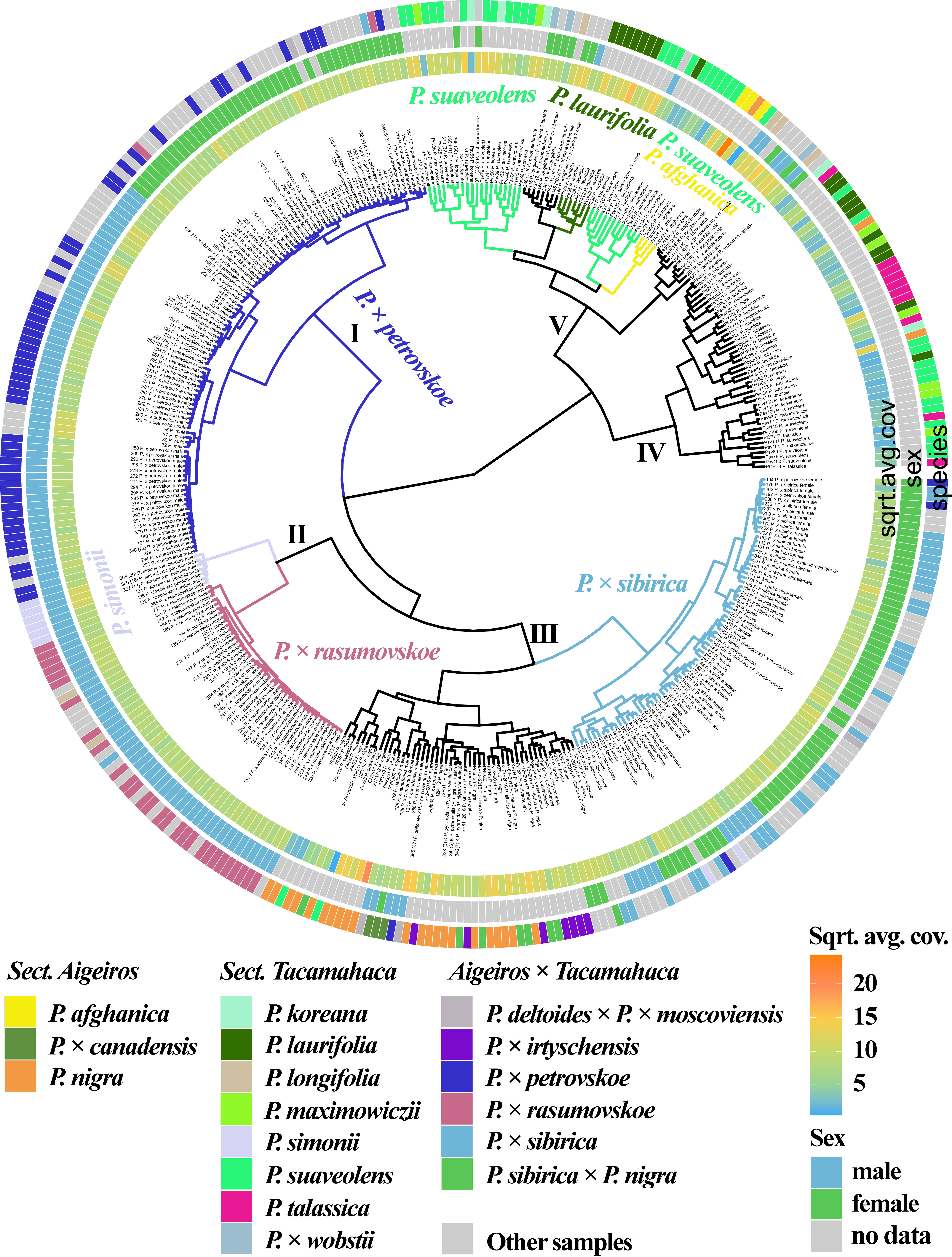


**Supplementary Data 6M.** Dendrogram based on deep sequencing data for NTS 5S rDNA, ITS, *DSH 2*, *DSH 5*, *DSH 8*, *DSH 12*, *DSH 29*, *6*, *15*, *16*, and *X18* sequences. Colors corresponding to species and hybrids mark only accessions for which there were no doubts in the morphological determination of the species affiliation.
