# Supplementary material for: Sex-determining region complements traditionally used in phylogenetic studies nuclear and chloroplast sequences in investigation of Aigeiros Duby and Tacamahaca Spach poplars (genus Populus L., Salicaceae)
Source: Front Plant Sci. 2023 Oct 4;14:1204899. doi: 10.3389/fpls.2023.1204899 (PMC10582643; doi:10.3389/fpls.2023.1204899)
Supplement: Supplementary file 7 [file DataSheet_7.docx]

**Supplementary Data 7.** Dendrograms for 379 poplar accessions of sections *Aigeiros* and *Tacamahaca* based on deep sequencing data for NTS 5S rDNA, ITS, *DSH 2*, *DSH 5*, *DSH 8*, *DSH 12*, *DSH 29*, *6*, *15*, *16*, *X18*, *trnG-psbK-psbI*, *rps2-rpoC2*, *rpoC2-rpoC1*, the sex-determining region (SDR), and the *ARR17* gene sequences, as well as their combinations (**7A** – *trnG‐psbK-psbI*; **7B** – *rps2‐rpoC2*; **7C** – *rpoC2-rpoC1*; **7D** – *trnG‐psbK-psbI*, *rps2‐rpoC2*, and *rpoC2-rpoC1*; **7E** – SDR; **7F** – *ARR17*; **7G** – NTS 5S rDNA, ITS, *DSH 2*, *DSH 5*, *DSH 8*, *DSH 12*, *DSH 29*, *6*, *15*, *16*, *X18*, *trnG‐psbK-psbI*, *rps2‐rpoC2*, *rpoC2-rpoC1*, SDR, and *ARR17*).


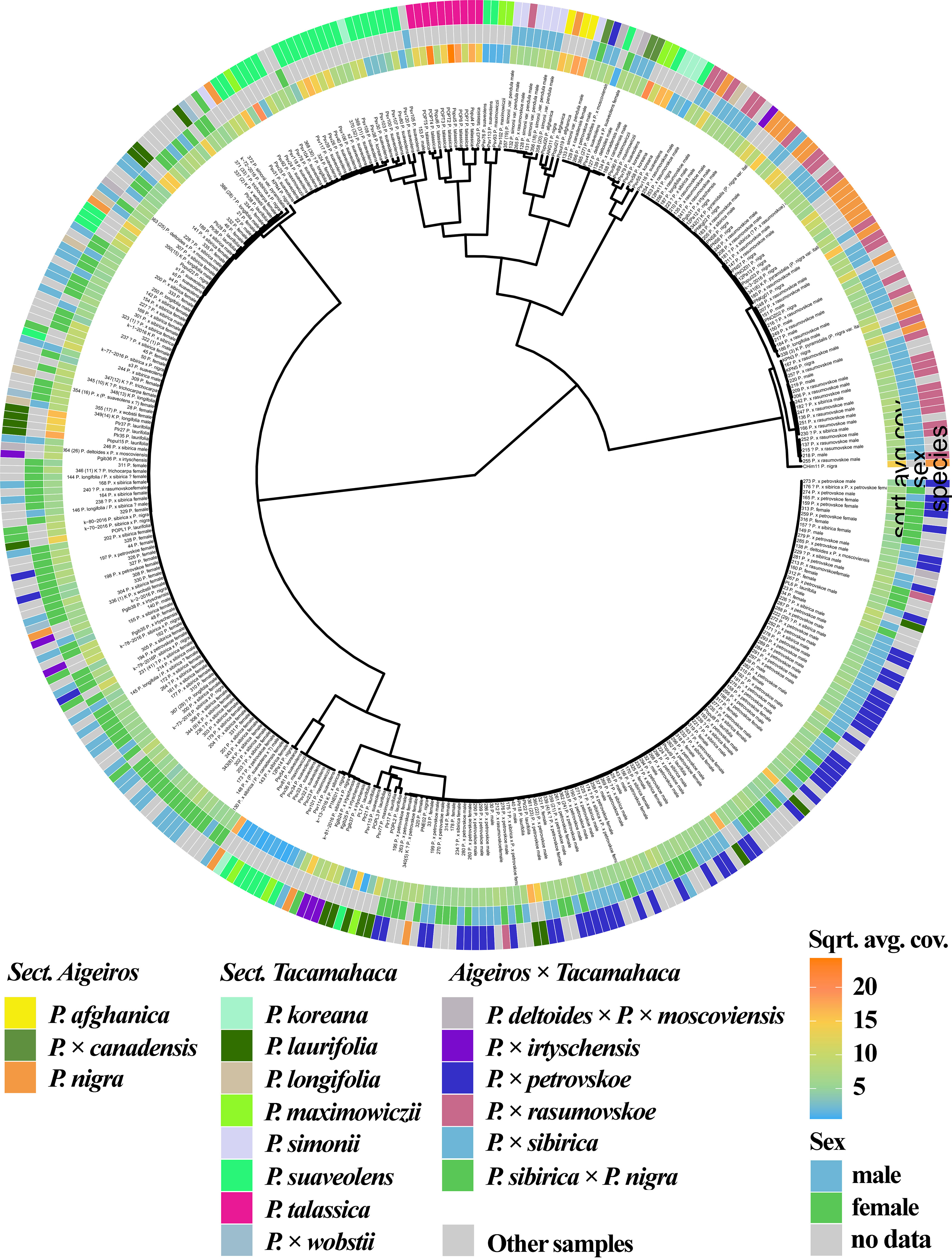


**Supplementary Data 7A.** Dendrogram based on deep sequencing data for *trnG‐psbK-psbI* sequences. Colors corresponding to species and hybrids mark only accessions for which there were no doubts in the morphological determination of the species affiliation.


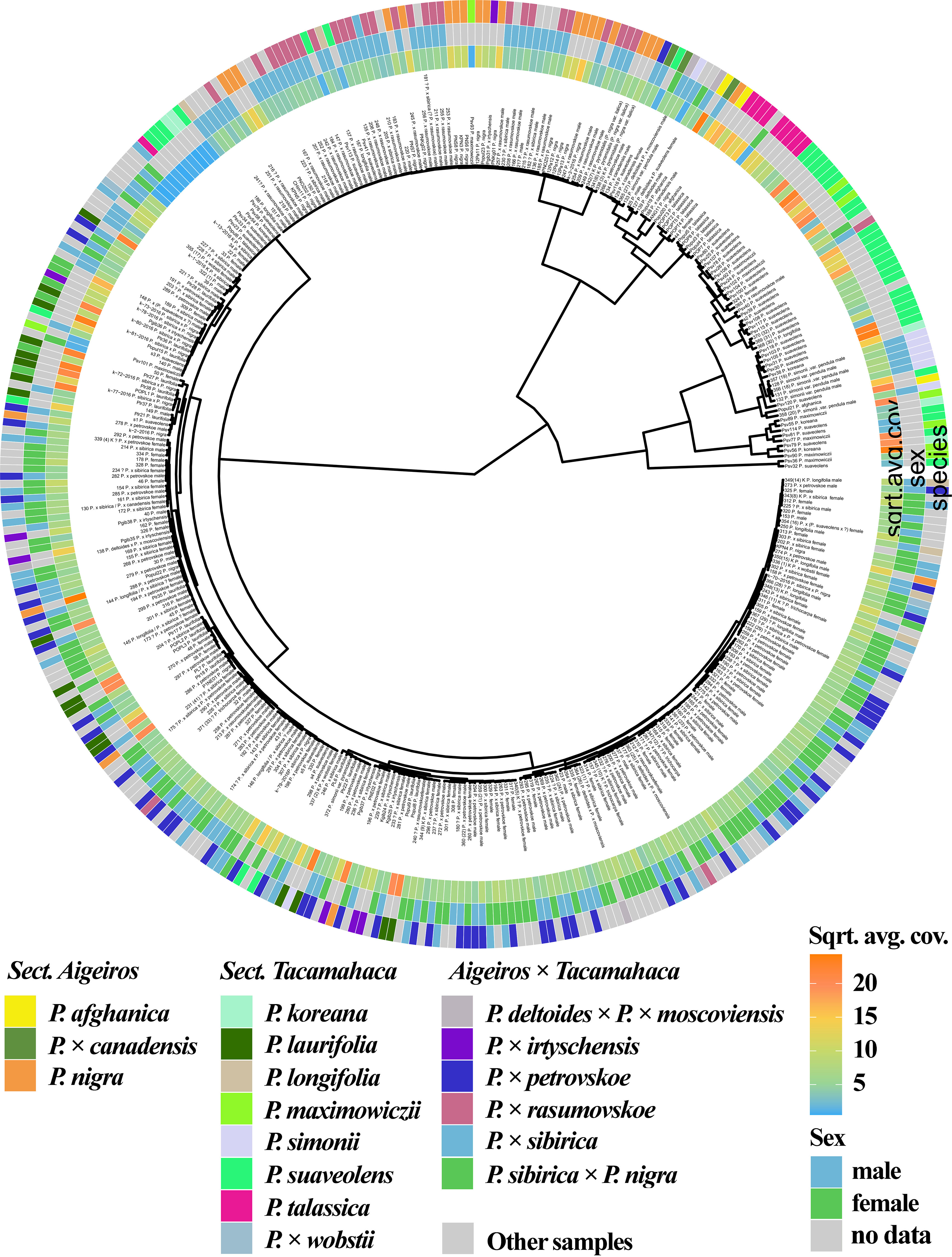


**Supplementary Data 7B.** Dendrogram based on deep sequencing data for *rps2-rpoC2* sequences. Colors corresponding to species and hybrids mark only accessions for which there were no doubts in the morphological determination of the species affiliation.


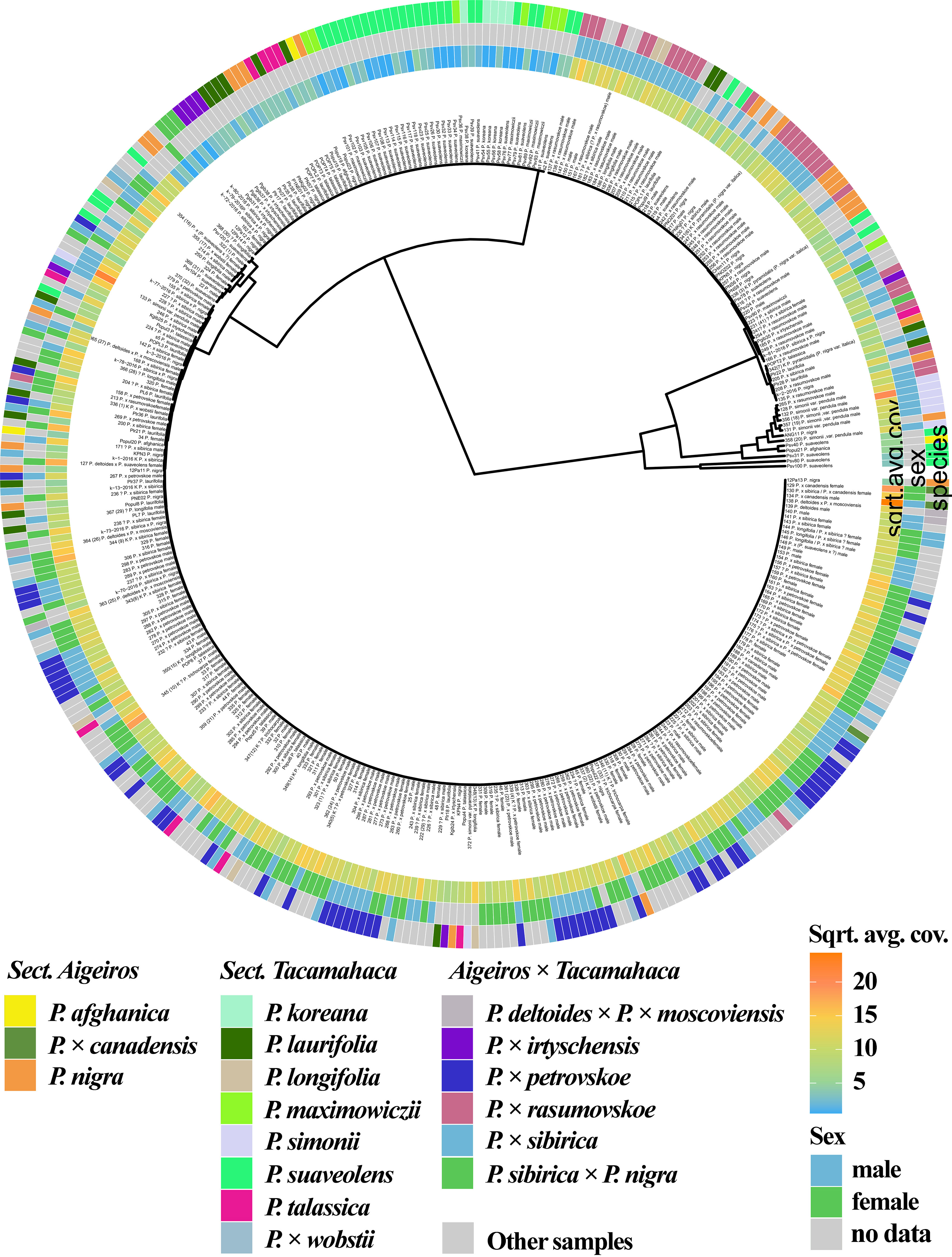


**Supplementary Data 7C.** Dendrogram based on deep sequencing data for *rpoC2-rpoC1* sequences. Colors corresponding to species and hybrids mark only accessions for which there were no doubts in the morphological determination of the species affiliation.


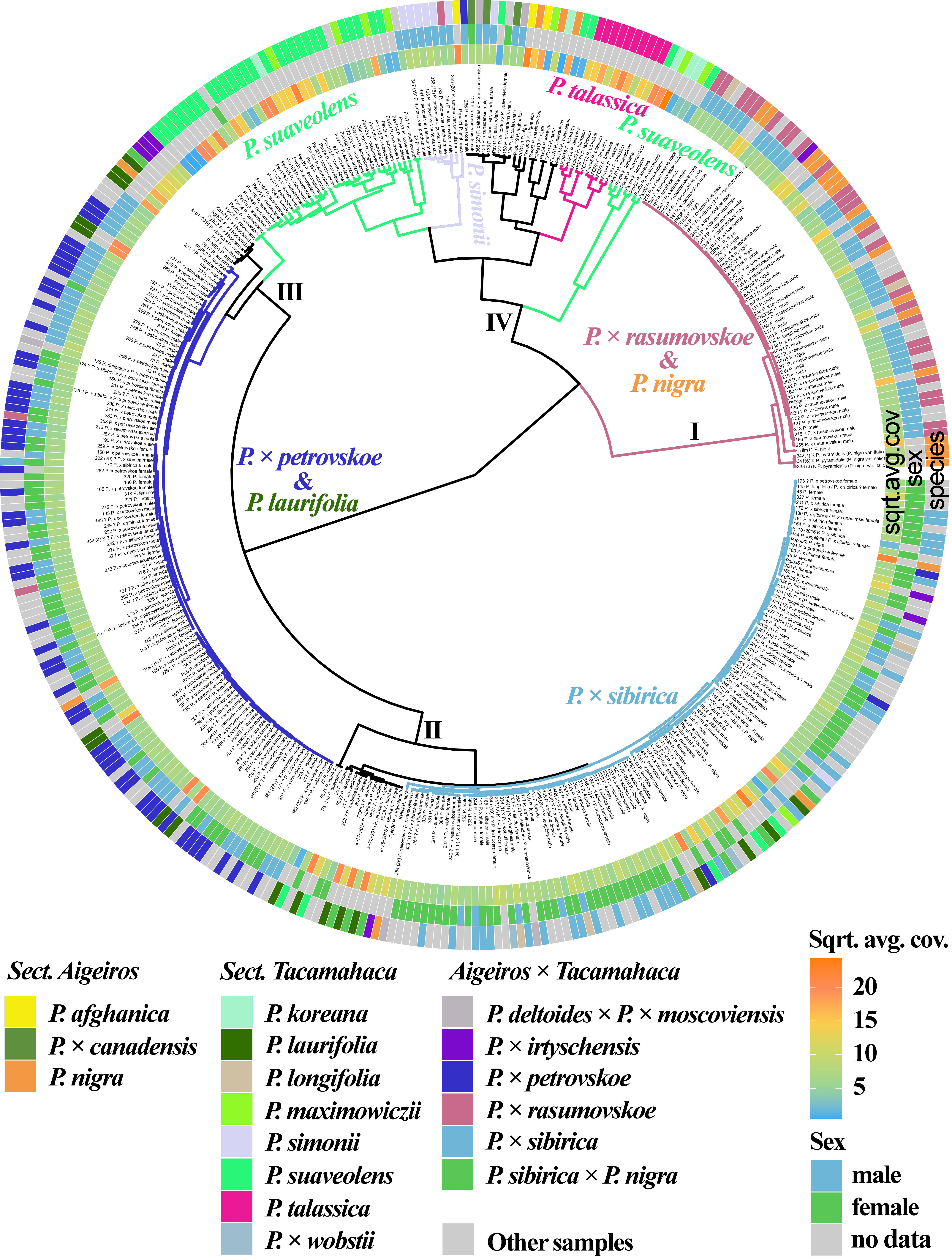


**Supplementary Data 7D.** Dendrogram based on deep sequencing data for *trnG‐psbK-psbI*, *rps2-rpoC2*, and *rpoC2-rpoC1* sequences. Colors corresponding to species and hybrids mark only accessions for which there were no doubts in the morphological determination of the species affiliation.


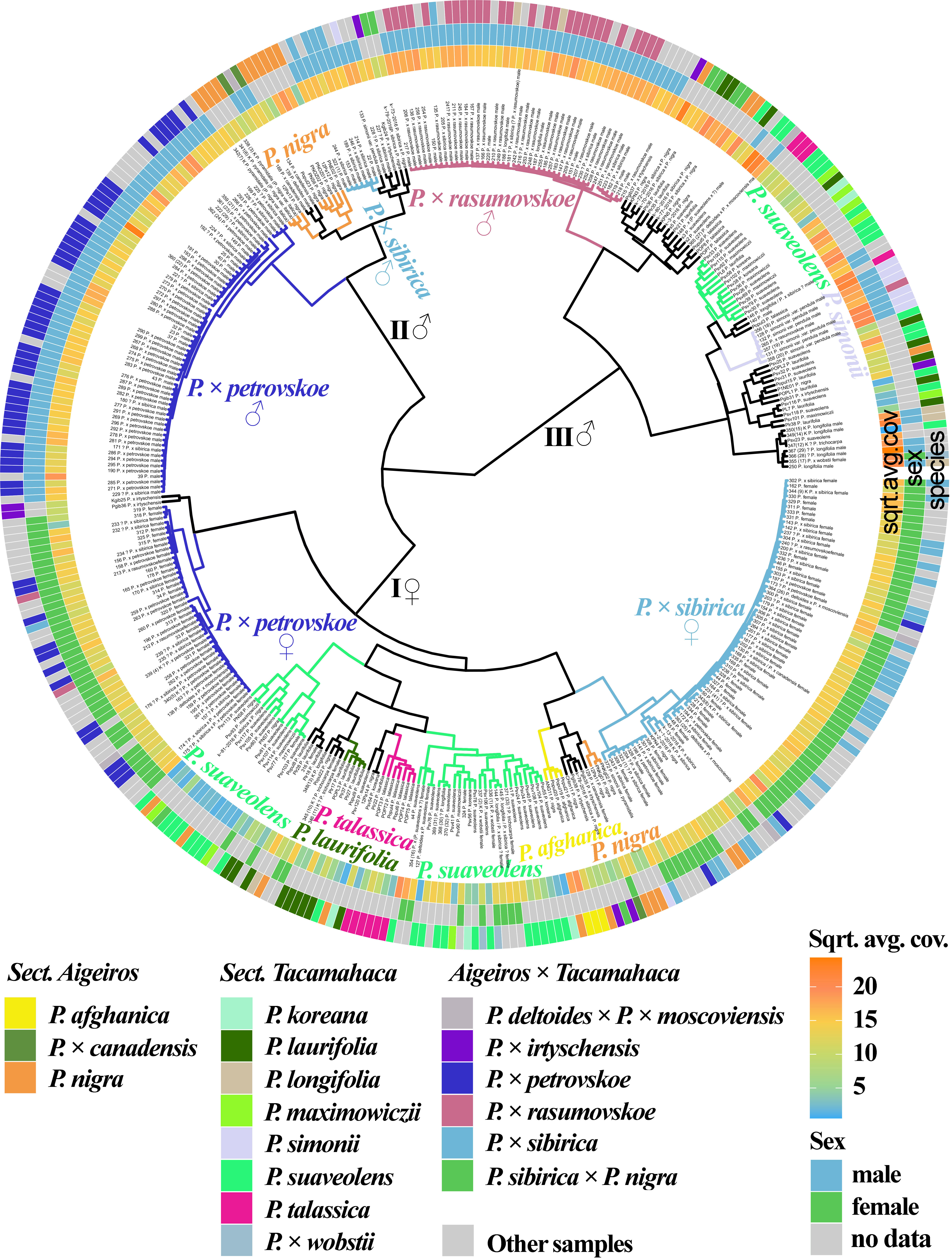


**Supplementary Data 7E.** Dendrogram based on deep sequencing data for the SDR sequences. Colors corresponding to species and hybrids mark only accessions for which there were no doubts in the morphological determination of the species affiliation.


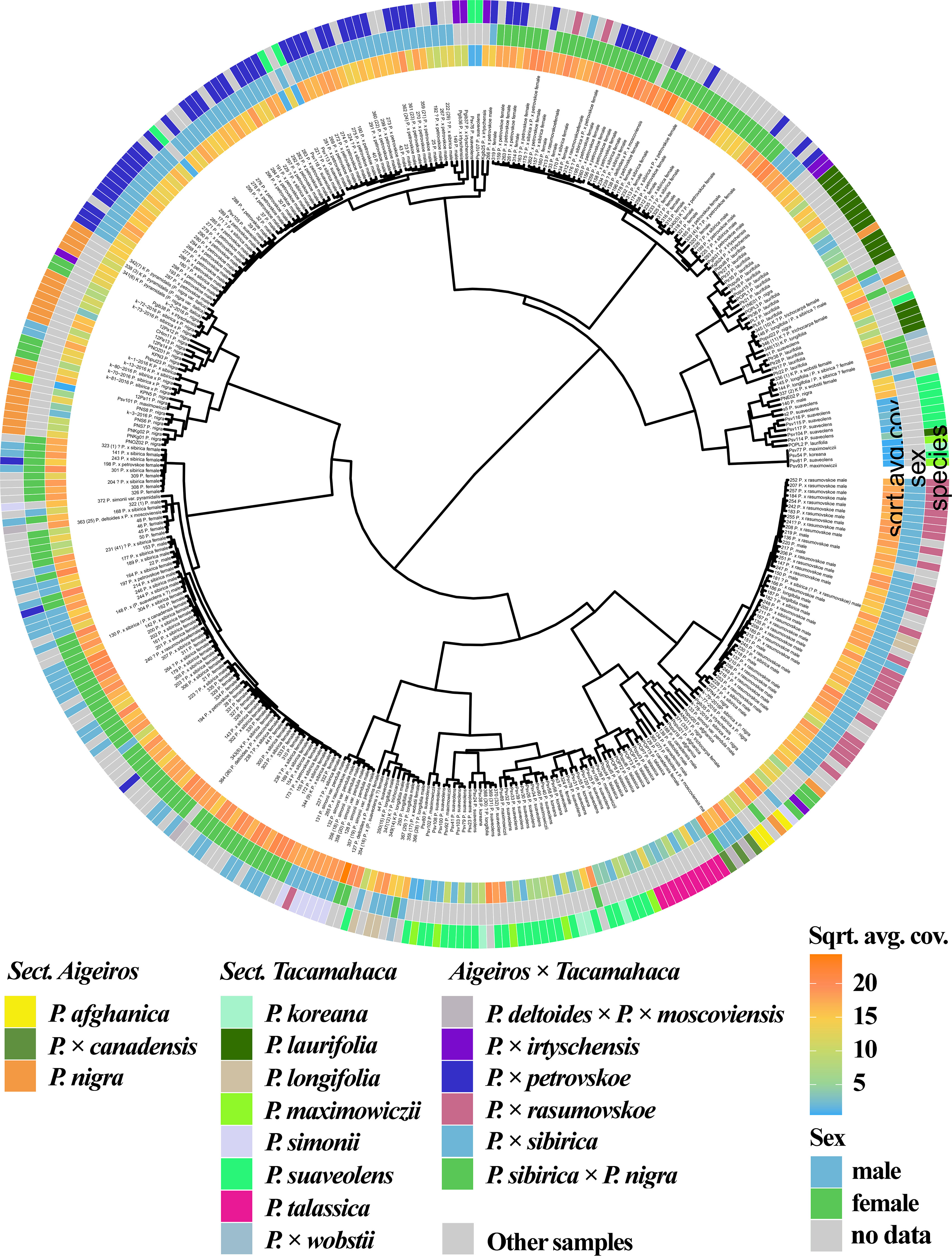


**Supplementary Data 7F.** Dendrogram based on deep sequencing data for the *ARR17* gene sequences. Colors corresponding to species and hybrids mark only accessions for which there were no doubts in the morphological determination of the species affiliation.


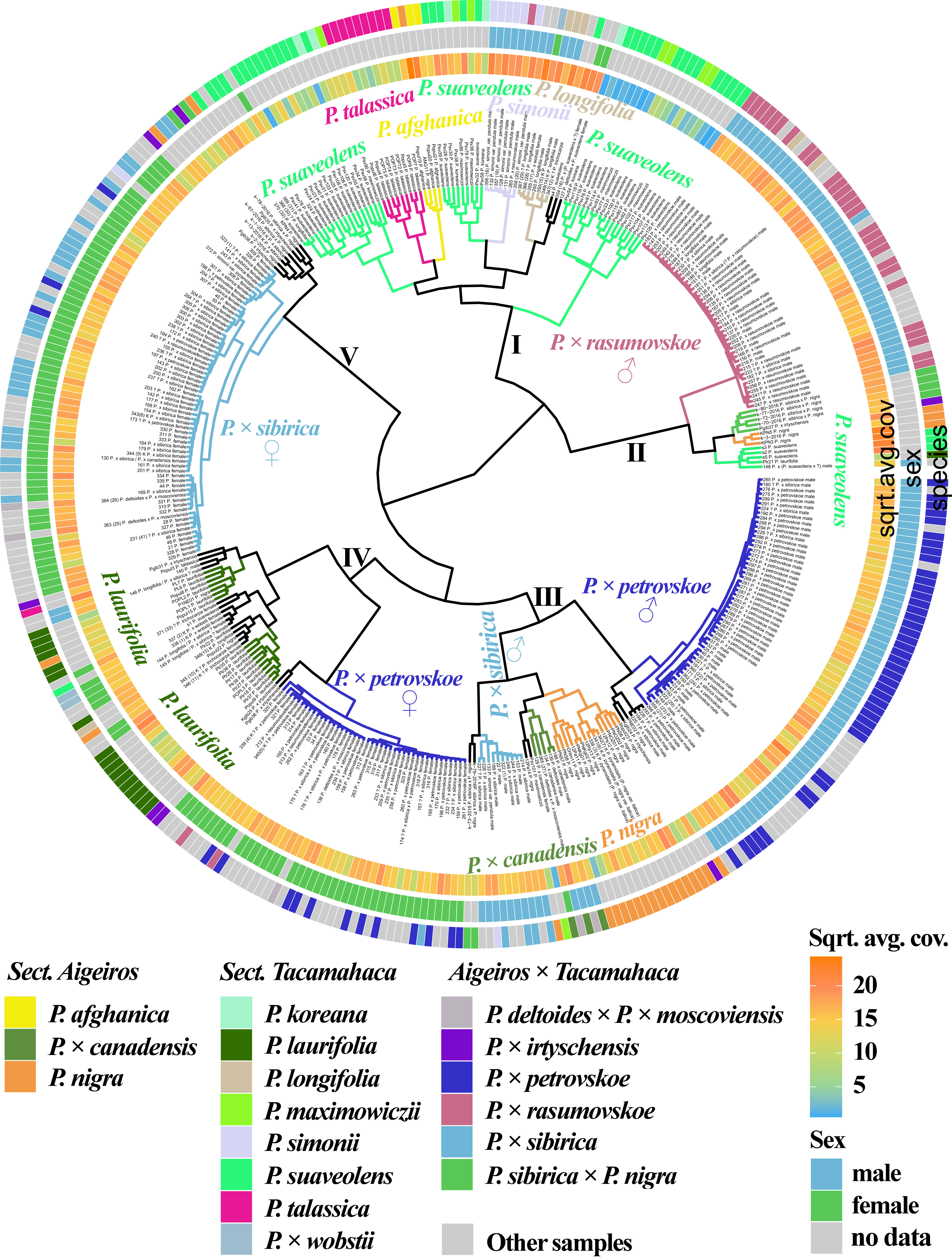


**Supplementary Data 7G.** Dendrogram based on deep sequencing data for NTS 5S rDNA, ITS, *DSH 2*, *DSH 5*, *DSH 8*, *DSH 12*, *DSH 29*, *6*, *15*, *16*, *X18*, *trnG‐psbK-psbI*, *rps2-rpoC2*, *rpoC2-rpoC1*, SDR, and *ARR17* sequences. Colors corresponding to species and hybrids mark only accessions for which there were no doubts in the morphological determination of the species affiliation.
