# Supplementary material for: Sex-determining region complements traditionally used in phylogenetic studies nuclear and chloroplast sequences in investigation of Aigeiros Duby and Tacamahaca Spach poplars (genus Populus L., Salicaceae)
Source: Front Plant Sci. 2023 Oct 4;14:1204899. doi: 10.3389/fpls.2023.1204899 (PMC10582643; doi:10.3389/fpls.2023.1204899)
Supplement: Supplementary file 8 [file DataSheet_8.pdf]

Supplementary Data 8. Dendrograms with bootstrap values for 379 poplar accessions of sections *Aigeiros* and *Tacamahaca* based on deep sequencing data for NTS 5S rDNA, ITS, *DSH 2*, *DSH 5*, *DSH 8*, *DSH 12*, *DSH 29*, *6*, *15*, *16*, *X18*, *trnG-psbK-psbI*, *rps2-rpoC2*, *rpoC2-rpoC1*, SDR, and *ARR17* as well as their combinations (**8A** – NTS 5S rDNA, ITS, *DSH 2*, *DSH 5*, *DSH 8*, *DSH 12*, *DSH 29*, *6*, *15*, *16*, *X18*, *trnG-psbK-psbI*, *rps2-rpoC2*, and *rpoC2-rpoC1*; **8B** – NTS 5S rDNA; **8C** – ITS; **8D** – *DSH 2*; **8E** – *DSH 8*; **8F** – *DSH 29*; **8G** – gene *6*; **8H** – gene *15*; **8I** – gene *16*; **8J** – *X18*; **8K** – *DSH 5*; **8L** – *DSH 12*; **8M** – NTS 5S rDNA, ITS, *DSH 2*, *DSH 5*, *DSH 8*, *DSH 12*, *DSH 29*, *6*, *15*, *16*, and *X18*; **8N** – *trnG-psbK-psbI*; **8O** – *rps2-rpoC2*; **8P** – *rpoC2-rpoC1*; **8Q** – *trnG-psbK-psbI*, *rps2-rpoC2*, and *rpoC2-rpoC1*; **8R** – SDR; **8S** – *ARR17*; **8T** – NTS 5S rDNA, ITS, *DSH 2*, *DSH 5*, *DSH 8*, *DSH 12*, *DSH 29*, *6*, *15*, *16*, *X18*, *trnG-psbK-psbI*, *rps2-rpoC2*, *rpoC2-rpoC1*, SDR, and *ARR17*) . Values at branches are approximately unbiased (AU) p-values (left, red), bootstrap probability (BP) values (right, green), and cluster labels (bottom, grey). Clusters with AU ≥ 80 are indicated by the red rectangles.

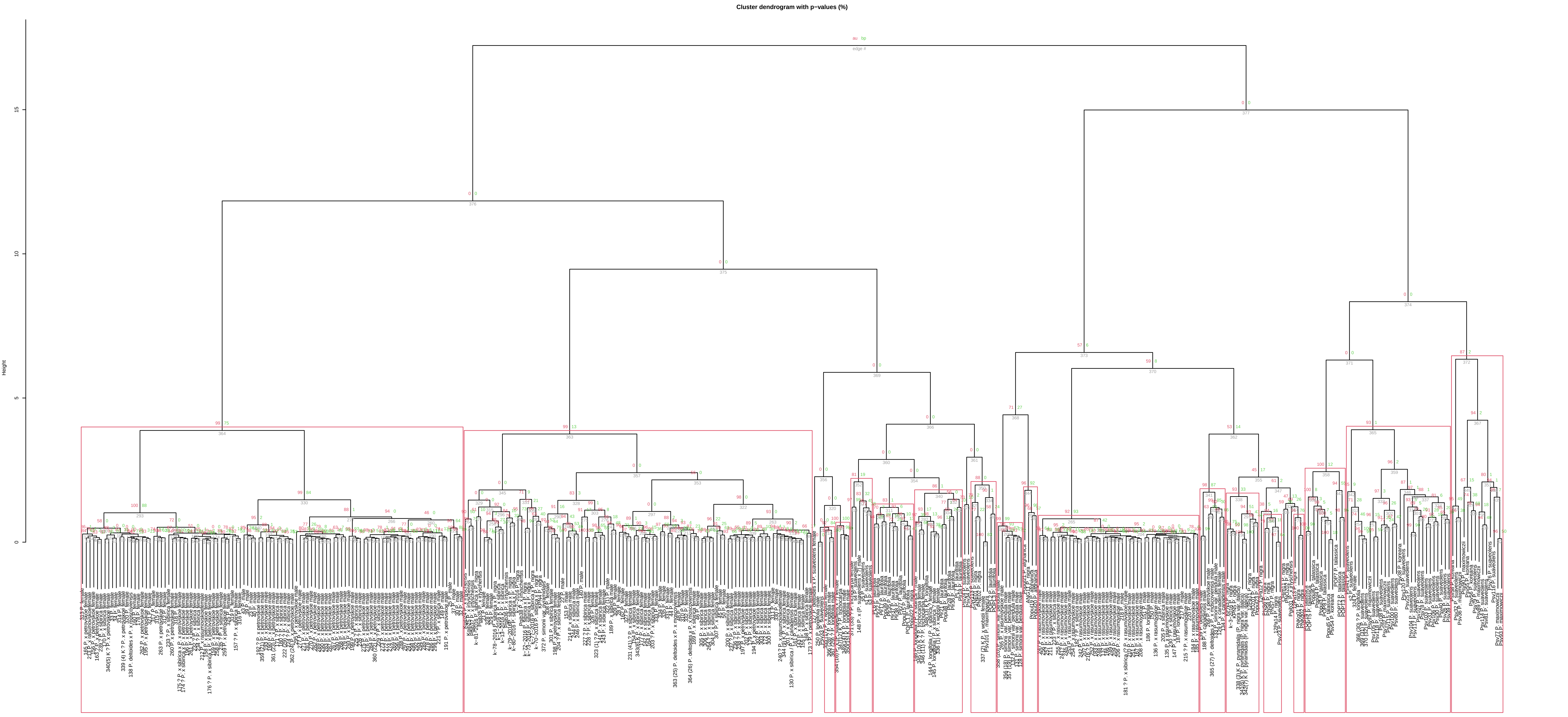

Supplementary Data 8A. Dendrogram based on deep sequencing data for NTS 5S rDNA, ITS, *DSH 2*, *DSH 5*, *DSH 8*, *DSH 12*, *DSH 29*, *6*, *15*, *16*, *X18*, *trnG-psbK-psbI*, *rps2-rpoC2*, and *rpoC2-rpoC1* sequences.

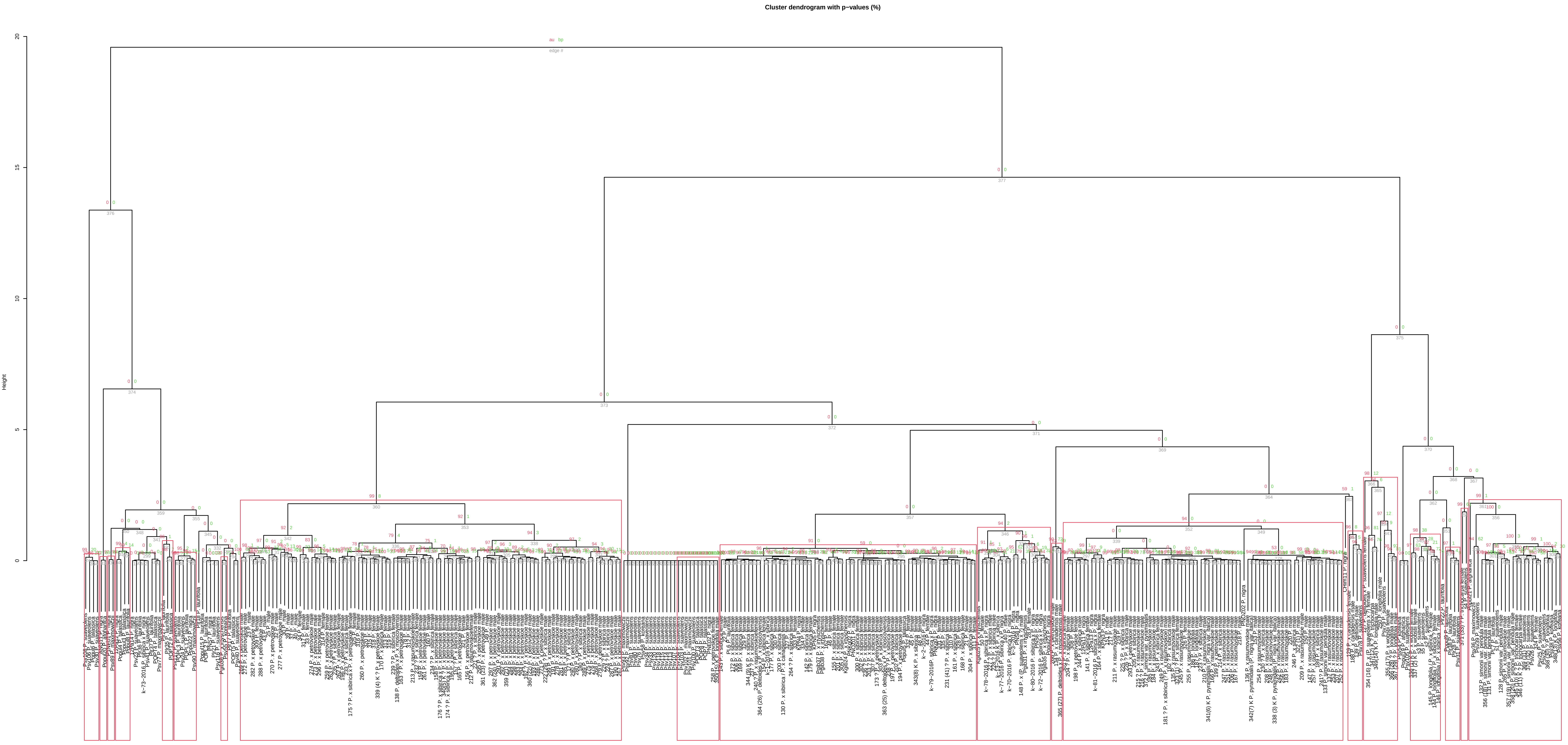

Supplementary Data 8B. Dendrogram based on deep sequencing data for NTS 5S rDNA sequences.

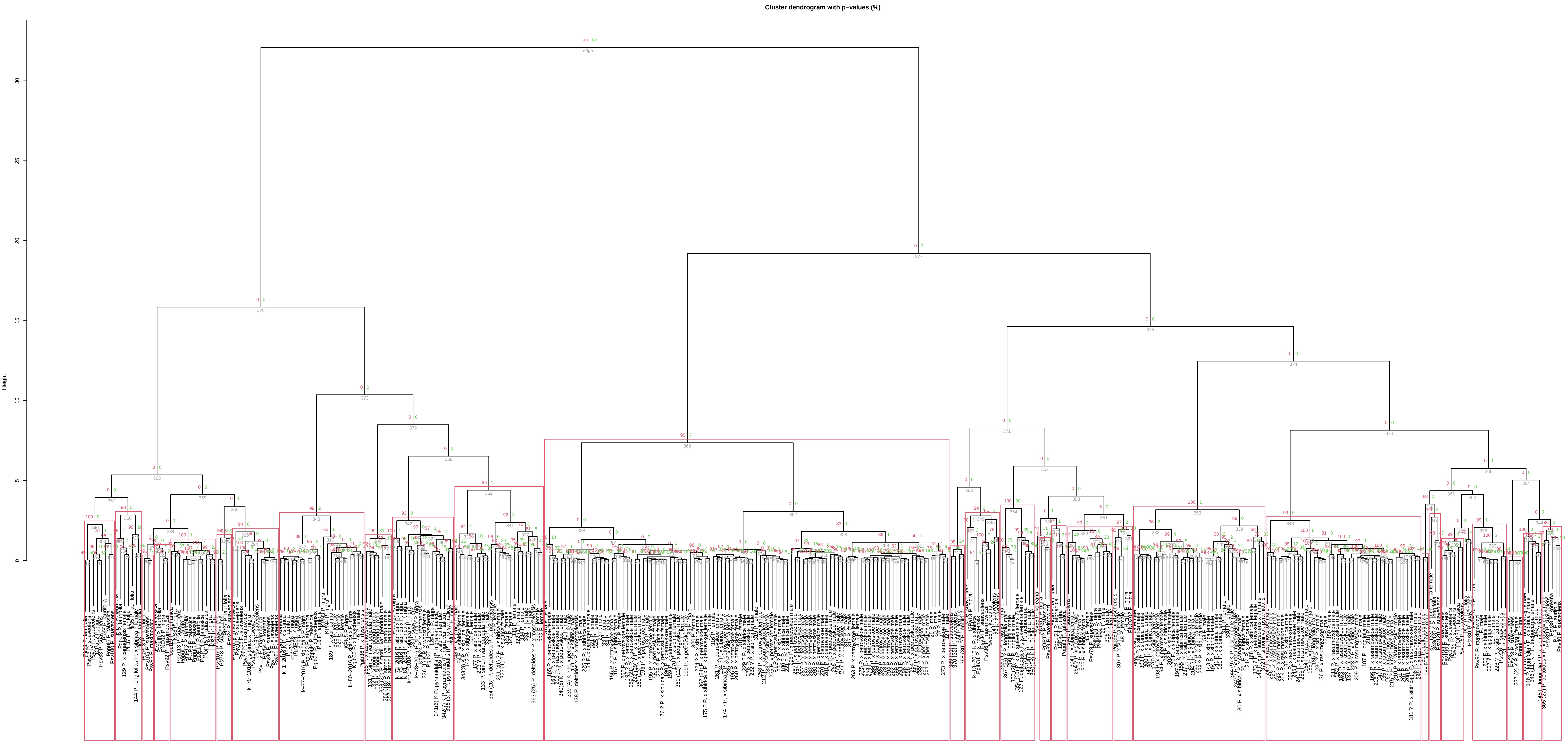

Supplementary Data 8C. Dendrogram based on deep sequencing data for ITS sequences.

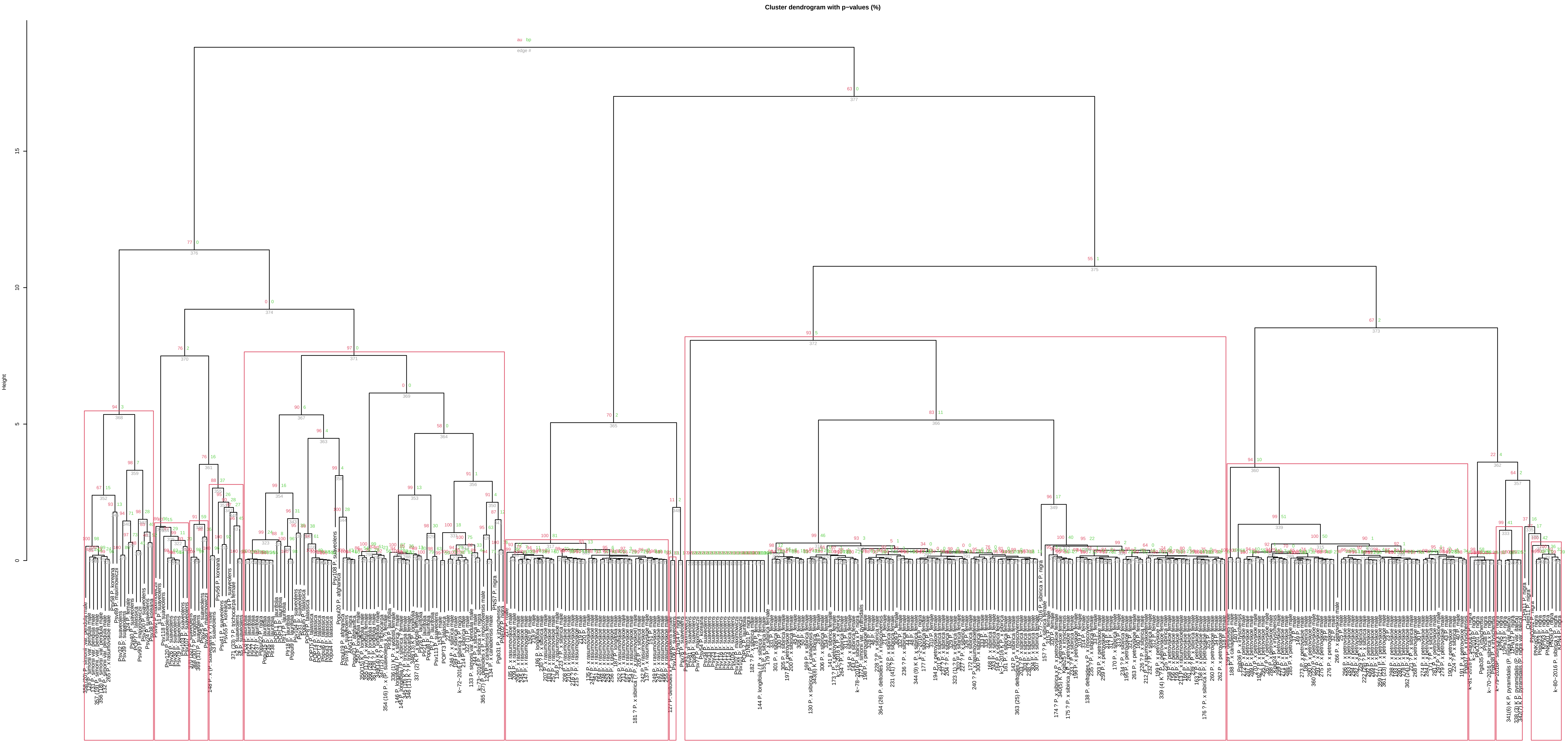

Supplementary Data 8D. Dendrogram based on deep sequencing data for *DSH 2* sequences.

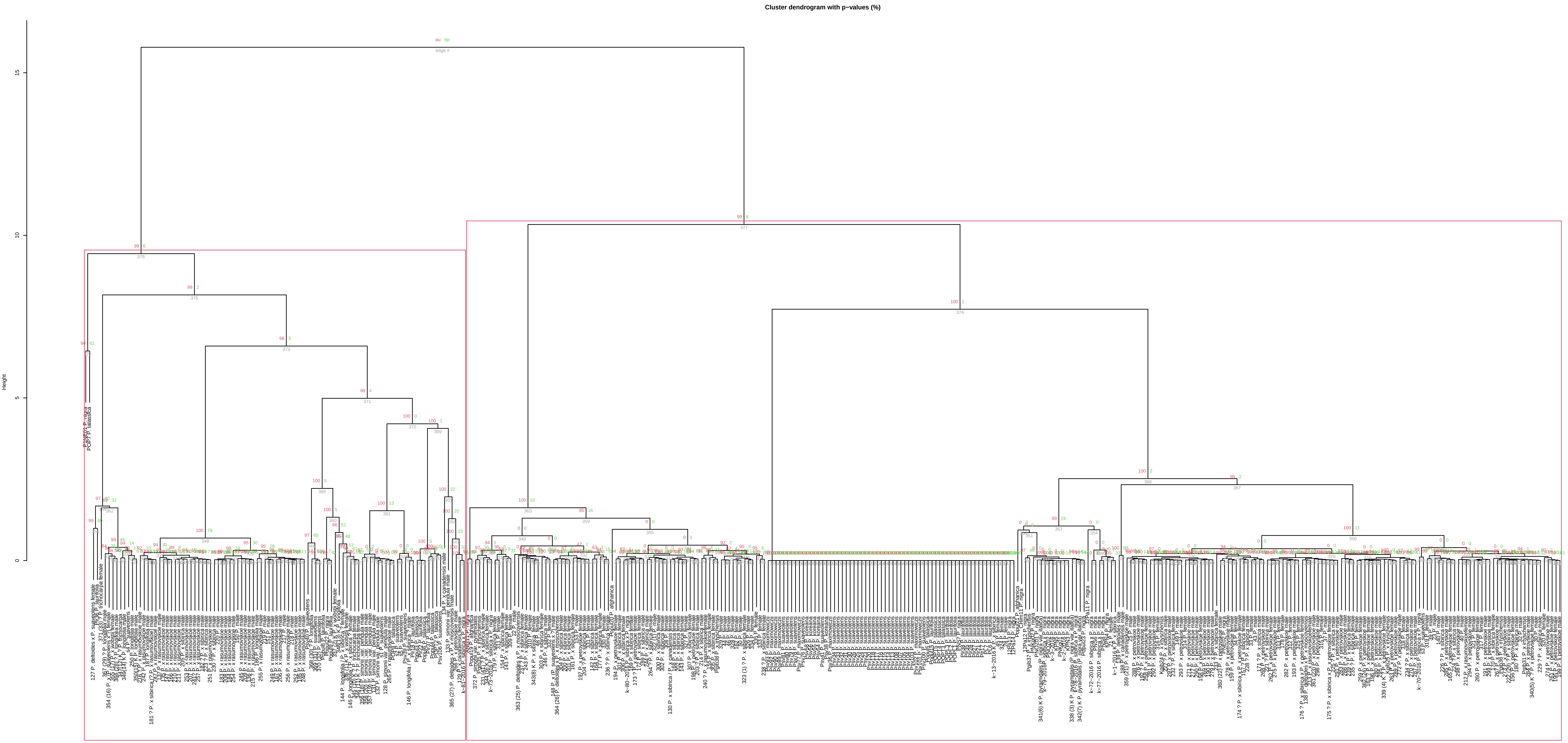

Supplementary Data 8E. Dendrogram based on deep sequencing data for *DSH 8* sequences.

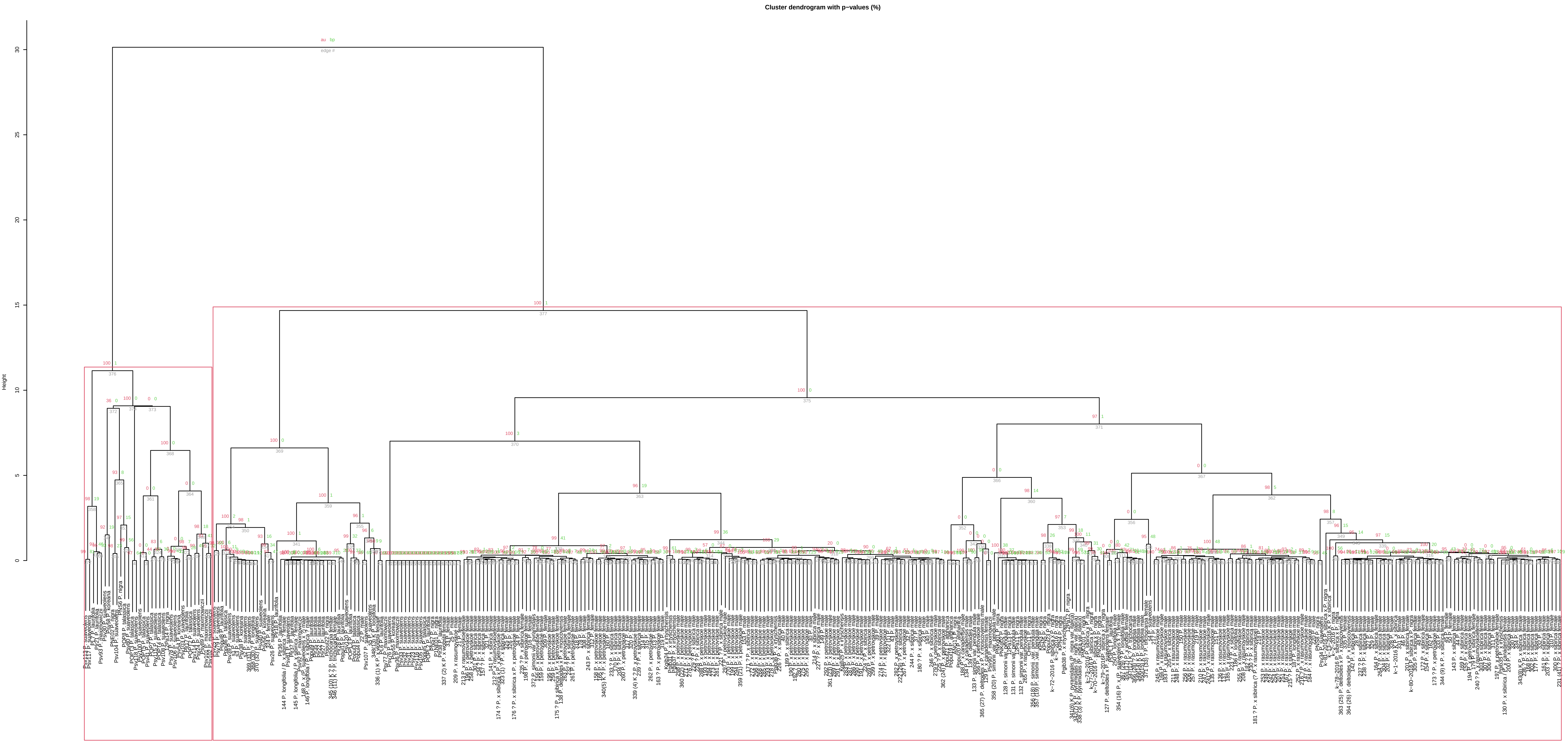

Supplementary Data 8F. Dendrogram based on deep sequencing data for *DSH 29* sequences.

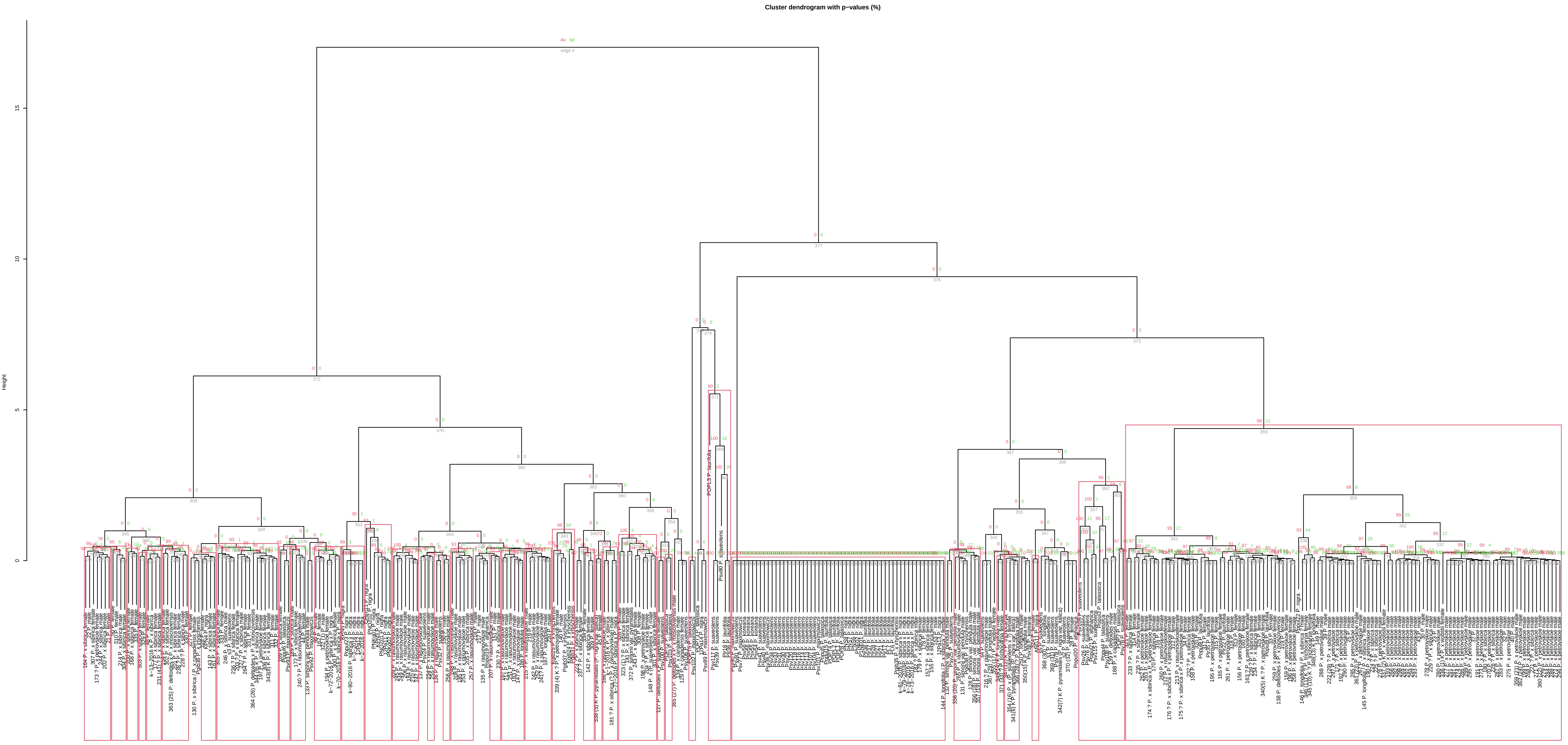

Supplementary Data 8G. Dendrogram based on deep sequencing data for gene *6* sequences.

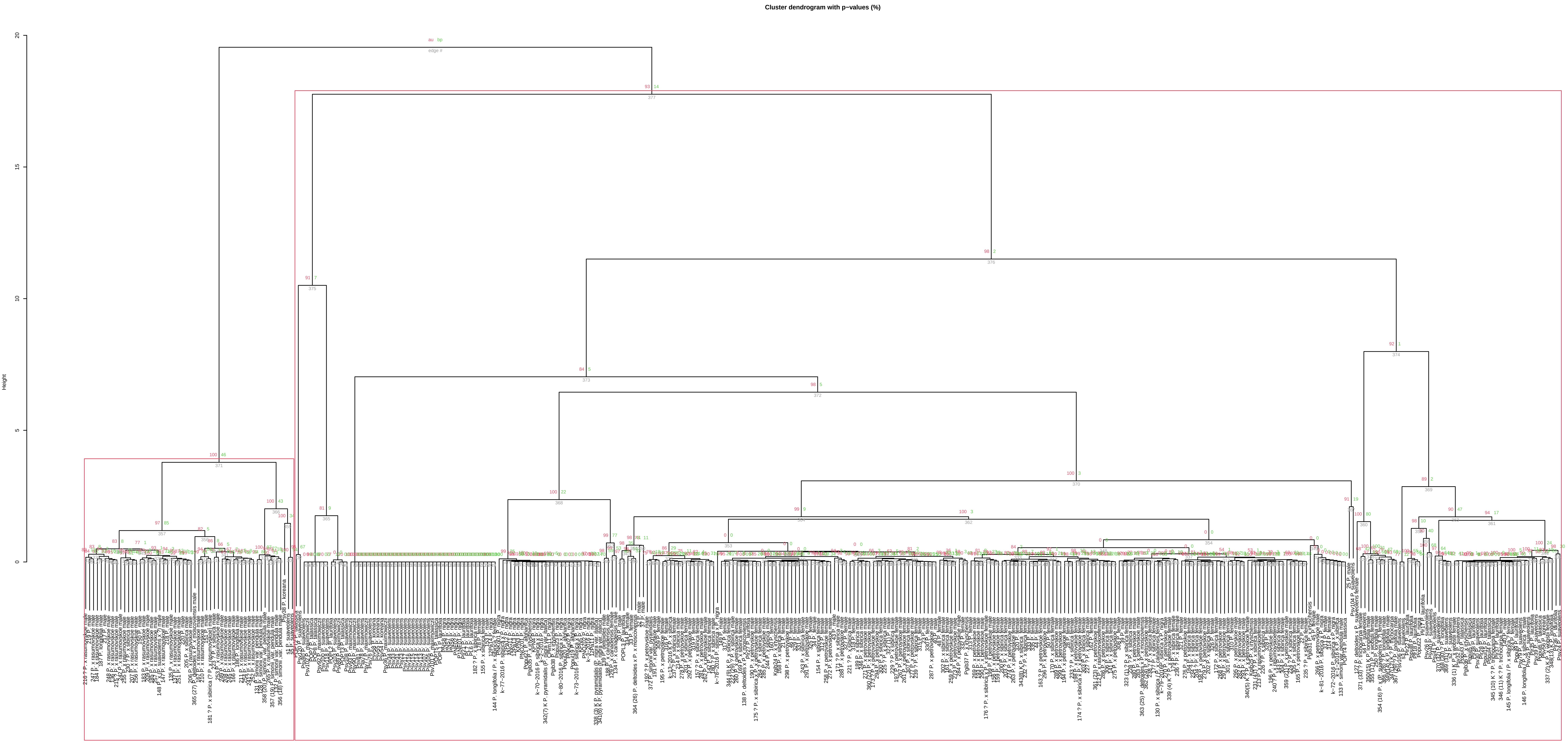

Supplementary Data 8H. Dendrogram based on deep sequencing data for gene *15* sequences.

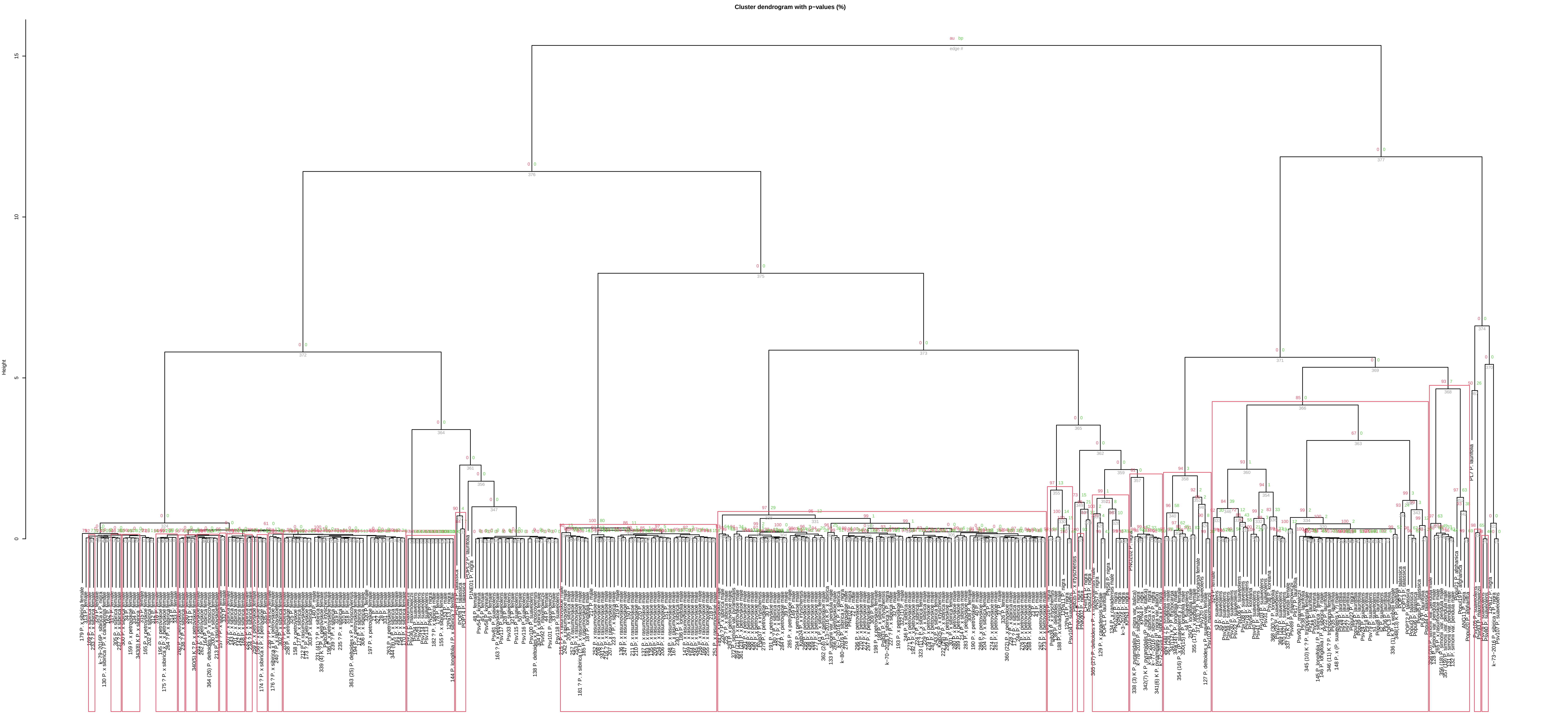

Supplementary Data 8I. Dendrogram based on deep sequencing data for gene *16* sequences.

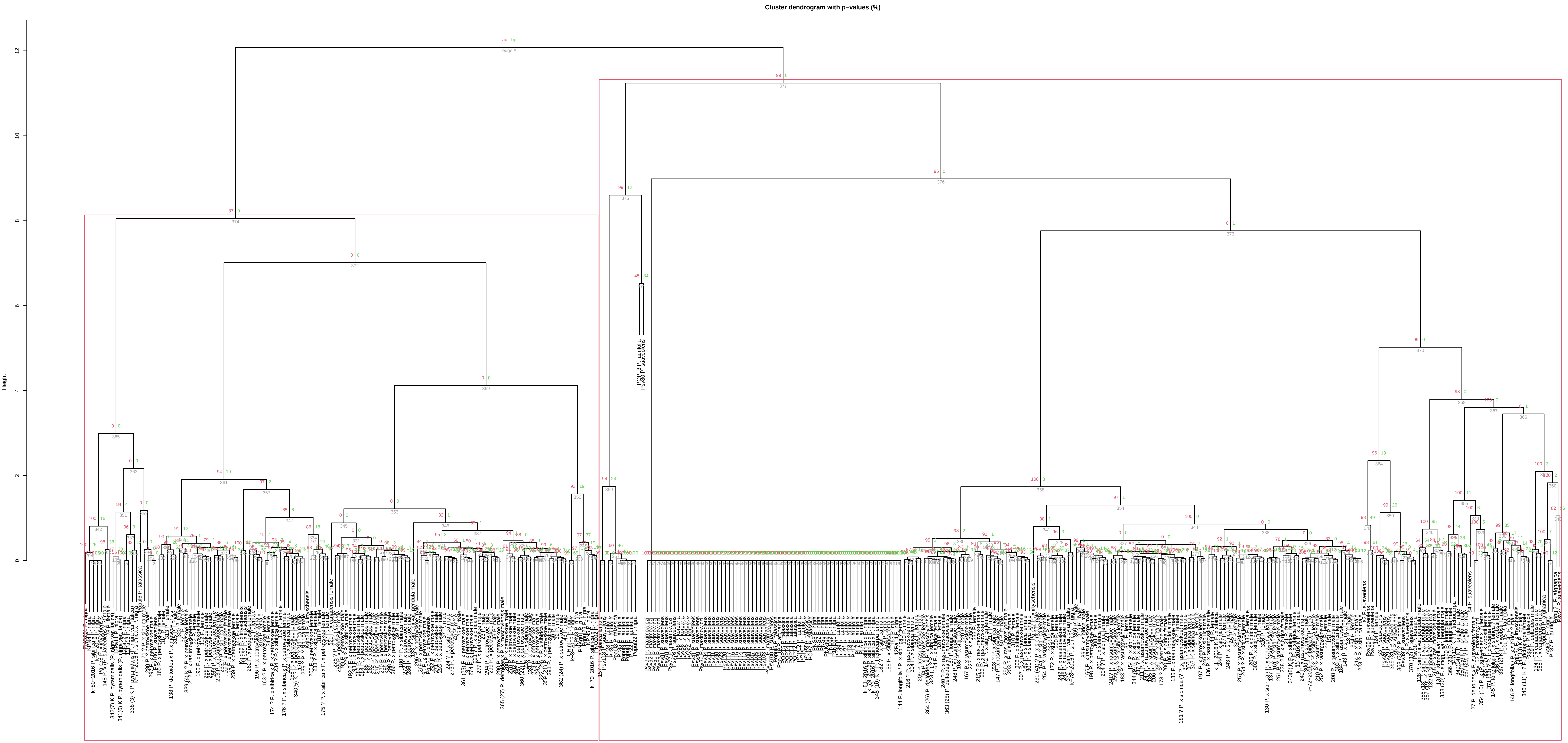

Supplementary Data 8J. Dendrogram based on deep sequencing data for *X18* sequences.

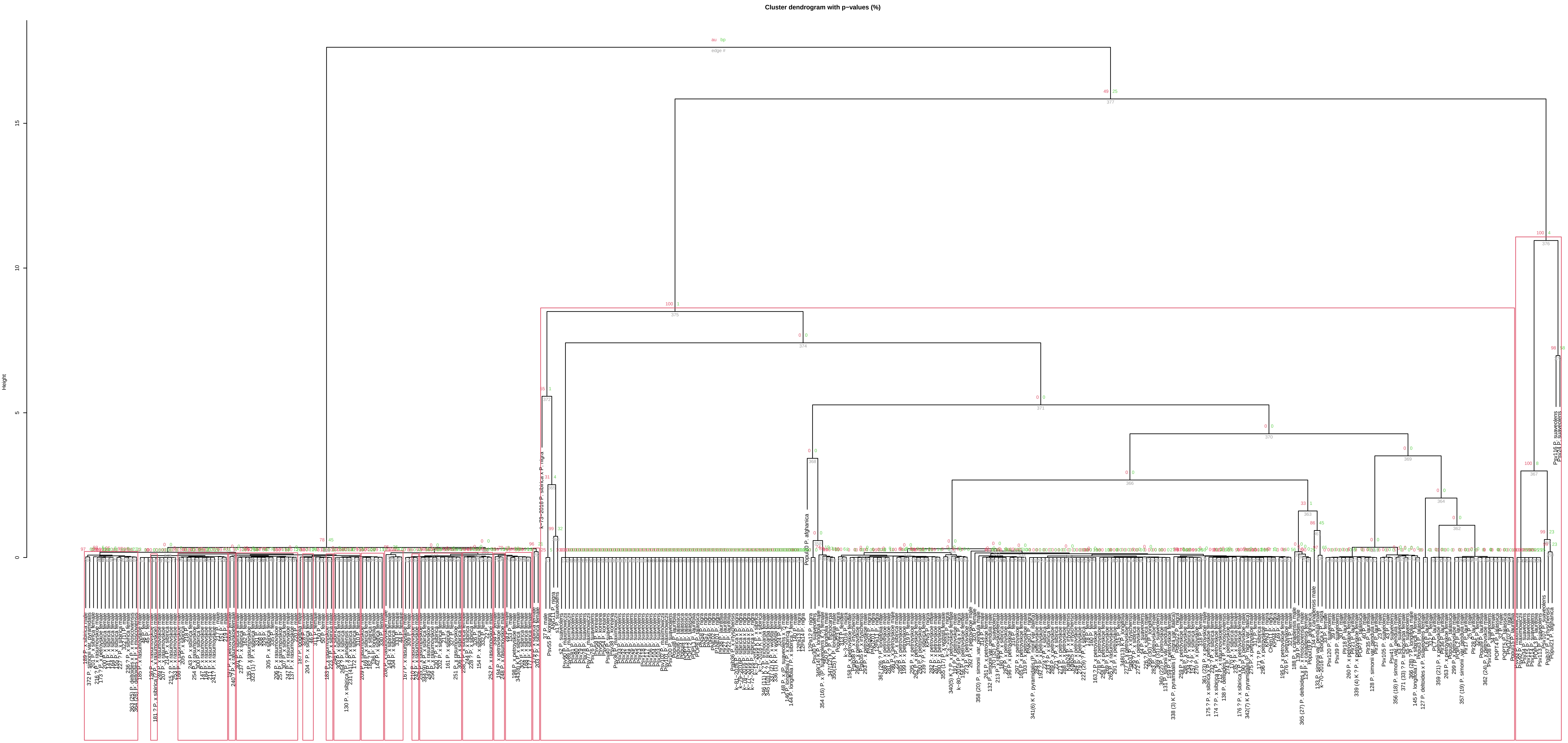

Supplementary Data 8K. Dendrogram based on deep sequencing data for *DSH 5* sequences.

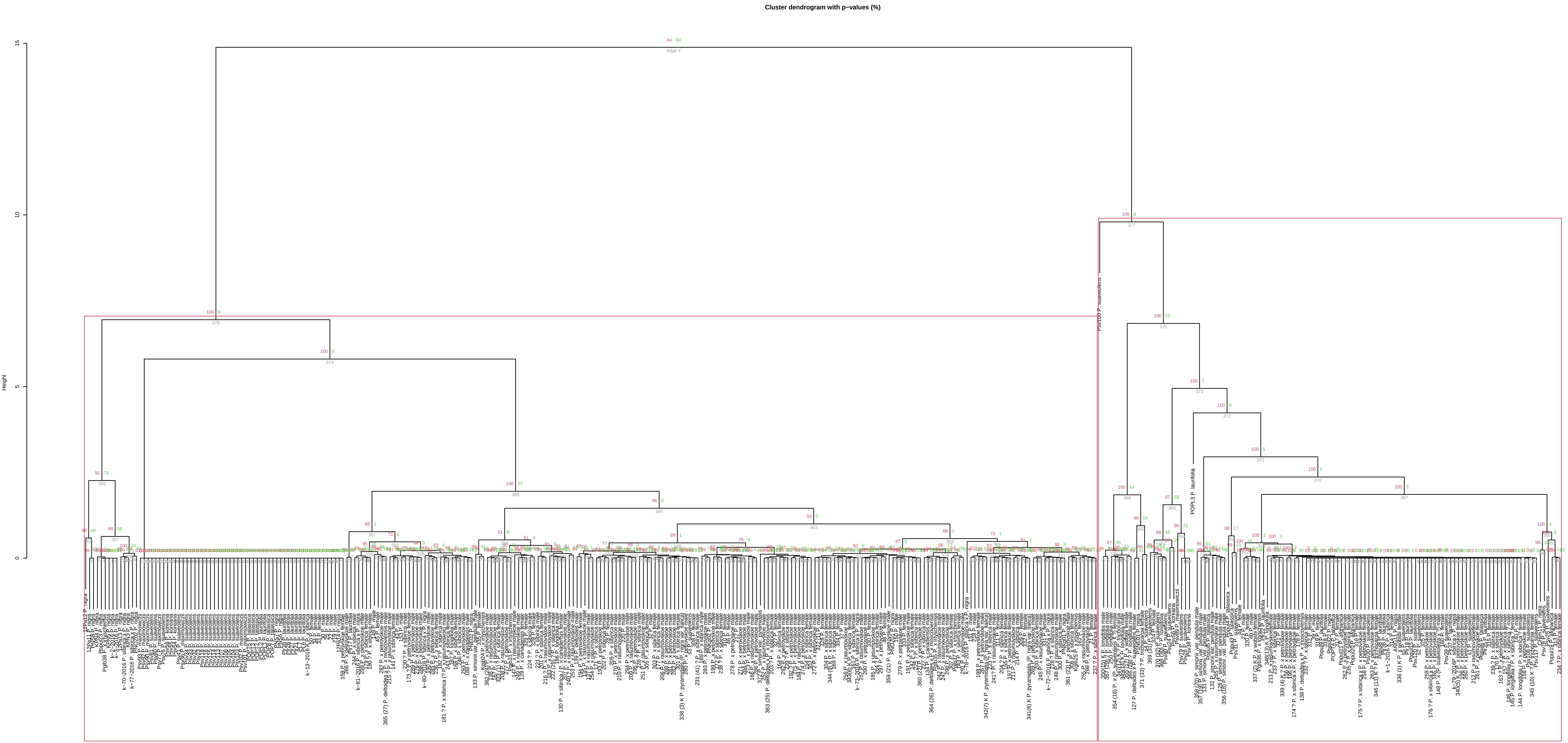

Supplementary Data 8L. Dendrogram based on deep sequencing data for *DSH 12* sequences.

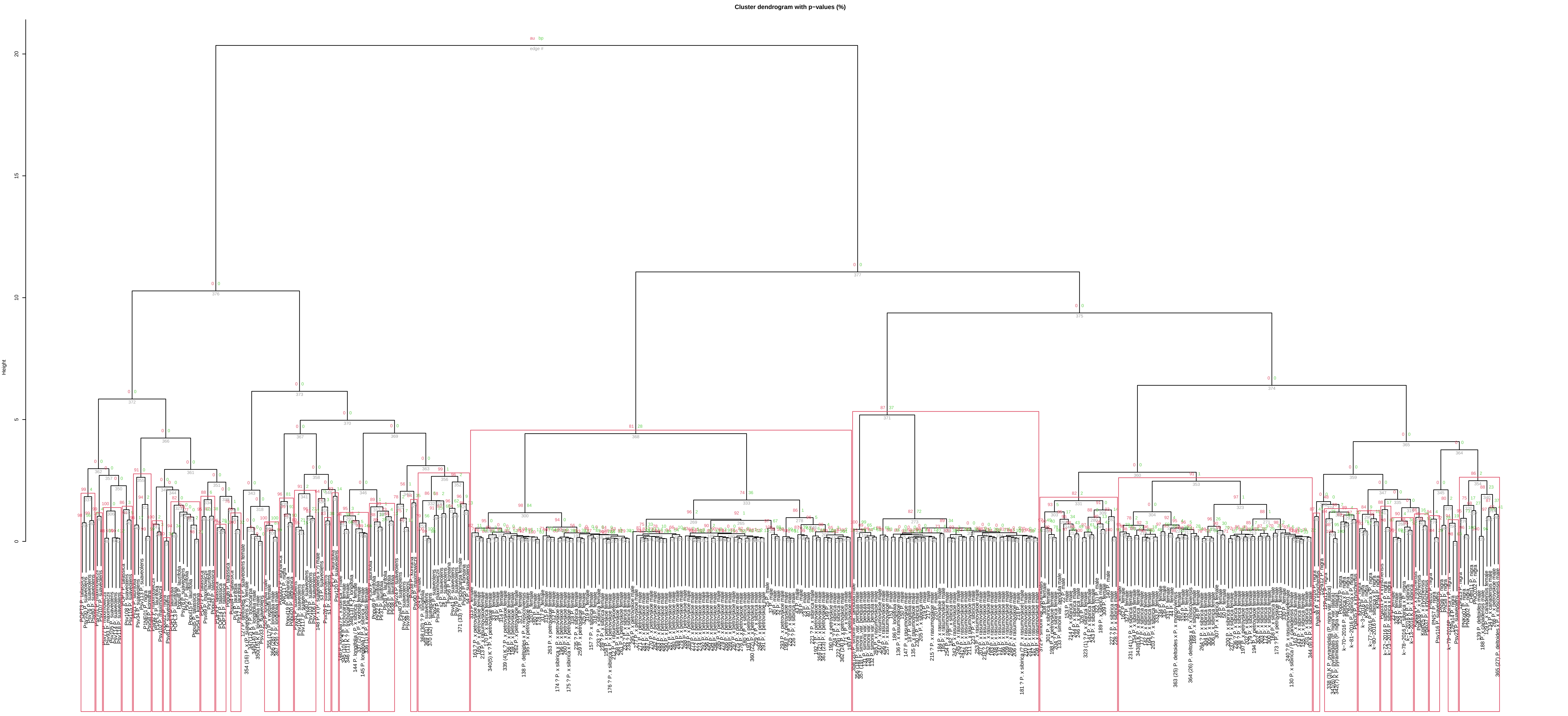

Supplementary Data 8M. Dendrogram based on deep sequencing data for NTS 5S rDNA, ITS, *DSH 2*, *DSH 5*, *DSH 8*, *DSH 12*, *DSH 29*, *6*, *15*, *16*, and *X18* sequences.

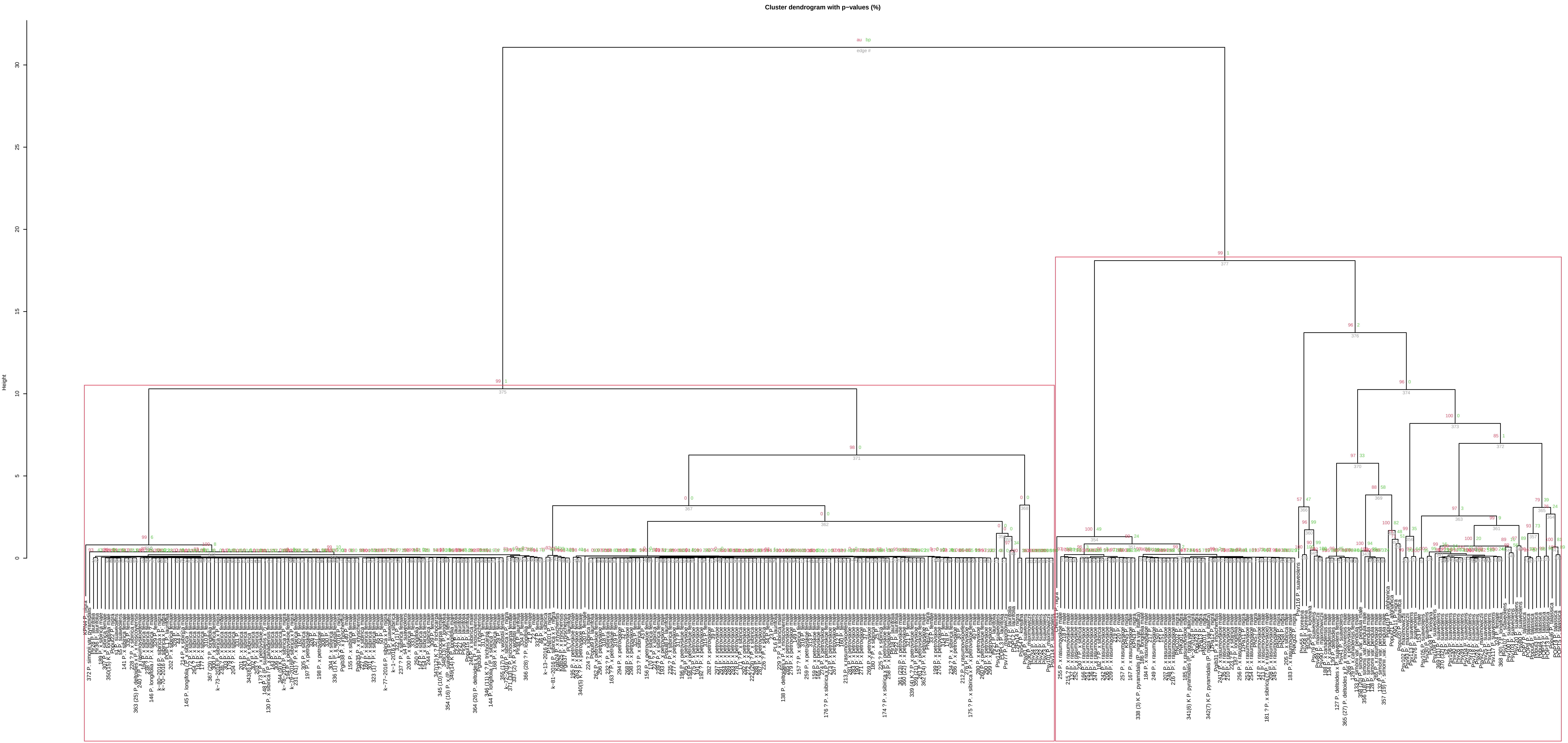

Supplementary Data 8N. Dendrogram based on deep sequencing data for *trnG-psbK-psbI* sequences.

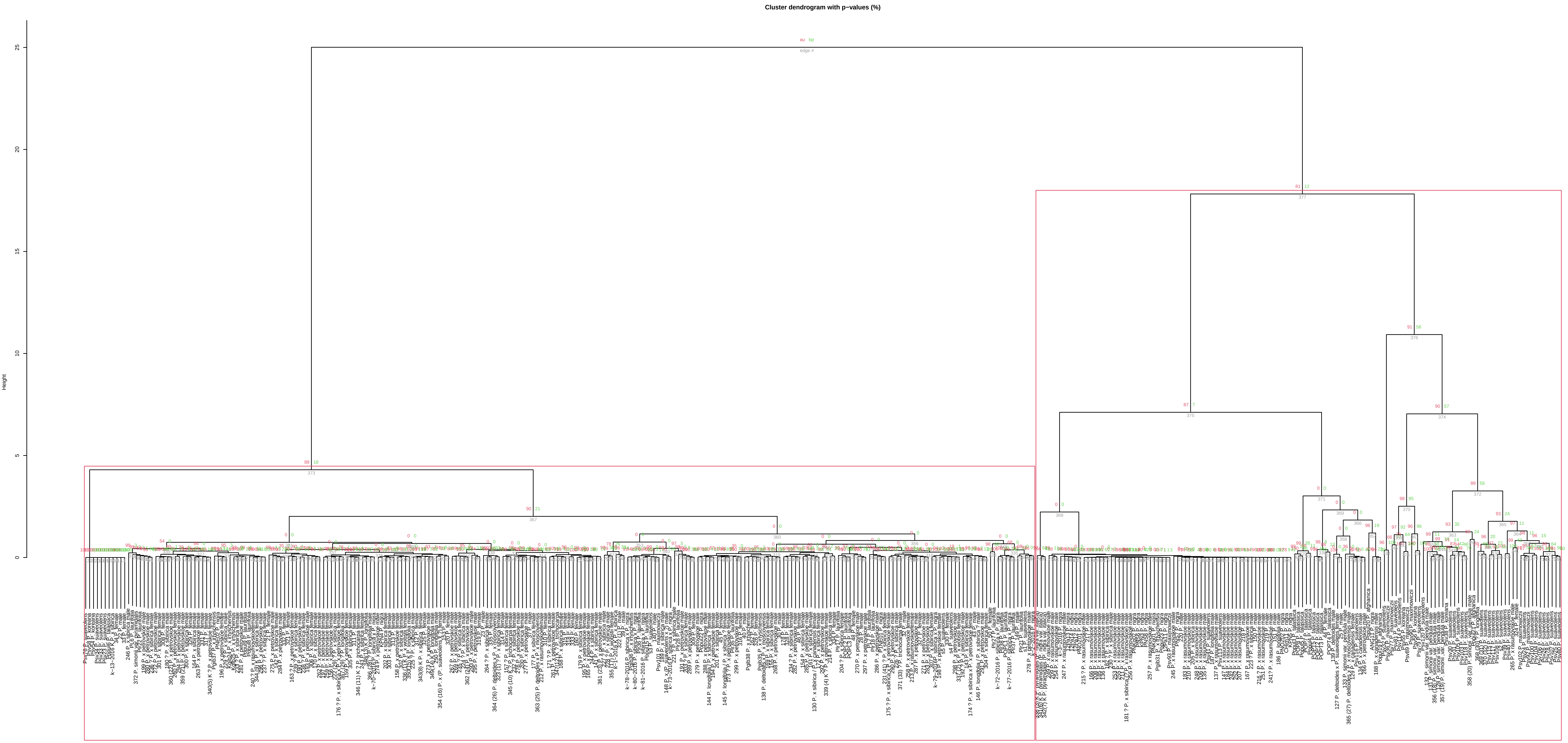

Supplementary Data 8O. Dendrogram based on deep sequencing data for *rps2-rpoC2* sequences.

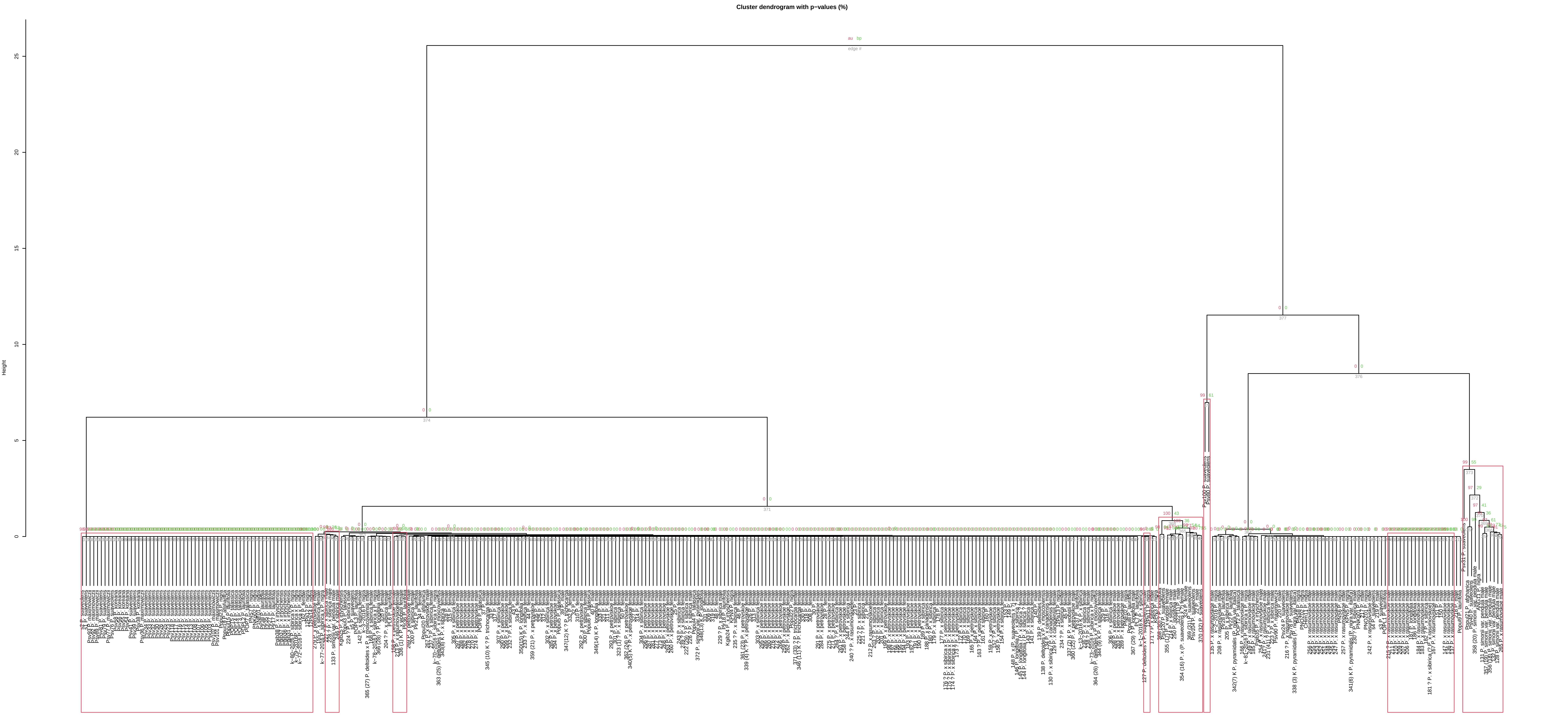

Supplementary Data 8P. Dendrogram based on deep sequencing data for *rpoC2-rpoC1* sequences.

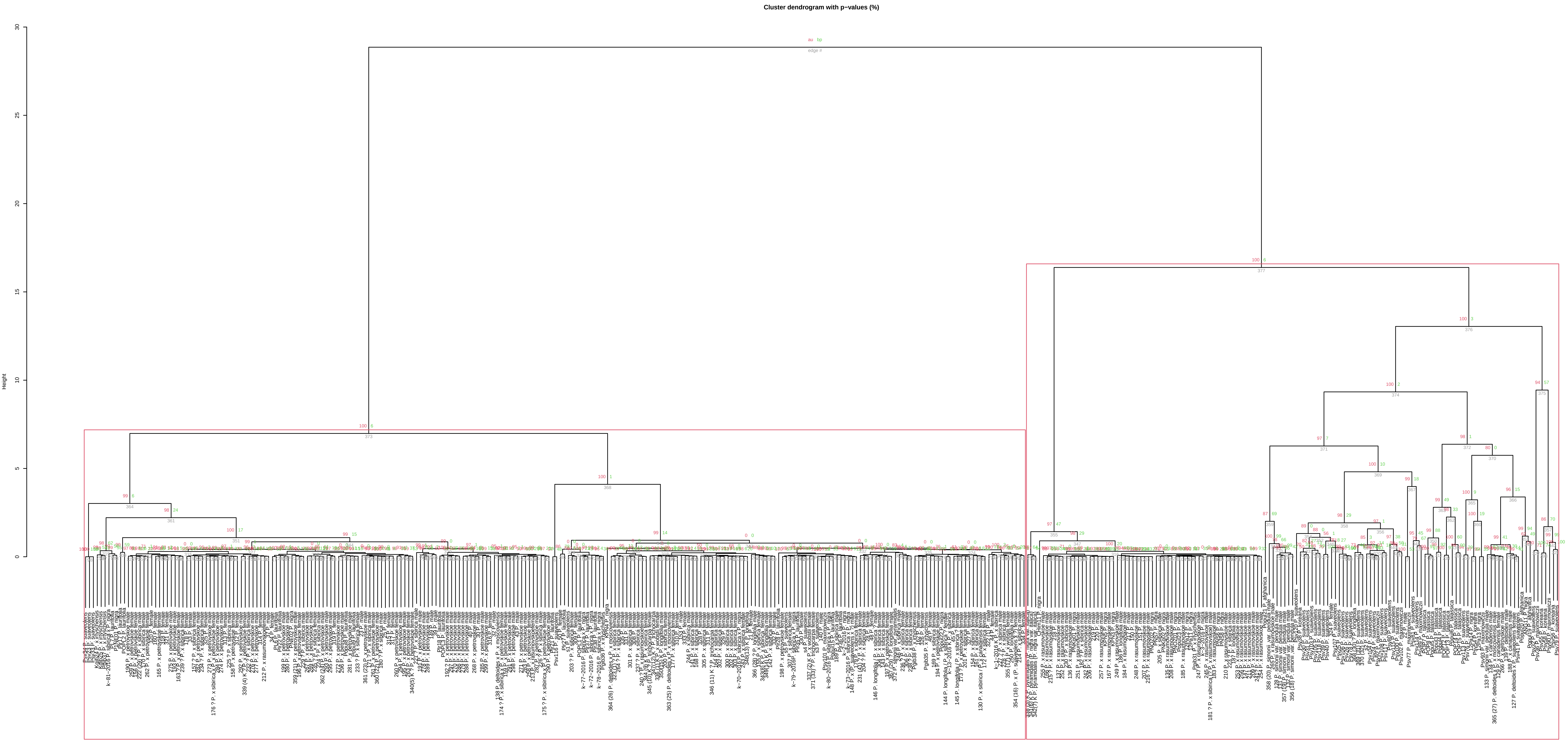

Supplementary Data 8Q. Dendrogram based on deep sequencing data for *trnG-psbK-psbI*, *rps2-rpoC2*, and *rpoC2-rpoC1* sequences.

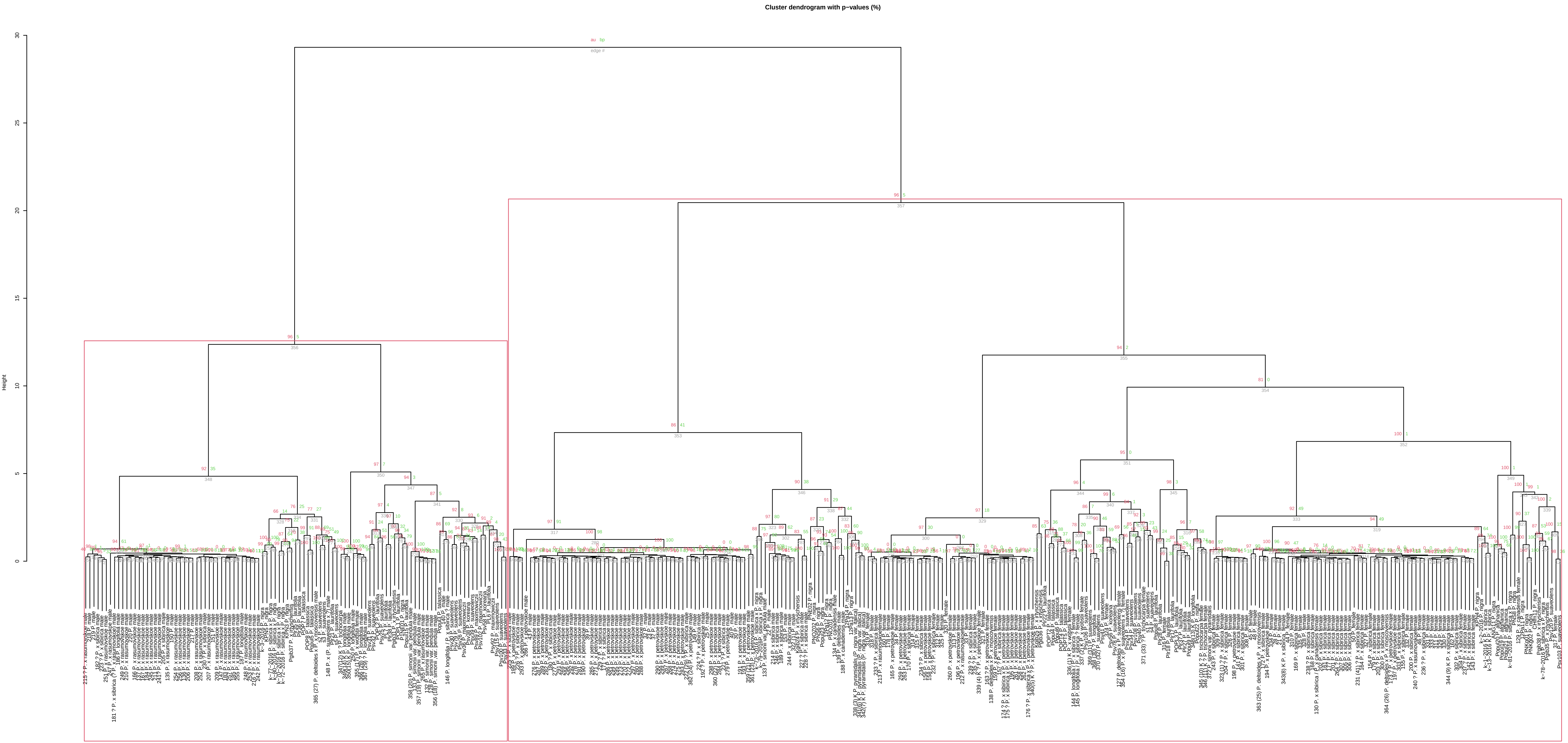

Supplementary Data 8R. Dendrogram based on deep sequencing data for the SDR sequences.

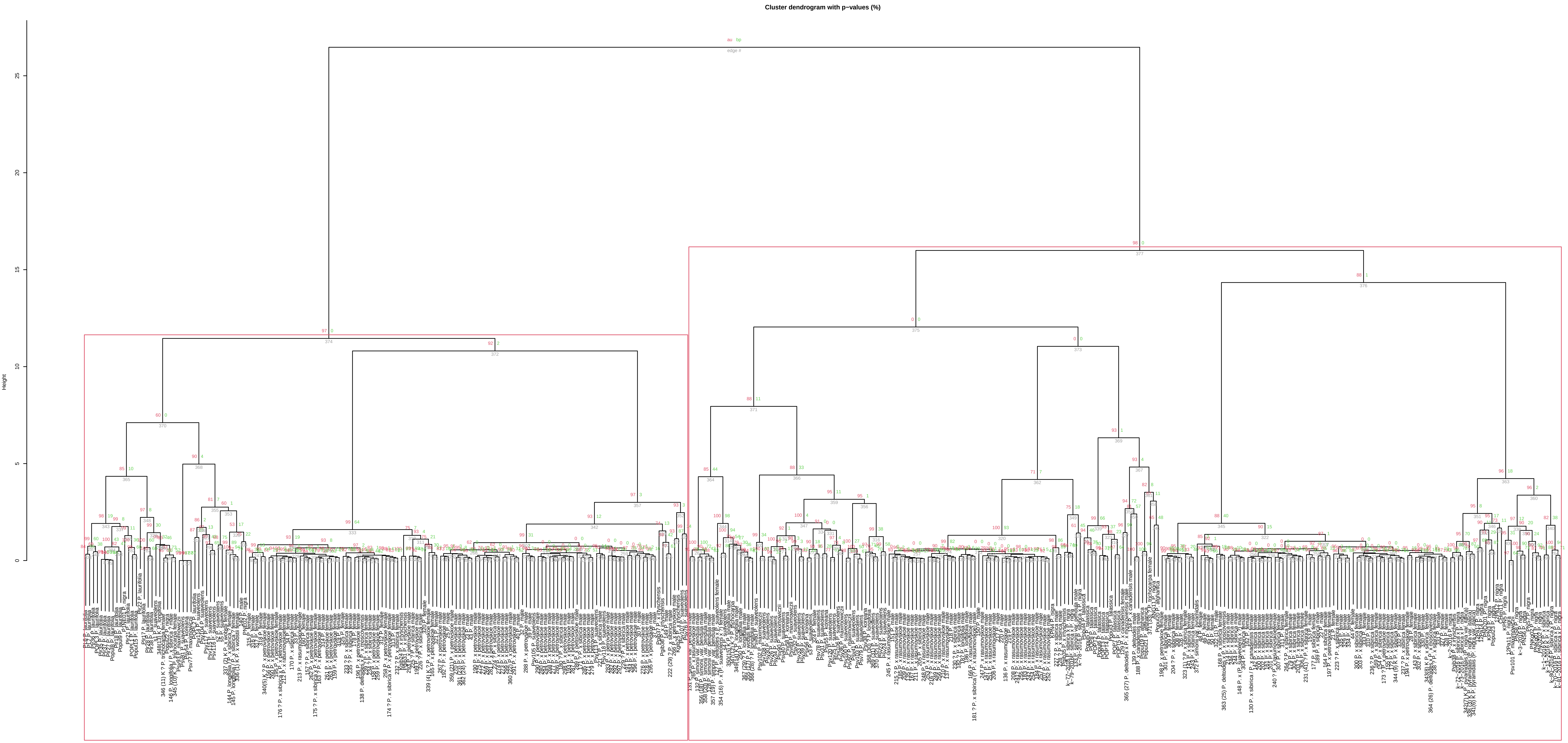

Supplementary Data 8S. Dendrogram based on deep sequencing data for the *ARR17* gene sequences.

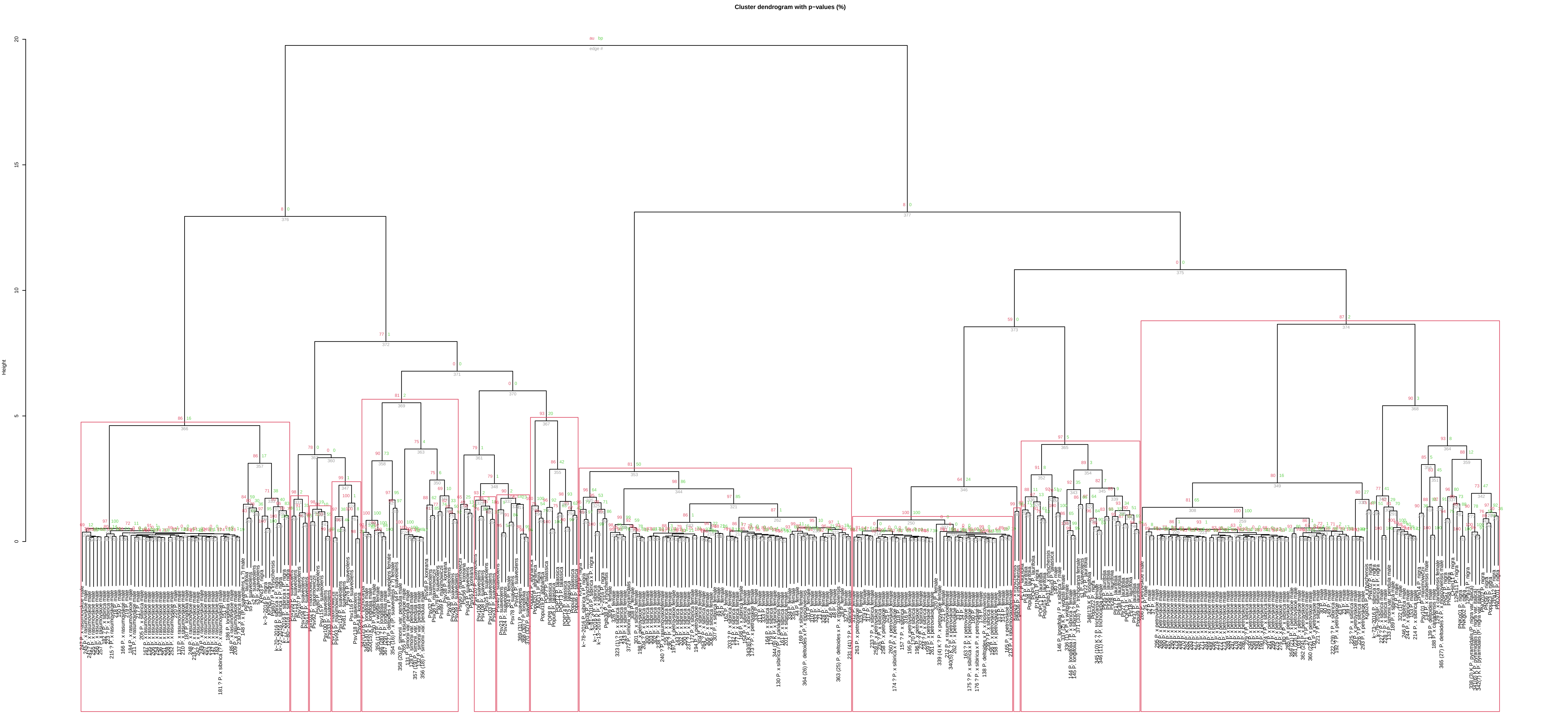

Supplementary Data 8T. Dendrogram based on deep sequencing data for NTS 5S rDNA, ITS, *DSH 2*, *DSH 5*, *DSH 8*, *DSH 12*, *DSH 29*, *6*, *15*, *16*, *X18*, *trnG-psbK-psbI*, *rps2-rpoC2*, *rpoC2-rpoC1*, SDR, and *ARR17* sequences.
